# Intron Evolution: Testing Hypotheses of Intron Evolution Using the Phylogenomics of Tetraspanins

**DOI:** 10.1371/journal.pone.0004680

**Published:** 2009-03-05

**Authors:** Antonio Garcia-España, Roso Mares, Tung-Tien Sun, Rob DeSalle

**Affiliations:** 1 Unitat de Recerca, Hospital Joan XXIII, Institut de Investigacio Sanitaria Rovira I Virgili (IISPV), Universitat Rovira i Virgili, Tarragona, Spain; 2 CIBER de Diabetes y Enfermedades Metabólicas Asociadas (CIBERDEM), Universitat Rovira i Virgili, Tarragona, Spain; 3 Department of Cell Biology, New York University School of Medicine, New York, New York, United States of America; 4 Department of Dermatology, New York University School of Medicine, New York, New York, United States of America; 5 Department of Pharmacology, New York University School of Medicine, New York, New York, United States of America; 6 Department of Urology, New York University School of Medicine, New York, New York, United States of America; 7 Sackler Institute for Comparative Genomics, American Museum of Natural History, New York, New York, United States of America; Centre de Regulació Genòmica, Spain

## Abstract

**Background:**

Although large scale informatics studies on introns can be useful in making broad inferences concerning patterns of intron gain and loss, more specific questions about intron evolution at a finer scale can be addressed using a gene family where structure and function are well known. Genome wide surveys of tetraspanins from a broad array of organisms with fully sequenced genomes are an excellent means to understand specifics of intron evolution. Our approach incorporated several new fully sequenced genomes that cover the major lineages of the animal kingdom as well as plants, protists and fungi. The analysis of exon/intron gene structure in such an evolutionary broad set of genomes allowed us to identify ancestral intron structure in tetraspanins throughout the eukaryotic tree of life.

**Methodology/Principal Findings:**

We performed a phylogenomic analysis of the intron/exon structure of the tetraspanin protein family. In addition, to the already characterized tetraspanin introns numbered 1 through 6 found in animals, three additional ancient, phase 0 introns we call 4a, 4b and 4c were found. These three novel introns in combination with the ancestral introns 1 to 6, define three basic tetraspanin gene structures which have been conserved throughout the animal kingdom. Our phylogenomic approach also allows the estimation of the time at which the introns of the 33 human tetraspanin paralogs appeared, which in many cases coincides with the concomitant acquisition of new introns. On the other hand, we observed that new introns (introns other than 1–6, 4a, b and c) were not randomly inserted into the tetraspanin gene structure. The region of tetraspanin genes corresponding to the small extracellular loop (SEL) accounts for only 10.5% of the total sequence length but had 46% of the new animal intron insertions.

**Conclusions/Significance:**

Our results indicate that tests of intron evolution are strengthened by the phylogenomic approach with specific gene families like tetraspanins. These tests add to our understanding of genomic innovation coupled to major evolutionary divergence events, functional constraints and the timing of the appearance of evolutionary novelty.

## Introduction

Eukaryotic protein coding genes are interspersed with non coding sequences called introns that are removed from the corresponding transcripts by the spliceosome, a complex RNA-protein assemblage. Introns and sequences of proteins from the splicing machinery have been found in all eukaryotic species with fully sequenced genomes [1]–[3]. Despite the vast amount of information generated since their discovery and the importance of introns in understanding gene organization, many issues regarding intron evolution remain enigmatic. These issues include the timing of intron origin and proliferation, the evolutionary history of introns and mechanisms of intron loss/gain in eukaryotic organisms, and the evolutionary dynamics that can explain their existence. These issues have led many researchers of intron biology to ask - is there a selective advantage to having introns and if so what is the advantage [for recent reviews see: 3]–[7].

Studies on the evolution of spliceosomal introns mainly use broad genomic data sets of conserved homologous genes from diverse eukaryotic organisms [3], [4], [8]–[10]. Few publications have addressed intron evolution by examining full complements of a gene family and the distribution of intron/exon sites in all members of a family, probably because the intron-exon structure was only known for a small set of species [6], [11]–[14]. As pointed out by Hughes our understanding of protein evolution could be improved by studying specific well characterized systems [15]. The recently fully sequenced genomes of multiple eukaryotic species covering broad evolutionary divergences, makes analysis of intron-exon structure of individual gene families an interesting option. In particular, taking a phylogenomic approach to understand the distribution of intron/exon evolution in a suitable gene family would allow the determination of ancestral states of intron presence/absence, and allow for the correlation of intron loss/gain events with function and to place time estimates on intron/exon evolutionary events.

We suggest that suitable gene families to apply the phylogenomic approach to examine intron/exon structure would be ones with many members, several introns in each paralog and a broad phylogenetic distribution. The tetraspanin superfamily of proteins meets all three of these important requirements. This large family has 33 paralogs in the human genome and at least 37 members in *Drosophila* [16]. Members of the family are found in eukaryotic organisms as diverseas animals, fungi, plants and protists [17]–[18].

**Figure 1:**
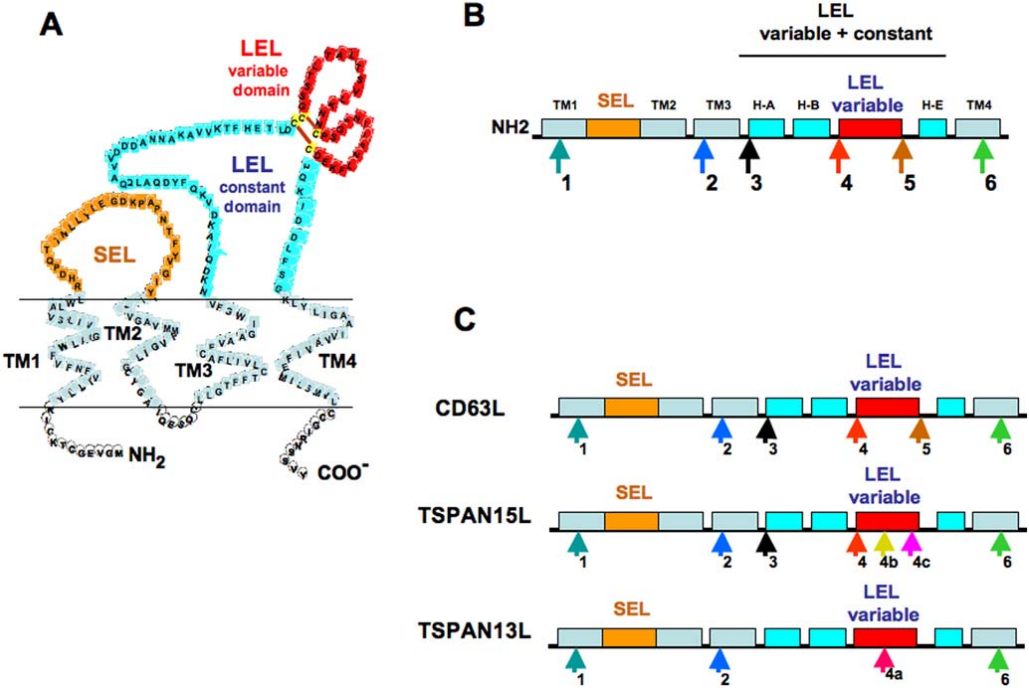
Cartoon of a typical tetraspanin and intron positions. (A) The small (SEL) and large (LEL) extracellular loops constant and variable regions are indicated. The example shown is that of CD81, whose LEL 3D structure has been solved (43). Brown bars in the LEL loop represent disulfide bridges; Orange, colored SEL; Dark blue, constant region of LEL; Red, variable region of LEL; Light blue transmembrane domains; no color, intracellular regions. (B) Ancestral intron positions 1 to 6 are indicated on the protein by arrows of the same color that will be used through all of the figures. TM-1 to 4 transmembrane domains; H-A, H-B and H-E constant helices in the LEL (C) Animal CD63L, TSPAN15L and TSPAN13L tetraspanins' consensus intron structure.

The biochemical functions of tetraspanins, a broadly expressed superfamily of transmembrane proteins, are based upon their ability to form large integrated signalling complexes or tetraspanin-enriched microdomains by their primary associations with multiple transmembrane and intracellular signaling/cytoskeletal proteins and secondary associations with themselves [19], [20]. Tetraspanins participate in many membrane-associated cellular activities such as cell adhesion, motility, activation of signaling pathways, and cell proliferation. This participation occurs in normal and in pathological conditions such as cancer metastasis or infections by viral, bacterial, or parasitic organisms [21]–[30]. Specific functions have been described for some tetraspanins such as the PLS1 tetraspanin, which enables the plant pathogenic fungus Magnoporthe to invade its rice host's leaves [31]; the LBM tetraspanin, whose mutations cause synaptic defects in *Drosophila*; the CD9 and CD81 tetraspanins, which are involved in mammalian sperm: oocyte fusion [25], [30]; CD81, which is involved in immune signaling [32]; peripherin/RDS, which scaffolds vertebrate photoreceptor outer segment structure [33]; and uroplakins, in the maintenance of the urothelial permeability barrier [34]–[36]. The ability of tetraspanins to carry out multiple interactions relies upon their characteristic structure. Structurally, tetraspanins are proteins of only 200–300 amino acids, with four conserved transmembrane domains which delimit one small extracellular loop of 13–31 aminoacids (SEL; see Figure 1), a short intracellular sequence and a large extracellular loop of 69 to 132 aminoacids (LEL) [37], [38]. These SEL and LEL regions are the least conserved regions of tetraspanins. The LEL has two domains: a constant region, containing three α-helices (H-A, H-B, and H-E) without cysteines and a variable region, characterized by the presence of cysteines that form a structural scaffold of disulfide bridges that allows for high sequence variability on the inter-cysteine loops (Fig 1A, B) [39]. This variable region in the LEL contains nearly all of the known tetraspanin protein-protein interaction sites and most likely specifies the diverse tetraspanin classes and functions [23], [37].

The typical intron/exon structure of most tetraspanins has been reported, in bilaterian animals, to consist of six introns in positions that do not break the reading frame (so called phase 0 introns) that we have named introns 1 to 6 (Fig. 1 B) [40].

In the present study we add to our recent analyses of tetraspanin relationships [18], [41] by investigating exon/intron evolution. In our previous study we utilized information from tetraspanin paralogues from fully sequenced genomes (bilaterian animals - protostomes and deuterostomes, plants - monocots and dicots, fungi - Microsporidia, Zygomycota and Asmcycota, and protists - Amoebozoa and Excavates [18]). For the present study, we add tetraspanin paralogues from non bilaterian animals (Cnidarians, Placozoa and Poriferans), Choanoflagellates (the closest unicellular relatives of animals), Fungi (chitridiomicota), Plants (lycophytes and mosses) and Protists (stramenopiles, alveolates and discicristates). In all, these organisms cover seven out of the eight major groups of eukaryotic organisms [42]. This analysis of the full complement of tetraspanins in a broad set of eukaryotic organisms allowed us to precisely pinpoint the origin of specific exon/intron structure and to determine the evolutionary significance of intron gain/loss events.

With a broad taxonomic sampling of tetraspanin genes and precise description of their exon/intron structure we can test several hypotheses relevant to the evolution of tetraspanin introns. First, we can examine whether the tetraspanins corroborate already well-established patterns of exon/intron loss in other genes. Because there are several introns in the tetraspanins this allows us to test hypotheses of intron gain/loss very precisely. Second, we hypothesize that major structural changes in tetraspanin genes with respect to exon/intron structure are associated with major cladogenetic events in the eukaryotic tree of life. This hypothesis stems from ideas about major radiations of organisms being accompanied by similar major adaptations. In order to test this hypothesis, we reconstruct the gain/loss of introns on a well corroborated eukaryotic tree and examine where on the tree the gain/loss events occur. Third, we hypothesize that substantial exon/intron structural changes that occur in tetraspanins are associated with major functional changes in these proteins. This hypothesis can be tested by examining whether any other physical aspects of tetraspanins change concurrently with exon/intron alterations. Specifically, we examine if changes in the cysteine motifs in tetraspanins are coincidental or correlated with exon/intron changes.

## Results

### Sequence analysis of sponge tetraspanin introns and the discovery of three novel ancient animal tetraspanin introns

**Figure 2:**
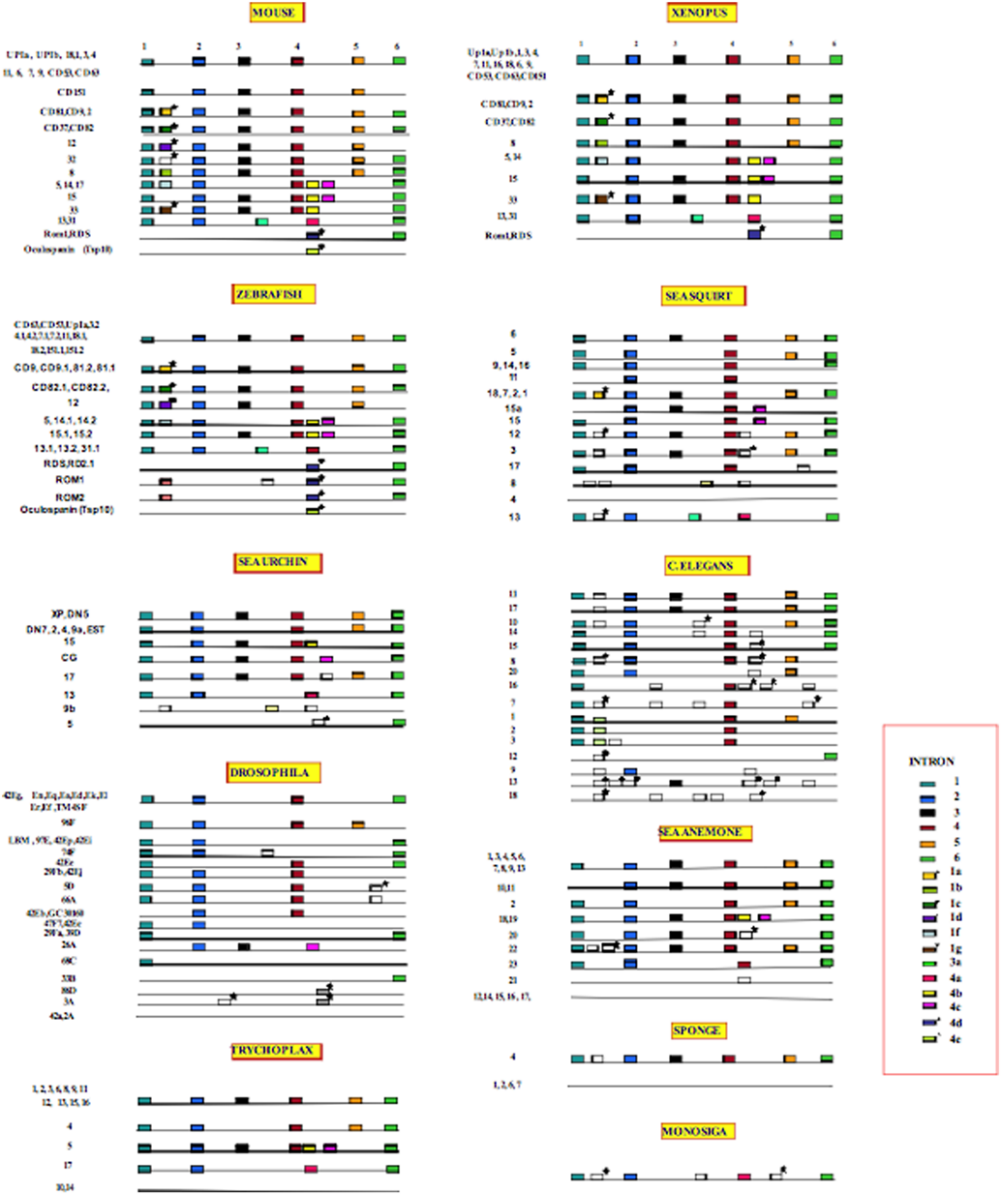
Intron/exon structure of all animal tetraspanins in the present study. Intron positions are represented by boxes of different colors. Ancestral introns 1–6 are numbered on top of the figure. Same color boxes represent conserved intron position. Empty boxes indicate unique intron positions within the species gathered in this analysis. A star above a box indicates an intron position that breaks the reading frame (intron phases 1 or 2).

**Figure 3:**
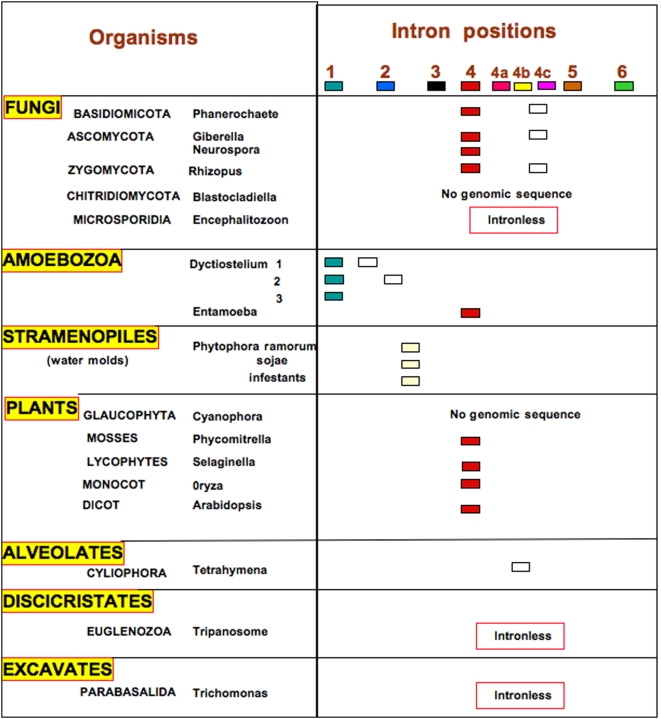
Intron/exon structure eukaryotic non-animal (fungi, plants and protists) tetraspanins in the present study. Intron numbering and colours are as in legend of Figure 2.

Sponges are often considered the most primitive diploblastic (two tissue layers) animals. Since fully sequenced sponge genomes are not yet available, we searched the NCBI database for sponge tetraspanins in the expressed sequence tag (ESTs) database. We identified several tetraspanins from the sponge *Oscarella carmella* ESTs, designed primers to obtain introns and determined the exon/intron structure by sequencing *O. carmella* genomic DNA. All six ancestral intron positions were present in one of the five sequences of *O. carmella* tetraspanins that we obtained (Fig 2, Table S1). While we did not detect other intron/exon boundaries in *Oscarella*, we cannot infer that these do not exist, as the tetraspanins from this organism were not obtained from completed genome sequences. Mining of the whole genomes of *Nematostella* and *Trichoplax* (Placozoa) for tetraspanins revealed the presence of all six introns in the tetraspanins of these diploblastic animals. We found ancient intron 4 also in Fungi, Plants and introns 1 and 4 in Amoebozoa (Figure 3). In addition to the six reported ancestral introns, 1 to 6, we identified three new ancient introns we call 4a, 4b, and 4c, which are conserved from the ancestors of the non bilaterian animal, Placozoa (*Trichoplax adherens*, introns 4b and 4c) and the unicellular choanoflagellate (*Monosiga*; intron 4a) to mammals (Fig. 2, Figures S1, S2 and S3). All these nine introns are in positions that do not break the reading frame (phase 0 introns).

### Animal tetraspanins cluster in three ancient groups by their intron/exon gene structure

We observed that three combinations of the nine ancient introns (1 to 6, 4a, 4b, and 4c) divide animal tetraspanins into three groups; we call them CD63-like (CD63L) (introns 1, 2, 3, 4, 5 and 6), TSPAN15-like (TSPAN15L) (introns 1, 2, 3, 4, 4b, 4c and 6) and TSPAN13-like (TSPAN13L) (1, 2, 4a and 6) (Fig 1 C and Figs S1, S2, and S3). These three groups of tetraspanins have been conserved from the basal non-bilaterian metazoans (i.e., placozoans and sea anemones (Figure 2 and Figures S1,S2, and S3). Introns 1, 2, and 3 are common to each of the three groups described above; interestingly variant introns 4, 4a, 4b, 4c and 5 are located in the region of tetraspanin genes that codes for the variable LEL region. (Figure 1C and Figures S1, S2, and S3). This protein region, involved in protein-protein recognition between tetraspanins and other proteins [23], [37] also includes specific cysteines that form a different scaffold of disulfide bonds in each of these three groups (CD63L, TSPAN15L and TSPN13L), allowing for high sequence variability in these regions. The cysteine specific disulfide bonds are predicted for the CD63L and TSPAN15L groups in Kitadekoro et al, [43] and Signeuret et al [39]. The TSAPAN13L prediction was generated using the DiANNA web server [44] (Data not shown). Introns 1 to 6 alone, characterize the largest group of tetraspanins, CD63L. Tetraspanins in this group have six cysteines in the LEL with the pattern: CC–CC–C–C (where C means cysteine,; and dashes represent a variable number of amino acids; Figure S1). Tetraspanins in the TSPAN15L group have introns 1, 2, 3, 4, 4b, 4c and 6. This TSPAN15L tetraspanin group has an eight-cysteine pattern: CC–C–C–CC–C–C in the LEL variable region (Suppl. Fig. 2). The last group (TSPAN13L) has introns 1, 2, 4a and 6, and six cysteines in the following order: CC–C–C–CxxC (where x refers to any amino acid residue; Figure S3). In this group, a new intron we call 3a, appeared in the common ancestor of tunicates (the sea squirt *Ciona intesinallis*) and vertebrates and has been conserved since (Figure S3). In animals, new classes of tetraspanins appeared during the diversification of phyla and classes, some of the new tetraspanins, although maintaining the intron structure in genomic DNA, that codes for the LEL variable region have variation in their cysteine patterns. Such is the case of tetraspanins CD151, CD53, CD9, CD81, CD82, CD37 and TSPAN11, TSPAN 9, TSPAN 4, TSPAN 2, TSPAN 8, TSPAN 1, TSPAN 16, TSPAN18 that have a variable number of cysteins (4, 6, or 8) and constant introns 4 and 5 in the DNA sequence that codes for the LEL region, (Figure S4)

### A tetraspanin found in the Unicellular Choanoflagellate Monosiga belongs to TSPAN15L

A search for tetraspanins in the recently sequenced genome of the choanoflagellate, *Monosiga brevicolis*, a unicellular organism, which is considered the closest relative to multicellular animals [45], revealed a single tetraspanin sequence with the intron characteristics of the TSPAN15L group (introns 1, 2, 4a and 6; Figure 2 and Figure S3). In addition to the introns described above, *M. brevicollis* has four additional specific introns (Figure 2; Figure S3).

### Tracing tetraspanin intron loss/gain to common ancestors in the eukaryotic tree of life

**Figure 4:**
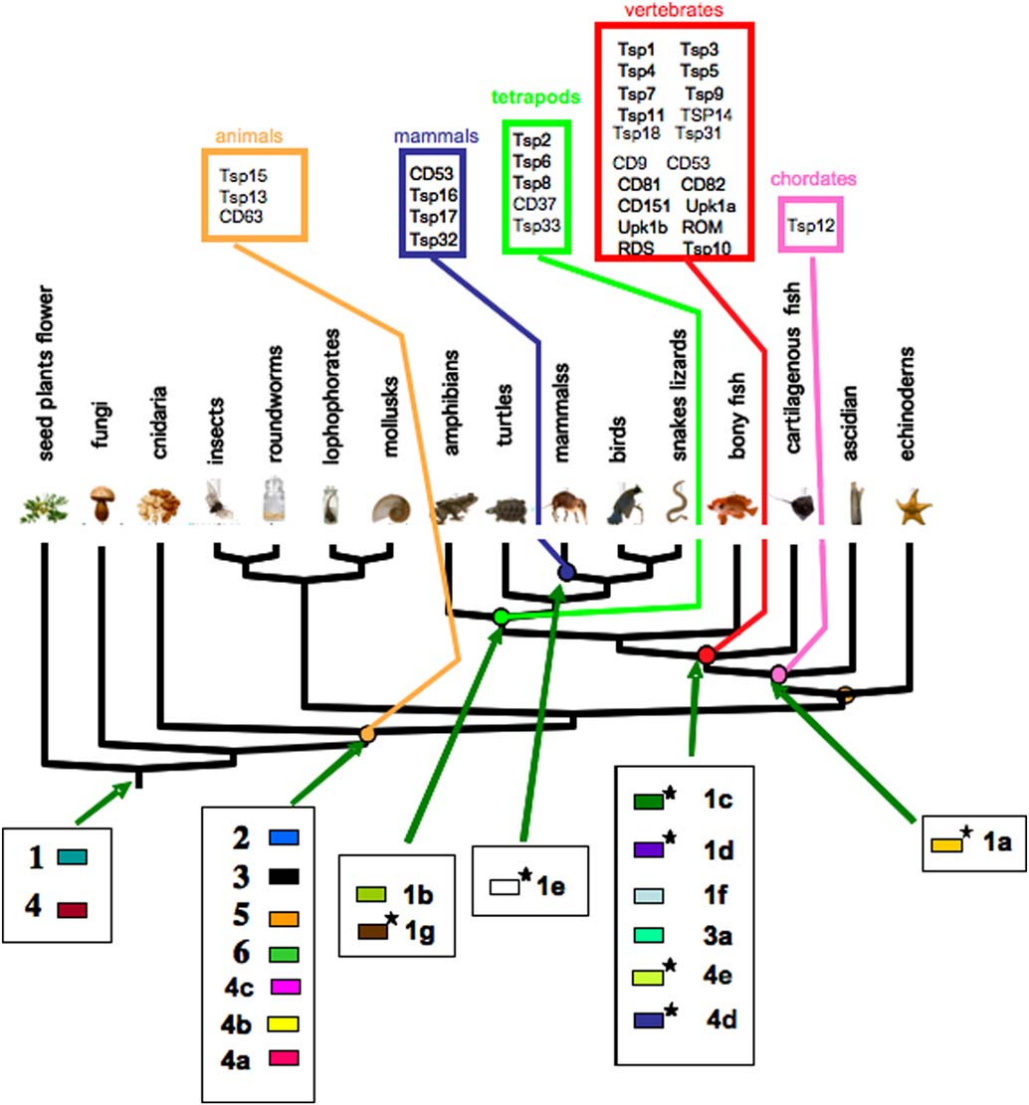
Ancestral origins of introns in mice and humans. The tree shows ancestral points of origin (mammals, tetrapods, vertebrates, chordates and animals) for the human and mouse tetraspanins (modified from Garcia-Espana et al, 2008 (18). Human and mouse intron origins are indicated by arrows. The phylogenetic tree is based on our best recent understanding of relationships of major taxonomic groups.

Phylogenomic analysis, performed using a multigene family like the tetraspanins from several organisms, can allow for the identification of the common ancestor of an intron loss/gain event. The phylogenetic distribution of the origins of the 19 introns that have been conserved in the 33 human tetraspanin paralogs is shown in Figure 4.

In the following we refer to any intron other than 1 thru 6 and 4a, 4b and 4c as derived because they have arisen in more derived common ancestors. Of the ten derived introns in human tetraspanins most arose in the common ancestor of the vertebrates, where six new introns can be traced to this common ancestor. In addition, two new introns can be traced to the common ancestor of mammals. These observations suggest that, for the sparsely sampled organisms we examined in this study, there are very few cases of intron loss/gain in single species lineages suggesting that most intron evolutionary events in tetraspanins coincide with major radiations of animals. The exceptions to this observation are the nematode tetraspanins, where intron gain/loss is rampant in that species (see below).

We also observed that of the ten more highly derived intron positions we detect in this study, seven break the reading frame (either phases 1 or 2), in contrast to the ancestral nine phase 0 well-conserved introns (introns 1 through 6). These phase 1 or 2 introns are found in tetraspanin groups: CD9, Tsp2, CD37 (intron 1a); Tsp8, (intron 1b); CD82, CD37, (intron 1c); Tsp 12 (intron 1d); Tsp32, (intron 1e); ROM, RDS, (intron 4d); and Tsp10, (intron 4e). For example, the gene structure of the tetraspanin subgroup Tsp2/CD81/CD9 shows a new intron (intron 1a in Figure 2, Figure S4) between intron 1 and 2. This intron (1a) has been conserved from tunicates (*Ciona*) throughout vertebrates. Another example is that of the CD37/CD82 subgroup which has accrued a new intron (intron 1c) in the ancestor of vertebrates (Figure 2, Figure S4).

### Frequency of intron gain/loss in tetraspanins

**Figure 5:**
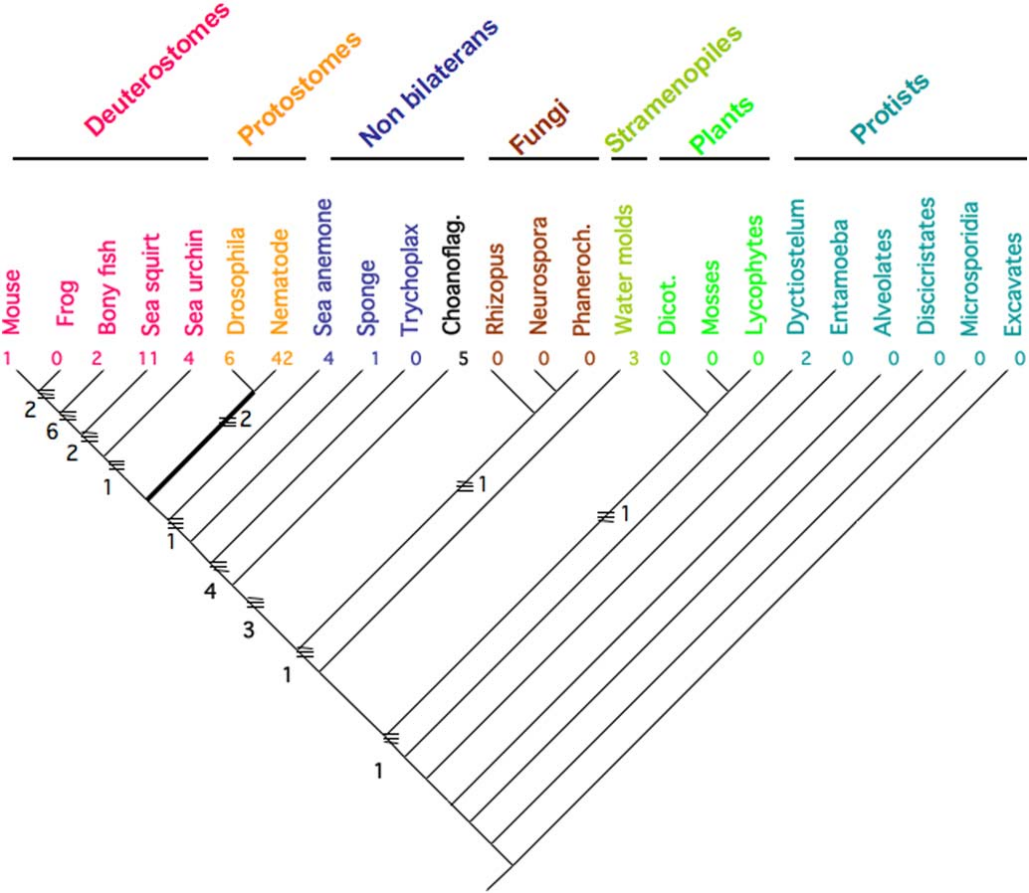
Phylogeny of all organisms examined in this study showing number of intron gains and losses on internal branches. The number of gains and losses in a particular species since its divergence from a common ancestor is shown below the taxon name. For instance, the number four below the sea urchin name means that the sea urchin has had four gain/loss events since its divergence from the common ancestor of sea urchins and vertebrates.

We coded all introns, in all organisms we examined in this study as present, absent or as unknown (missing data) into a matrix [46], [47], and mapped these onto the phylogenetic hypothesis in Figure 5. The result of character mapping and ancestral reconstruction indicated that nine conserved ancient intron/exon junctions (1 thru 6 and 4a, 4b and 4c) were present in the ancestor of all animals. The red branches in Figure 5 show the position and number of intron gains that led to the nine ancestral animal tetraspanin introns. Subsequent gain and infrequent loss of intron/exon junctions has occurred in all protostome and deuterostome genomes we examined in this study (Figure 5).

Using the mapped presence/absence of introns on the phylogenetic tree we calculated there are 105 intron gain events and only four intron loss events. More than one fourth of these gains (27 gains) are in hypothetical ancestors in the tree. The majority of the gains occur in a single species, in the nematode *C. elegans* (42 gains), with ten percent occurring in the sea squirt (11 gains) and over five percent occurring in *Drosophila* (6 gains) and choanoflagellate (5 gains). The rest of the gains are dispersed across the other taxa. The four losses occur in the common ancestor of protosotomes (2; blue branch in Figure 5), in the lineage leading to *C. elegans* (1) and in the sea squirt (1).

**Figure 6:**
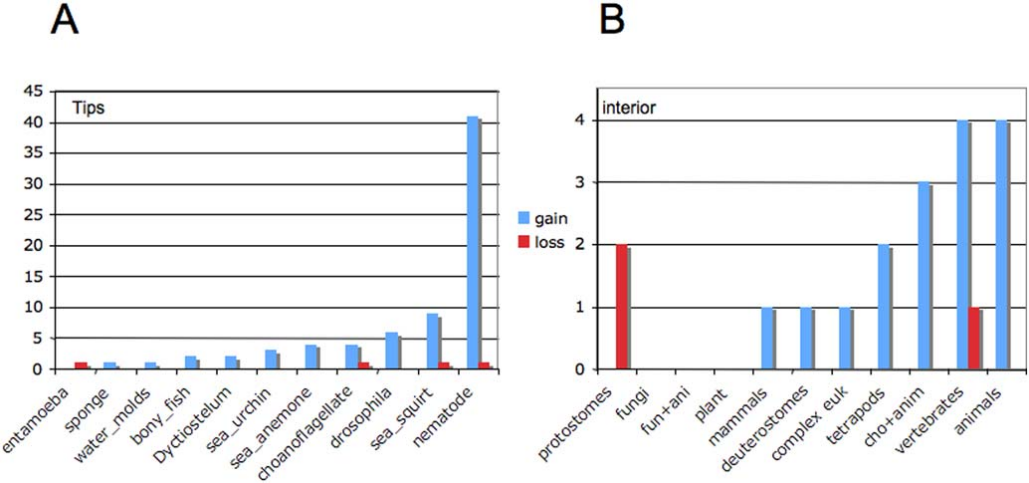
Histogram showing a comparison of the number of introns in taxa that are gains and losses at the tips of the tree (A) and at internal nodes of the tree (B). The number of events was calculated using the ACCTRANS option in MacClade. Common names of the species and higher categories of organisms are given on the X axis. The number of intron gains and losses are given on the Y axis. Gains are in blue. Losses are in red.

When we characterized the loss and gain of introns in tetraspanin genes in terminal lineages in the taxa in Figure 5 by calculating the percentage of introns that are lineage-specific gains and losses, we observe a high degree of variability of percentage of gains from taxon to taxon (Figure 6). The majority of taxa in Figure 2 have gained 10% or fewer introns in their tetraspanin genes since divergence of the last common ancestor in the tree. The only exceptions are the *C. elegans* tetraspanins, which show a very high frequency of intron gain (88% of the introns in *C. elegans* are gains from the common ancestor of *Drosophila* and *C. elegans*; Figure 6). The average number of introns per gene (not shown) is in accordance with what has been described for other genes (3; 7) with the exception of the *A. thaliana* tetraspanins which have fewer introns per gene than the average in that species. The high frequency of intron gains we observe in tetraspanins in *C. elegans* is a well documented phenomenon for other genes examined so far [48].

### Intron exon/structure in tetraspanins support several nodes in animal phylogeny

An examination of the phylogenetic patterns of intron presence/absence mapped on the phylogenetic tree (Figure 5) demonstrates a strong correspondence between phylogeny and the distribution of introns. The large scale agreement of intron presence and absence is manifest in the strong consistency of these characters when mapped on the tree. Only six introns have consistency indices less than 1.0, while of the 37 introns that are phylogenetically informative, 35 of these have a consistency index of 1.0, meaning they are entirely consistent with the well accepted phylogenetic hypothesis in Figure 5. These results suggest that major branching events in the tree of life (like the origin of vertebrates) are highly correlated with intron gain/loss. This result also implies that the tetraspanins that share the same intron structure cluster together into strongly supported phylogenetic groups. For example, in the large cluster that we call the CD group [Espana et al., 2007; 18], the subcluster CD151/Tsp11 and Tsp9/Tsp4/CD53 have the ancestral six intron structure, while CD9/Tsp2/CD81 all have, in addition, a new intron 1a between conserved introns 1 and 2. All tetraspanins in the Tsp8 group have intron 1b and CD82/CD37 tetraspanins have intron 1c (Figure S4).

### SEL and the hypervariable region of LEL in tetraspanins are preferential hotspots for new intron insertion

Interestingly, the derived tetraspanin introns are not randomly inserted in tetraspanin genes, but rather are preferentially found in the sequence region that codes for the small extracelular loop 1 (SEL) (Fig. 1). In this short domain that corresponds to only 10.5% of the total sequence surveyed, we found 36.7% of the 79 novel intron gains in tetraspanins (a ratio of 3.5 times greater rate than expected over random). The next highest intron dense region in tetraspanins is the variable subdomain in the LEL where 24.0% of all new introns are found in the 25.3% of the sequence that makes up the LEL. The remainder of the tetraspanin gene regions (the four trasmenmbrane domains and the LEL without the variable subdomain; Fig 1), had a frequency of intron insertions 5.8 times lower than that of the SEL (a frequency of 0.60 for each region). In particular, of the ten derived introns present in human tetraspanins (1a–f, 3a, 4d and 4e; Fig. 4), seven (1a to 1f) were found in the SEL region and three (3a, 4e and 4d) in the LEL region. A search for the implication of these derived introns in alternative spliced transcript in the NCBI AceView database, showed that only intron/exon junctions, 1f in TSPAN14 and TSPAN5, were involved in alternative splicing events (exons between introns 1f and intron 4 and between 1f and 2 were omitted in splice variants of TSPAN14 and TSPAN5 respectively; AceViev database TSPAN14 transcript variant iApr07 and TSPAN5 transcript variant bApr07).

### Apparent intron sliding in tetraspanins CD81 and Tspan15 is due to variation in sequence length at the ends of exons

As expected, the intron/exon junctions for the same intron (i.e., intron 1, 2, 3, etc) were almost always found in the same position and phase in the aligned sequences of orthologous tetraspanins genes. However, in a few tetraspanins some intron positions are shifted in position by one to three amino acid residues (Figures S5 and S6). This observation raises doubts about whether such introns are really orthologous (we assume that an intron is orthologous in two genes if it occupies the same position in both genes and it has the same phase). For example, the position of the 5^th^ intron in mouse CD81 gene is shifted one amino acid codon relative to that in the same gene in *Danio rerio* (Figure S5). Another example is the *D. rerio* Tsp5/14 intron position 4c which has shifted tree amino acid residues with respect to mouse Tsp14 (Supplemental Figure 6). A closer look showed that the shifts were due to indels at the exon DNA sequence level, flanking the intron (Figures. 5B and 6B and C).

## Discussion

### General Pattern of Intron Gain and Loss in Tetraspanins

Our phylogenomic approach using tetraspanins allows us to specifically address the general observation from genome wide informatics studies that intron gain is more prevalent than intron loss [49]–[52]. Our character mapping of intron loss/gain in tetrasapanins (Figure 4) suggests that intron/exon junctions 1 and 4 existed in the common ancestor of all eukaryotes, while the intron/exon junction combination of 1 through 6 existed in the common ancestor of animals. How can we best explain this distribution with respect to intron gain and loss? Hypothesizing that all six of these “core” introns existed in the common ancestor of eukaryotes requires the parallel loss of introns 2, 3, 5 and 6 in the protist, plant and fungal lineages (Figure 4). An alternative hypothesis is that the common ancestor of all eukaryotes had introns 1 and 4 and that the common ancestor of animals acquired introns 2, 3, 5 and 6. This alternative hypothesis is better because it requires many fewer evolutionary loss and gain events (4 versus 12). Even if the loss or gain of introns 2, 3, 4 and 5 is considered a single evolutionary event, the scenario with fewer steps (1 step versus 3 steps) suggests that the common ancestor of animals acquired the four “core” introns 2, 3, 5 and 6 (Figure 4). This analysis supports a mixture of introns late and introns early in the evolution of tetraspanins (depending on what one considers “early” and “late”) and is similar to the conclusions made by Rogozin et al. [8]. Figure 6 clearly demonstrates that the bulk of tetraspanin introns are the result of gains both at the tips of the tree for the organisms involved in this study, and for internal nodes. One exception to this general trend is the internal node defining protostomes (in the present study protostomes are represented by *C. elegans* and *D. melanogaster*).

### Intron gain/loss is correlated with cladogenetic events in the tree of life

Early studies of intron gain/loss indicated that intron gain is prevalent in eukaryotic genomes [49]–[51]. More detailed examination of this problem revealed a more specific pattern. Babenko et al. [52] analyzed the evolution of exon/intron structure of paralogous genes in several eukaryotic lineages and concluded that intron loss dominates at short evolutionary distances, whereas bursts of intron insertion might accompany major evolutionary transitions. In addition, Carmel et al., [53], [54] showed that evolutionarily conserved genes and gene families accumulate introns more readily than rapidly evolving genes. We can directly test this hypothesis using the patterns observed in tetraspanins. The gain/loss of tetraspanin introns coincides with the major organismal radiations and there are very few intron gain/loss events found in single taxa in our study (except the protosomes, *D. melanogaster* and *C. elegans*). Most tetraspanins (20 of 33 human tetraspanins) originated in the common ancestor of vertebrates [18] (Fig. 4). A few other tetraspanin groups can be traced to the common ancestor of mammals (4 out of 33 human tetraspanins; 18). The appearance of these new tetraspanins coincides in nearly all cases with the concomitant acquisition of new introns. On the other hand, the large number of protostome tetraspanins (from flies and worms) have few orthologs with the 33 tetraspanins in *Homo sapiens*, and hence few orthologous intron acquisitions. Because we only used a limited taxonomic sampling for our comparisons, the suggestion that major intron loss and gains coincide with the divergence of major organismal groups needs to be tested with the acquisition of more whole genomes. Nevertheless, our data are consistent with the notion that intron structure changes are major evolutionary events that coincide with adaptive or anatomical divergence.

### Are intron gain/loss events correlated with function?

The generation of functional diversity in gene families is another interesting aspect that might be correlated with intron gain/loss events. In order to examine this possibility, we utilize the interesting result that three combinations of the nine ancient intron positions divide all the animal tetraspanins into three groups CD63L, TSPAN15L and TSPAN13L (Fig. 1C). All animal tetraspanins are included in these three groups except for RDS/ROM which has only one of the nine ancient introns (intron 6) and TSPAN10 (oculospanin) which has no introns (Fig. 2).

Each of these three groups have a distinct scaffold of disulfide bonds in the LEL variable region suggesting that each group could perform different functions as these particular structures are in is the region of tetraspanins that is involved in protein-protein recognition [23], [37]. The presence of group specific introns (introns 4, 4a, 4b, 4c, 5) in the DNA that codes for the variable domain, could be an evolutionary remnant of the different gene assemblages in the ancestral tetraspanins that duplicated to produce CD63L, TSPAN15L and TSPAN13L tetraspanin gene structures. On the other hand, different patterns of alternative splicing patterns involving these group specific intron junctions could be behind the conservation of those intron junctions throughout tetraspanin evolution in the animal kingdom. Splice variants generated with some of those specific intron junctures in CD82 and TSPAN 32 from group CD63L have been reported in normal and tumor tissues [55], [56] and from TSPAN17, TSPAN 31 from groups TSPAN15L and TSPAN13L (mRNA variants hApr07 and jApr07 from NCBI AceView) respectively.

Utilizing a phylogenomic analysis of tetraspanin protein sequences, we previously suggested that tetraspanins can be divided into four major groups - the CD family, the CD63 family, the uroplakin family and the RDS family [18]. When we combine this earlier classification approach with the intron classification scheme presented in this paper we observe that: the CD63L group include all the tetraspanins of the CD family (TSPAN11, 9, 4, 2, 8, 1, 16, 18, CD151, 53, 9, 81, 82, 37) uroplakin family, (and uroplakins and TSPN12, 32), and the CD63 family (CD63, TSPAN3, 6 and 7). This observation indicates that CD and uroplakin families most likely diverged from a CD63-like ancestor. On the other hand, TSPAN13 and 31 which form the TSPAN13L group are also included in the CD63 family of tetraspanins based in sequence analysis [18]. This placement of the TSPAN13L tetraspanins, which are the smallest group of tetraspanins, in the CD63 family despite their different intron structure and cysteine pattern relative to other CD63 tetraspanins suggests that the TSPAN13L may have distinct functional properties relative to their closely related CD63 proteins.

Finally, the TSP15L group includes all the RDS family tetraspanins (TSPAN15, 14, 5, 17 and 33) except for the RDS/ROM and TSPAN10 (oculospanin). These 8 cysteine pattern proteins are close to the base of the tree in Garcia-Espana et al [18], which is occupied by the plant, fungi and protist tetraspanins, indicating a basal origin for the RDS/ROM family of proteins. Despite being implicated in important cellular activities, few tetraspanins have been studied in detail, probably due to the difficulties imposed by their functional redundancy and subtle functions [17], [20]. The available data does not currently allow for a unifying view of their functions [23]. We suggest that classifying tetraspanins, based on their primary sequences in conjunction with their gene structure, could uncover functional differences and be useful in the task of elucidating their functional roles.

### Are intron loss/gains localized to specific regions of tetraspanins?

Interestingly, 36.7% of the derived newly gained 79 tetraspanin introns are found in the SEL which make up only 10.5% of the total sequence length. A correlation of some intron positions in the DNA with protein structural features has been observed, but how or why this correlation occurs is not fully understood. It has been reported that a non-random tendency exists for introns to be located in interdomain regions of proteins [57]–[58]. There is also a propensity for introns to avoid secondary structure elements such as α-helices and β-strands [59], and in some proteins there is a coincidence of introns with variable surface loops in the protein structure [60]. One explanation is that some of these intron location constraints could be caused by the amino acid composition of these regions which have nucleotide compositions found at different frequencies in various protein secondary structure elements [61]–[62].

The regions in tetraspanins where introns are less frequently found (mainly the transmembrane domains) have distinct and conserved secondary structures mainly formed by α-helices. These α-helices shape the transmembrane domains and form a tree trunk like structure in the LEL constant domain from which the LEL variable domain extends [39], [43]. On the other hand, the SEL loop and the LEL variable domain have a lower tendency for α-helix formation that could explain in part its higher frequency of intron gains in comparison to regions with α-helices. One reason why derived introns in tertraspanins are less frequently found in the DNA coding for α-helices could be the caused by the effects of purifying natural selection, as a result of the chance of disrupting the α-helices. Large alternative splice events or subtle changes produced by NAGNAG tandem acceptors that could be produced by the insertion of new introns would severely disrupt the α-helical structure of these regions. It is reasonable to assume that natural selection will eliminate variation caused by novel intron insertions in α-helices because of the disruption of the primary structure in these regions of tetraspanins. Such purifying selection would be absent or weak in DNA regions coding for flexible or loosely packed parts of a fold and hence such regions could accrue more novel intron insertions. Our observations on tetraspanin introns suggest that a systematic analysis of intron evolution, in suitable protein families where new and ancient introns could be differentiated, may shed light in the mechanisms of intron gain and loss and their relationship to protein structure and function.

### Intron sliding in tetraspanins

That specific intron junctions sometimes differ among species by only a few nucleotides is a well known although rare phenomena [63], [64]. Since the rate of sequence divergence in introns is very high, it is difficult to infer the source/mechanism of such spatial differences between distantly related species [65]. We observed several instances of putative intron sliding in tetraspanins in which intron positions were shifted among different species. It has been pointed out that exon length variations could be caused by extension or contraction of exons at the intron junctions, as for example by the assimilation of adjacent intron sequences by one exon [60], [66]. This hypothesis plus that the shifted intron positions were located in the extracellular variable domain let us to check if intron exon sequences could be implicated as a source of sequence variability by producing expansion or contraction of exons length. To study this phenomenon further, we gathered orthologous tetraspanins in the close relatives to the species in which the shift took place. In two instances, CD81 intron 5 and TSPAN14 intron 4c (Figures S5 and S6), we were able to compare the intron sequence in two closely related species which ruled out any implication of the intron sequence in the exon change of length. It seems the variation of length at the end of the exons was due to indel loss or gain, a frequent cause of divergence [67].

### Testing hypotheses about intron evolution with tetraspanins

The tetraspanin family is particularly well suited to addressing questions about protein function and evolution in a phylogenomic context. Because the structure and function of many of the members of the gene family are well known, these attributes of tretraspanins can be examined for correlation with intron change and organismal evolution. Because the number of introns in the tetraspanin family is reasonably large, and the phylogenetic distribution of genomes that can be used for such studies covers all of eukaryotes, specific evolutionary questions about introns can be addressed. Using tetraspanins, we were able to examine several aspects of intron evolution in a very precise manner. First, we examined the overall phylogenetic distribution of intron change and found that much of the intron gain/loss occurs in major phylogenetic branching events, specifically in the origin of the vertebrates. Second, we show that a proportionately larger number of intron gain/loss events occur in the large and small extracellular loops (LEL and SEL) than anywhere else in the protein structure. Finally, we examine the dynamics of intron gain/loss in the context of reading phase shifts. Close examination of tetraspanins allows for very precise statements to be made about these phenomena related to intron evolution.

## Materials and Methods

### Data mining and Sequence analysis

Intron-exon borders (Table S1) were determined or verified aligning the tetraspanin sequences to their respective genomic region with the Align two sequences option of the NCBI BLAST program (www.ncbi.nlm.nih.gov) and manual supervision of the splice consensus signals. Information about the position of introns was gathered from different sources such as: Ensmbl, (www.ensembl.org).

### Amplification of genomic DNA

Genomic DNA from the bird *Taeniopygia guttata* was prepared with the RNeasy Midi Kit (QIAGEN Science, Maryland, U.S.A) and DNA from the sponge *Oscarella carmela* was kindly donated by Scott A. Nichols (University of California, Berkeley). The genomic fragments of the *Oscarella* and *Taeniopygia* tetraspanin genes were amplified by PCR using the AccuPrime Taq DNA polymerase System (Invitrogene, California, U.S.A).

### Intron positions

An intron was considered to be shared if it was found in the same aminoacid position and the same codon position (the same intron phase) in sequences the alignments of the FASTA-formatted files containing the protein. We used introns positions in the tetraspanins reading frame between intron 1 and 6 and exclude the divergent ends of tetraspanin proteins. To compare intron positions we used several alignments with Clustal W (www.ebi.ac.uk/) or at NPS Web server (http://npsa-pbil.ibcp.fr). Tetraspanin splicing transcripts were searched in the NCBI AceView database (http://www.ncbi.nlm.nih.gov/IEB/Research/Acembly/index.html) and published data [55], [56].

### Establishment of intron/exon structure

Our approach to identifying patterns of intron evolution is to determine the intron/exon arrangements from a wide variety of organisms and to map these onto a phylogenetic tree. By reconstructing ancestors in the phylogenetic tree we can decipher the transition of intron/exon structure over evolutionary time. Two kinds of information on intron/exon junction need to be made clear. First, any whole genome that has been characterized for tetraspanin intron/exon pattern can equivocally be used to determine the pattern in common ancestors. The second kind of information on intron/exon presence is found in incompletely sequenced genomes. In these cases, only the presence of an intron/exon junction can be considered as equivocal. The absence of the junction in such cases is not inferable. Consequently, we constructed a presence absence matrix for all of the known introns and exons in tetraspanins from fully sequenced genomes.

### Mapping intron/exon structure on phylogenetic hypothesis

We mapped these presence absence data onto a phylogenetic hypotheses for the animals in the study. We utilize a tree that places placozoa as the basal most animal. We present a reconstruction of intron/exon structure for only wholly sequenced genomes and for tetraspanins from selected other taxa such as a sponge. The reconstructions were made using MacClade [46]. We implemented the ACCTRANS mode of character transformation which results in accelerating the transformation of characters in trees. The reconstructions for each intron was then used to construct Figure 3 which shows where in the phylogeny, taxa either lost or gained introns.

### Estimating frequency of intron loss and gain

A matrix of intron presence/absence was constructed for all introns in the study and then mapped onto the phylogenetic hypothesis. We estimated the number of intron gains and losses using character reconstruction of only unambiguous changes. The number of introns found in all of the tetraspanins was calculated and then divided by the number of intron losses or gains that are lineage specific to the terminal taxa in the tree.

## Supporting Information

Table S1 List of all genes and their accession numbers used in this study.
(0.19 MB DOC)

Figure S1 Intron junction analysis of the CD63L group. Alignment of full length representative CD63 tetraspanin from human (Hs), mouse (Mm), and zebrafish (Dr) with Sea anemone and Trychoplax CD63-like tetraspanins. The presence of an intron is shown within the amino acid sequences by a number, shaded in red, indicating the intron phase (0 is between codons, 1 is between the first and second position of a codon and 2 is between the second and third position). Ancestral introns 1–6 are numbered above the alignment. Cysteines in the variable subdomain in the LEL loop are shaded in yellow.
(0.25 MB TIF)

Figure S2 Intron junction analysis of the TSPAN15L group. Alignment of Deuterostomes (Mm, Dr, Ci, and Sp) and Protostomes (Dm) TSPAN15L proteines with Sea anemone, Trychoplax and Sponge tetraspanins of the TSPAN15L group. Ancestral introns 1–4, and 6 plus introns 4b and 4c characteristics of this group of tetraspanins are numbered above the aligned sequences. Cysteines in the variable LEL loop domain and introns positions are marked yellow as in Figure S1.
(0.34 MB TIF)

Figure S3 Intron junction analysis of TSPAN13L group. Alignment of Deuterostomes (Mm, Dr, Ci, and Sp) and Protostomes (Dm) TSPAN13L proteines with Sea anemone, Trychoplax and Monosiga tetraspanins of the TSPAN15L group. Characteristic introns of this group (1, 2 and 6 plus 4a) are numbered above the aligned sequences. Cysteines in the LEL loop and introns positions are marked as in Suppl. Fig 1.
(0.37 MB TIF)

Figure S4 Evolution of the Intron structure in tetraspanins supports several nodes in animal phylogeny. Intron structures are represented above the tetraspanins from the cluster we called CD group in Garcia-Espana et al [18]. Cysteine numbers in the varaiable LEL domain are shown in boxes above the intron structures. Species are designated by coloured boxes with a legend for the species designation given. (Species abbreviation are: Hs, human; Cf, dog; Mm, mouse; Gg, chicken; Dr, zebrafish; Ci, sea squirt; Sp, sea urchin; Dm, drosophila; Ce; C. elegans.
(0.16 MB TIF)

Figure S5 Junction analysis of the CD81 group. (A) Protein alignment of the full exon protein sequences between introns 4 and 6 of several CD81 tetraspanins. Cysteines are shaded in yellow. (B) Clustal W DNA alignment of the above exons 5 and 6 in blue letters plus the intron sequence between them in black letters of CD81 tetraspanins from Aves, chicken (Gg) and zebra finch (Tg). Other species: Hs, human; Pt, chimpanzee; Cf, dog; Rn, rat; Mm, mouse; Md, opossum; Ac, green anole lizard; Xt and XL, frogs; Dr, zebrafish; Fr and Tn, puffer fish; Ol, medaka; Ga, stickleback. * indicates indentical bases in both sequences.
(0.24 MB TIF)

Figure S6 Junction analysis of theTSPAN14 group. (A) Protein alignment of the exon protein sequences between introns 4b and 6 of several TSPAN14 tetraspanins. Intron/exon junctions are indicated with arrowheads. (B) Clustal W DNA alignment of exons sequences in blue and partial intron sequences in black of the TSPAN14 sequences from fish stickleback and medaka. (C) Alignment of sequences from zebrafish, fugu, medaka and stickleback.Dashes indicate intron sequence not shown. Species are designated as in S5.
(0.24 MB TIF)
