# Supplementary material for: Intron Evolution: Testing Hypotheses of Intron Evolution Using the Phylogenomics of Tetraspanins
Source: PLoS One. 2009 Mar 5;4(3):e4680. doi: 10.1371/journal.pone.0004680 (PMC2650405; doi:10.1371/journal.pone.0004680)
Supplement: Table S1 — List of all genes and their accession numbers used in this study. (0.19 MB DOC) [file pone.0004680.s001.doc]

**Table S1**

**MOUSE**

>MmRDS NP_032964

MALLKVKFDQKKRVKLAQGLWLMNWLSVLAGIVLFSLGLFLKIELRKRSEVMNNSESHFVPNSLIGVGVLSCVFNSLAGKICYDALDPAKYAKWKPWLKPYLAVCIFFNVILFLVALCCFLLRGSLESTLAYGLKNGMKYYRDTDTPGRCFMKKTIDMLQIEFKCCGNNGFRDWFEIQWISNRYLDFSSKEVKDRIKSNVDGRYLVDGVPFSCCNPSSPRPCIQYQLTNNSAHYSYDHQTEELNLWLRGCRAALLNYYSSLMNSMGVVTLLVWLFEVSITAGLRYLHTALESVSNPEDPECESEGWLLEKSVPETWKAFLESFKKLGKSNQVEAEGADAGPAPEAG

>MmROM1 NO_033099

MAPVLPVVLPLQPRIRLAQGIWLLSWLLALVGGLTLLCSGHLLVQLGHLGTFLAPSCSFPALPQTALAAGTVALGTGLGGAGASRASLDAAQYPPWRGVLTPLLAVGTAAGGGLLTLALGLALALPVSLNQGLEEGLEAALAHYKDTEVPGRCQAKRLMDELQLRYHCCGRHGYKDWFGVQWVSNRYLDPSDQDVVDRIQSNVEGLYLIDGVPFSCCNPHSPRPCLQSQLSDPYAHPLFDPRQPNLNLWAQGCHEVLLEHLQGLSGTLGSILAVTLLLQILVLLGLRYLQTALEGLGGVIDGEGEAQGYLFPGGLKDILKTAWLQGGLAHKPAPEEAPPDEEPPKEVLAEA

>MmTsp10 NP_663338

MMKEEECSPLLSQDTAGREHPLTRNSPPTANIPCPAPWENQKGSWGCRCCPGAKRQASGEGQASSLPLSTGSNCVKYLIFLSNFLFSLPSLLALAAGLWGLTVKRSQGIGWGGPVPTDPMLMLVLGGLVVSVVSLSGCLGAFCENSCLLHWYCGAVLFCLALEALAGVLMVTLWKPLQDSLKYTLHAAIIHYWDDPDLHFLLDQVQLGLQCCGAVSYQDWQQNLYFNCSSPGVQACSLPASCCINPQEDGAVVNTQCGFGALGLDQNVAGQVVFLQGCWPALQEWLRGNTGAIGDCAVAVVMIQGTELLLAACLLRALAVHEAAEDIEAGPL

>Mm**Tspan14. NP_666040**

MHYYRYSNAEVSCWYKYLLFSYNIVFWLAGVVFLGVGLWAWSEKGVLSDLTKVTRLHGIDPVVLVLMVGVVMFTLGFAGCVGALRENICLLKFFCGAIVLIFFLELAVAVLAFLFQDWVRDRFREFFESNIKSYRDDIDLQNLIDSLQKANQCCGAYGPEDWDLNVYFNCSGASYSREKCGVPFSCCVPDPAQKVVNTQCGYDVRIQLKSKWDEFIFTKGCIQALEGWLPRNIYIVAGVFIAISLLQIFGIFLARTLISDIEAVKAGHHF

>MmTspan5.Mm NP_062517

MSGKHYKGPEVSCCIKYFIFGFNVIFWFLGITFLGIGLWAWNEKGVLSNISSITDLGGFDPVWLFLVVGGVMFILGFAGCIGALRENTFLLKFFSVFLGIIFFLELTAGVLAFVFKDWIKDQLYFFINNNIRAYRDDIDLQNLIDFTQEYWQCCGAFGADDWNLNIYFNCTDSNASRERCGVPFSCCTKDPAEDVINTQCGYDARQKPEVDQQIVIYTKGCVPQFEKWLQDNLTIVAGIFIGIALLQIFGICLAQNLVSDIEAVRASW

>Mm**Tspan17 NP_083117**

MPGKHQQFQDPEVGCCGKYFLFGFNIVFWVLGALFLAIGLWAWGEKGVLSNISALTDLGGLDPVWLFVVVGGVMSVLGFAGCIGALRENTFLLKFFSVFLGLIFFLELAAGILAFVFKDWIRDQLNLFINNNVKAYRDDLDLQNLIDFAQEYWSCCGARGPNDWNLNIYFNCTDLNPSRERCGVPFSCCVRDPAEDVLNTQCGYDIRLKLELEQQGSIYTKGCVGQFEKWLQDNLIVVAGVLVGIALLQIFGLCLAQNLVSDIKAVKANW

>MmTspan15 NP_932113

MPRGDSEQVRYCARFSYLWLKFSLIIYSTVFWLIGGLVLSVGIYAEAERQKYKTLESAFLAPAIILILLGVVMFIVSFIGVLASLRDNLCLLQSFMYILGICLVMELIGGIVALIFRNQTIDFLNDNIRRGIENYYDDLDFKNIMDFVQKKFKCCGGEDYRDWSKNQYHDCSAPGPLACGVPYTCCIRNTTDVVNTMCDYKTIDKERLNAQNIIHVRGCTNAVLIWFMDNYTIMAGLLLGILLPQFLGVLLTLLYITRVEDIILEHSVTDGLLGPGAKSSTDTAGTGCCLCYPD

>MmCD151 CD151.Mm NP_033972

MGEFNEKKATCGTVCLKYLLFTYNCCFWLAGLAVMAVGIWTLALKSDYISLLASSTYLATAYILVVAGVVVMVTGVLGCCATFKERRNLLRLYFILLLIIFLLEIIAGILAYVYYQQLNTELKENLKDTMVKRYHQSGHEGVSSAVDKLQQEFHCCGSNNSQDWQDSEWIRSGEADSRVVPDSCCKTMVAGCGKRDHASNIYKVEGGCITKLETFIQEHLRVIGAVGIGIACVQVFGMIFTCCLYRSLKLEHY

>MmTsp9 (TsplR.Mm, tetraspan NET-5) NP_780623

MARGCLCCLKYTMFLFNLIFWLCGCGLLGVGIWLSVSQGNFATFSPSFPSLSAANLVIAIGTIVMVTGFLGCLGAIKENKCLLLSFFIVLLIILLAELILIILFFVYMDKVNENAKQDLKEGLLLYNTENNVGLKNAWNIIQAEMRCCGVTDYTDWYPVLGENTVPDRCCMENSQGCGRNSTTPLWRTGCYEKVKLWFDDNKHVLGTVGMCILIMQILGMAFSMTLFQHIHRTGKKYDA

>MmTsp11 Tspan-11.|NP_081019.1

MAHCKTEQDDWLLAHLKYLLFIFNFFFWVGGAAVMAVGIWTLVEKSGYLSILASSTFAASAYILIFVGGLVMTTGFLGFGAIIREQKSCLSTYFCLLLVIFLVELVAGVLAHVYYQRLSDELKWHLNSTLTEHYGQPRAAEITASVDRLQQDFKCCGSNSSADWQHSAYILSQEALGRQVPDSCCKTVVARCGQRAHPSNIYKVEGGCMAKLEQFVADHLLLMGAVGIGVACLQICGMVLTCCLHRRLQQQFY

>MmTsp4 Tspan-4.Mm (T4S7) NP_444312

MARGCLRGIKYLIIQLLNTVKWLGGCGVLGVGIWLAATQGNFATLSSSFPSLSAANLLIVTGTFVMAIGFVGCIGALKENKCLLLTFFVLLLLVFLLEATIAVLFFAYSDKIDSYAQQDLKKGLHLYGTQGNVGLTNAWSIIQTDFRCCGVSNYTDWFEVYNATRVPDSCCLEFSDSCGLHEPGTWWKSPCYETVKAWLQENLLAVGIFGLCTALVQILGLTFAMTMYCQVVKADTYCA

>MmCD53 CD53.Mm NP_031677

MGMSSLKLLKYVLFIFNLLFWVCGCCILGFGIYFLVQNTYGVLFRNLPFLTLGNILVIVGSIIMVVAFLGCMGSIKENKCLLMSFFVLLLIILLAEVTIAILLFVYEQKLNTLVAEGLNDSIQHYHSDNSTMKAWDFIQTQLQCCGVNGSSDWTSGPPSSCPSGADVQGCYNKAKSWFHSNFLYIGIITICVCVIQVLGMSFALTLNCQIDKTSQALGL

>MmCD9 CD9.Mm NP_031683

MPVKGGSKCIKYLLFGFNFIFWLAGIAVLAIGLWLRFDSQTKSIFEQENNHSSFYTGVYILIGAGALMMLVGFLGCCGAVQESQCMLGLFFGFLLVIFAIEIAAAVWGYTHKDEVIKELQEFYKDTYQKLRSKDEPQRETLKAIHMALDCCGIAGPLEQFISDTCPKKQLLESFQVKPCPEAISEVFNNKFHIIGAVGIGIAVVMIFGMIFSMILCCAIRRSREMV

>MmCD81 CD81.Mm NP_598416

MGVEGCTKCIKYLLFVFNFVFWLAGGVILGVALWLRHDPQTTSLLYLELGNKPAPNTFYVGIYILIAVGAVMMFVGFLGCYGAIQESQCLLGTFFTCLVILFACEVAAGIWGFVNKDQIAKDVKQFYDQALQQAVMDDDANNAKAVVKTFHETLNCCGSNALTTLTTTILRNSLCPSGGNILTPLLQQDCHQKIDELFSGKLYLIGIAAIVVAVIMIFEMILSMVLCCGIRNSSVY

>MmTsp2 **Tspan-2.Mm (TSN2) NP_081809**

MGRFRGGLRCIKYLLLGFNLLFWLAGSAVIAFGLWFRFGGTMKDLSSEDKSPEYFYVGLYVLVGAGALMMTVGFFGCCGAMRESQCVLGSFFTCLLVIFAAEVTTGVFAFIGKDVAIRHVQSMYEEAYSDYLKDRARGNGTLITFHSAFQCCGKESSEQVQPTCPKELPGHKNCIDKIETVISAKLQLIGIVGIGIAGLTIFGMIFSMVLCCAIRNSRDVI

>MmTspan33 NP_062767

MARRPGVPAAYGDEFSFVSPLVKYLLFFFNMLFWVISMVMVAVGVYARLMKHAEAALACLAVDPAILLIVVGVLMFLLTFCGCIGSLRENICLLQTFSLCLTIVFLLQLAAGILGFVFSDKARGKVSEIINNAIVHYRDDLDLQNLIDFGQKKFSCCGGISYRDWSQNMYFNCSEDNPSRERCSVPYSCCLPTPNQAVINTMCGQGMQALDYLEASKVIYTNGCIDKLVNWIHSNLFLLGGVALGLAIPQLVGILLSQVLVNQIKDQIKLQLYNQQHRADPWY

>MmTsp8 Tspan-8.Mm NP_666122

MAGVSSCLKYSMFFFNFLFWVCGTLILGLAIWVRVSKDGKEIITSGDSSTNPFIAVNILIAVGSIIMVLGFLGCCGAVKESRCMLLLFFIGLLLILILQVAAGILGAAFKPEYNRILNETLYENAKLLSDNTDEAKDFQKAMIVFQSEFKCCGLENGAADWGNNFVEAKESCQCTGTDCATYQGSSVYPKTCLSLIKDLFEKNIIIVIGIAFGLAVIEILGLVFSMVLYCQIGSK

>MmCD82 CD82.Mm NP_031682

MGAGCVKVTKYFLFLFNLLFFILGAVILGFGVWILADKNSFISVLQTSSSSLQVGAYVFIGVGAITIVMGFLGCIGAVNEVRCLLGLYFVFLLLILIAQVTVGVLFYFNADKLKKEMGNTVMDIIRNYTANATSSREEAWDYVQAQVKCCGWVSHYNWTENEELMGFTKTTYPCSCEKIKEEDNQLIVKKGFCEADNSTVSENNPEDWPVNTEGCMEKAQAWLQENFGILLGVCAGVAVI

ELLGLFLSICLCRYIHSEDYSKVPKY

>MmTsp6b **Tspan-6.Mm (T4S6) NP_062630**

MASPSRRLQTKPVITCLKSVLLIYTFIFWITGVILLAVGIWGKVSLENYFSLLNEKATNVPFVLIGTGTVIILLGTFGCFATCRASAWMLKLYAMFLTLIFLVELVAAIVGFVFRHEIKNSFKSNYENALKEYNSTGDYRSEAVDKIQSTLHCCGVTNYGDWKGTNYYSETGFPKSCCKLEGCYPQRDADKVNEEGCFIKVMTTIESEMGVVAGISFGVACFQLIGIFLAYCLSRAITNN

QYEIV

>MmTsp3 **Tspan-3**.Mm (T4S8) NP_062767

MGQCGITSSKTVLVFLNLIFWGAAGILCYVGAYVFITYDDYDHFFEDVYTLFPAVVIIAVGALLFIIGLIGCCATIRESRCGLATFVFILLLVFVTEVVVVVLGYVYRAKVENEVDRSIQKVYKTYNGTNSDAASRAIDYVQRQLHCCGIHNYSDWENTDWFKETKNQSVPLSCCRETAKSCNGSLANPSDLYAEGCEALVVKKLQEILMHVIWAALAFAAIQLLGMLCACIVLCRRSRDPAYELLITGGTYA

>MmTsp31 NP_080258

MVCGGFACSRNALCALNVVYMLVGFLLIGVAAWGKGLGVVSSIHIIGGVIAVGVFLLLIAVAGLVGAANHHQVLLFFYMIILGLVFIFQFGISCSCLAINRNTQADVINASWSVLSNSTRHELERSFDCCGLFNLTTLRLSCSAVCKTKSSTCQMCGERFLKHSDKALKILGGVGLFFSFTEILGVWLAMRFRNQKDPRANPS

>MmTsp13 Tspan-13.Mm NP_079635.

MVCGGFSCSKNCLCALNLLYTLVSLLLIGIAAWGIGFGLISSLRVVGVVIAVGIFLFLIALVGLIGAVKHHQVLLFFYMIILLLVFIVQFSVSCACLALNREQQGQLLEVGWNNTASARNDIQRNLNCCGFRSYNPNDTCPASCAKSTQKCSSCAPIIGEYAGEVLRFVGGIGLFFSFTEILGVWLTYRYRNQKDPRANPSAFL

>MmTsp12 Tspan-12.Mm NP_766595 tetraspanin 12 [Mus musculus]

MAREDSVKCLRCLLYALNLLFWLMSISVLAVSAWMRDYLNNVLTLTAETRVEEAVILTYFPVVHPVMIAVCCFLIIVGMLGYCGTVKRNLLLLAWYFGTLLVIFCVELACGVWTYEQEVMVPVQWSDMVTLKARMTNYGLPRYRWLTHAWNYFQREFKCCGVVYFTDWLEMTEMDWPPDSCCVREFPGCSKQAHQEDLSDLYQEGCGKKMYSFLRGTKQLQVLRFLGISIGVTQILAMILTITLLWALYYDRREPGTDQMLSLKNDTSQHLSCHSVELLKPSLSRIFEHTSMANS

>MmCD63 CD63.Mm NP_001036045

MAVEGGMKCVKFLLYVLLLAFCACAVGLIAIGVAVQVVLKQAITHETTAGSLLPVVIIAVGAFLFLVAFVGCCGACKENYCLMITFAIFLSLIMLVEVAVAIAGYVFRDQVKSEFNKSFQQQMQNYLKDNKTATILDKLQKENNCCGASNYTDWENIPGMAKDRVPDSCCINITVGCGNDFKESTIHTQGCVETIAIWLRKNILLVAAAALGIAFVEVLGIIFSCCLVKSIRSGYEVM

>MmCD37 CD37.Mm NP_031671

MSAQESCLSLIKYFLFVFNLFFFVLGGLIFCFGTWILIDKTSFVSFVGLSFVPLQTWSKVLAVSGVLTMALALLGCVGALKELRCLLGLYFGMLLLLFATQITLGILISTQRVRLERRVQELVLRTIQSYRTNPDETAAEESWDYAQFQLRCCGWQSPRDWNKAQMLKANESEEPFVPCSCYNSTATNDSTVFDKLFFSQLSRLGPRAKLRQTADICALPAKAHIYREGCAQSLQKWLHNNIISIVGICLGVGLLELGFMTLSIFLCRNLDHVYDRLARYR

>MmTsp1 Tspan-1.Mm (Tsp1) NP_598442

MQCFKFIKVMMFLFNLLIFLCGAALLAVGIWVSVDGTSFLKVFGSLSSSAMQFVNVGYFLIAAGAVLFILGFLGCYGAHSENKCVLMMFFSILLIIFIAEIAGAVVALVYTTLAEQFLTLLVVPAIEKDYGYQTDFTQVWNTTMEELHCCGFNNYTDFNASRFVKENKVFPPPCCANPGNHTVEPCTEEKAKSMKVQGCFKEILHRIRANAVTVGGVAVGVAALELAAMVVSMYLYCNLK

>MmTsp18 **Tspan-18.Mm (Tspupk1l) NP_899003**

MEGDCLSCMKYLMFVFNFFVFLGGACLLGVGIWVLVDPTGFREIVATNPLLTTGAYIVLAMGGLLFLLGFLGCCGAVRENRCLLLFFFLFILIIFLVELSAAILAFIFREHLTREFFTKELTKHYQGDNDTDVFSATWNSVMITFGCCGVNGPEDFKLASVFRLLTLDTEEVPKACCRREPQTRDGVVLSREECQLGRNPFINKQGCYTVILNTFETYVYLAGAFAIGVLAIELFLMVFAMCLFRGIQ

>MmUPK1a upk1a.Mm NP_081091

MASAATEGEKGSPVVVGLLVVGNIIILLSGLALFAETVWVTADQYRVYPLMGVSGKDDVFAGAWIAIFCGFSFFVVASFGVGAALCRRRYMILTYLLLMLIVYIFECASCITSYTHRDYMVSNPSLITKQMLTYYSADTDQGQELTRLWDRIMIEQECCGTSGPMDWVNYTSAFRAATPEVVFPWPPLCCRRTGNFIPINEDGCRVGHMDYLFTKGCFEHIGHAIDSYTWGISWFGFAILMWTLPVMLIAMYFYTTL

>MmUPK1b upk1b.Mm NP_849255

MAKDDSTVRCFQGLLIFGHVIVGMCGIALTAECIFFVSDQHSLYPLLEATNNDDIFGAAWIGMFVGICLFCLSVLAIVGIMKSNRKILLAYFIMMFIVYGFEVASCITAATQRDFFTTNLFLKQMLMRYQNNSPPTNDDEWKNNGVTKTWDRLMLQDHCCGVNGPSDWQKYTSAFRVENNDADYPWPRQCCVMDKLKEPLNLDACKLGVPGYYHSQGCYELISGPMDRHAWGVAWFGFAILCWTFWVLLGTMFYWSRIEY

>MmTsp6a tsp32 Tsp61 NP_064682

MGHWNRIKIAKCQILITNFLVLLLGLSMATMVVVIHFGDHFTVIGHASLERNPYETLRYWAFYVGISLAGLLSLGAALSTIATVREAHGLMAAGFLCFALSFCILVQVAFWRFYNPTQVEDAVLDTYDFVYDQAMKSPSSNWWQELAVIQDTFLCCGKKSPFGLLVSTGAIMCQGREAMREDCLQSIRNVLWTHYSIASILTCTSLALTVYAMMLCAFLWFAIHSYHGLDRKGRYSLTPPRSHGFQTQEPSLFRWT

**XENOPUS**

>XtTsp2 NP_001106532.1 ESTs this study but this number correct sequence

MGRLSGALRCVKYLLLTFNLIFLLAGSAVTGIGLWLRFGGDLRDAILEDEDEDFRSSFFMGLYVLIGAGALMMLIGFFGCCGAARESQCLLGAFFACLLVIFAAEVTAGAVAFLGKAETVKNLKSTYKEAYKAYQENGAKNSTLSGLHKMAKCCGTEDPDSIKILKPLCSEKQQEYKNCYVEIEKTLNYYFHIVGILGIATAGITIFGMMFSMVLCCAIRNSRDML

>XtTsp4 AAI67361 from ESTs

MTRGCLLCIKITMFIFNLIFWLGGCGILGVGVWLAVTQGKFATLSVSFPSLSAASLFMVTGSIIMVVGFIGCLGAVTEHRCLLLTFFVILLIIFLLEMISMALFLTYTEQFHNYAQEDLKKGLQLYNTTGNLGLTNAWDIVQTEFRCCGVKNATDWLESKGSVPHTCCVEHSPACKSNPKLWWEEACYNKVRNWVESNIRSVGIFGICILVVQVFGLIFSMLMYCQVVKAEKYYE

>XtTs5 NP_001072198.1

MSGKHYKGPEVSCCIKYFIFGFNVIFWLLGITFLGVGLWAWSEKGVLSNISSITDLGGFDPVWLFLVVGGVMFILGFAGCIGALRENTFLLKFFSVFLGIIFFLELTAGVLAFVFKDWIKDQLQFFINNNIRAYRDDIDLQNLIDFTQEYWQCCGAFGADDWNLNIYFNCTDANASRERCGVPFSCCTKDPAEDVINTQCGYDVRQKPELDQQETIHTKGCVPQFEKWLQDNLTIVAGVFIGIALLQIFGICLAQNLVSDIEAVRASW

>XtTsp14 NP_001007871

MHYYRYSTAEVSCCYKYLLFSYNIIFWLSGMVLLGVGLWAWSEKGILSDITKVTRLHGFDPVWLVLVVGVIMFTLGFAGCVGALRENICLLKFFCGAIVLIFFMELAVAVLAFLFQDWVKDRAKDFFENNIKSYRDDIDLQNLIDSLQKANQCCGAVSPDDWDLNEYFNCSVENPSRERCGVPFSCCVPDPAQTVVNTQCGYDARRKKPNERPNTTFSKGCISALEAWLPRNIYIVAGVFLVISILQIFGIYLARTLMSDIEAVKAGYRF

>XtTsp16 NP_001072439 imilar Tsp1 not in mammals

MGCFSFLKTMMFVFNGIIFLGGVAVLGIGVWVKVDGGSFLQILGSAAPQLMHVVNVGYLCIAVGGFLILMGFLGCCGAVKESRCMLMLFFIIILIIFIAEVAGAVVVLAFSSVSRIFIEYLGNVAVKYLHYQYGESDELTTIWNATMKELKCCGFYSYEDFTNSTYYQRNQQYPPVCCANISPCQKYKIDPDVKGCLAAFEHFFSRNGKIVGGVALGICALELAAMIVSLVLFCHIGNSA

>XtTsp1 NP_001016107

MGCFTFIKVMMILFNVFIFLGGGTLLGVGIWVSVDSNSFLKIFGTVSASAALQFVNVGYFLIAIGSLLVILGFLGCCGAQRESKCLLLTFFSIILIIFIAEVAGAVVALVYSSLAETILGPLLKPVLQNEYGSNPDVTKIWNSTMENLHCCGFNNYTDFSNSTFYNNNHQQYPSYCCNSTSSANSVCTQQGAMNSHVSGCFSQLIYLIRQNAAIVGGVAAGICALELAAMVVSMYLYCHLDKEIH

>XtTsp18 NP_001093673

MEGDNLSCIKYLMFIFNFFIFLGGATLLGLGVWVFVDPTGFREIIATTPLLFMGAYLVLAMGGMLFLLGFLGCCGAIRENKCLLVFFFMFILVIFLAELSAAILAFLFRENLSKDFFAREVKKHYHGDNSTEVFSSTWNSIMITFGCCGVNGPEDFNDAHRFRAMHPFAPVPEACCRREVQSRAGKIVSRAECLTGGENYQNRQGCYSVIVNSVEPYVYIAGALAIGVLAIELLSMVFAMCLFRGIQ

>XtTsp8 NP_001011430

MAGVSKCLKYSMFAFNFLFWLCGCVILGVSIWVRVSKSVQQELNIDGGSLLSAVDLMIAVGAIIMVLGFFGCCGAIRESRCLLLLFFIGLFLILALQITAGVLGAVYKPKIEAQLNQTFYKLLPLSGQDESFKTSFENIQKESQCCGLVNGYTDWGANIPASCICPTTSRTNNCIYFAGKNYYKETCLGVISDFFKNHLVIIIGIAFGLAAVEIFGLVFSMILYCQIGKK

>XtTsp15 NP_001039054

MDASEVRYCSRFNYLCLKFALVVYSTVFWLIGAFVLAIGIYAEVERQKYKTLHGIFLAPSIILILLGVLMFVVSFIGVLASLRDNVCLLKVFMYTLVVCLILELVGGIIALIFRHQTMDLLNHNIGKGIRNYYDDLDFKNIMDFTQKQFRCCGGKDYLDWKKNEYHECSAPGPLACGVPYTCCIRNKTDVMNTMCGYKAMNYERLDAMKFIYVRGCTDAVIIWFLDNYTIMAGVLLGILLPQFLGILLCLLYITRIEDIISEWNSSGVLLEGESVKPEIEFSKVGCCSCYPGVESTA

>XtCD53 ENSXETP00000033554

MASNFVNTLKYLMFAFNFLFWVTGCSIIAIGIYFVVNNIYGDLLTNNPSLTVGNALIAIGIIIMVFGFLGCMGAIKENKCLLLTFFILLLLILLAEVIMAILLFVYEKQLDNYVRDRLTSSFEQNLKQNSSETWNIIQRNLQCCGINGTKDWKDNIPNSCCASNANSKCSQADLFKMGCSEALKNWFEKNFLYVGVGTICISVIEVLGMSFALTLYCHISRSSGTLST

>

>XtTsp3 OK NP_001017023

MGQCGLISSKTVLVFLNLIFWAAAGILCYVGAYVFITYDDYDHFFEDVYTLIPGVIIIAAGTLLFIIGLIGCCATIRESRCGLATFVVILLLVFVTEVVVVVLGYIYRAKVEDEVDNTIANVFNQYNGISPDSASRAIDYVQRQLHCCGIDNYLDWENTPWFSEAKNNSVPLSCCRNYVFNCTGSMNKPGDLYSEGCKALVVEKLQEIMMYVIWAALAFAAIQLLGMLCACIVLCRRTRDPAYELLITGGTYA

>XtTsp9 OK NP_001087054

MARGCLCCLKYMMFLFNLIFWLCGCGLLGVGIWLSVSQGNFATFSPSFPSLSAANLVIAIGTIVMVTGFLGCLGAIKENKCLLLSFFIILLIILLAELILLILFFVYMDKVNENAKQDLKDGLLLYNTENNVGLKNAWNIIQAEMHCCGVTDYTDWYPVLGENTVPDRCCMENSQDCGHNSTSLVWKTGCYEKVKMWFDDNKHVLGTIGMCILIIQILGMAFSMTLFQQIHRTGKKYD

>XtTsp7 NP_989350

MASRRMETKPVITCLKTLLIIYSFVFWITGVILLAVGVWGKLTLGTYISLIAENSTNAPYVLIGTGTTIIVFGLFGCFATCRGSPWMLKLYAMFLSLVFLAELIAGISGFVFRHEIKDTFLRTYTEAIQNYNGNDERSQAVDDVQRSLRCCGVQYYTNWTTSLYWHDHGFPSSCCANTSDCNPQDLRNMTINFTKVNQRGCYELVTSFMETNMGIIAGVAFGIAFSQLIGMLLACCLSRFITANQYEMV

>XtCD9 NP_001016989

MPVKGGIKCIKYLLFAFNFIFWLAGTAVLGIGLWLRFDPQTKAMFEADQNTGAFYTGVYILIGAGALMMLVGFLGCCGAIQESECMLGLFFAFLLVIFAVEIAAGIWGFTNKDKVVDELKTFYKDTYTKYMKSNDNALKDTLKAIHLALNCCGMTGVLEATVTDICPNNQGIVGALSAQSCPAAIEDVFTTKFHIVGAVGIGIAVIMIFGMIFSMVLCCAIRNNREMV

>XtCD81 NP_989271

MGVEGCTKCIKYLLFVFNFIFWLAGGVILGVALWLRHDPQTSNLLFQQFEDKHAPGTFYIGVYIIIAVGAVMMFVGFLGCYGAIQESQCLLGTFFACLVILFACEVAAGIWGFVNRDQVSKEMRLFYSEVYQHATTGTKEQQQKALPVLKAFHETLQCCGDASLKKYVTMSITDMCPKRSNILEQITIEDCHQKIDVLFSTKLYLVGIAAVVVAVIMIIEMIFSMVLCCGIRIYSVY

>XtCD82 NP_989191

MATSGCMKVTKYFLFLFNLLFFILGAVILGFGIWILVDKTSFIAVLQTSSSYLRTGSYILIAVGGFTMVMGFLGCLGAVNEIRCLLGLYFSFLLIILIAQVAAGILIYLQRDTLKTEMSSIIHELIVTYDYEDGKNTSAETTWDYIQTNIRCCGWTNYQNWTANTVVQNSTNQYPCSCMKNQSSPNGFCQYNGTVESAVYQTGCMQGVESWLQDNLGIILGVCVGVAVIELLGLILSMCLCRSIQSEDYTKVPKY

>XtCD37 NP_001015801

MAIKGCLSVTKYFLFVFNLLFFILGGVLLCFGLWILFDRGSFATMIGSSVPTLKVWSYVFSGVGILTMLLGFLGCLGSLKEIKCLLGFYFAFLLLLFSAQITIGVLVYTQRNSLSTRLGTIVESVIKDYGLYANQTDLEEGWDFAQEKMQCCGWYTPDDWMKNQKIQNTSVSLYPCSCRNVSAFHTNSSANSSTEFPEAKAGFCTTGAREKWPVYQTGCMNTLQAFLLNNSITIVGVCIGIALLELFVMTLSMCLCRNLDQNYNKLARYS

>XtCD63 NP_001016413

MAVEGGMKCVKFLMFFFNFVFWVCGIALIAIGIYVQIQLNHTLIMKNATSSGAPIVIIVVGVVIFLIAFFGCCGALKENYCMVTTFAVVLVLIFLVEIAAAIAAYVYKDKLRTAFEDSFKNGMVHYNDTKDIADSIDLLQKEFQCCGAINSTDWRQYAPFTGTNTVPDSCCKNITAGCGKGPIANINSEGCATGIDQWVKKNVGIVAGVALGIALFEILGIVFACCLMKGIRSGYEVM

>XtCD151 NP_001116919

MSEYNEKKETCGTICLKYLLFTFNFFFWLAGLAVMAVGIWTLIQKSDYISLLPSNTYAATAYILVIAGAIVMITGILGCCATFKERKSLLKVYFILLLCIFILEVLAGILAYIYYQQLNAELKQSLKQTMTTKYKQPGEEKVTNAVDKLQQEFKCCGSNNSEDWRDSIWINSPEAEKRLVPDSCCKTVTQRCGIRDHPSNIYKTEGGCITKLETFIRAHLLIIGAVGIGIACVQLFGMIFTCCLYRSLKSEPY

>RDS peripherin NP_001079816.

MALMKTKFNLKRRVKLAQGLWLMNWCCVLAGIALFSMGIFLKIELRKRSEIMDNEESHFVPNSLILMGALACALNAFAGKICYDSLDPNKFAKWKPMLKPYLTVCLLFNVFIFFTGVVCFLARGSLDSTLAHGLKNGMRYYKDTDTPGRCFMKKTIDLLQIEFKCCGNKGFRDWFELQWVSNRYLDFSSKEVKDRIKSNVDGKYLIDGVPFSCCNPSSPRPCIQLQVTNNSAHYSYDHQTEELNLWTRGCKEALLTYYTSMMSSMGGMVMLVWILEMVVMIGLRFLHTCLESIANPEDPECESEGWILEKSLKDTIKSSWELVKSMGKLNKVETAGGEGDKEAGVATVS

>XtROM1 underlined seq.from Laevis NP_001079823

MVLFKAKFSFQRRVKLAQTLWLLSWLSVLVGCLTFGMGIFLKVQLWIHNEVMDNTAAHAVPNTVITAGLVGILLGIYAGKISQASMEVTKYQRWKSFMLAFFFFAILSCLVCLAALVLSVALRGTLEESLKIGLRNAIRFYKDTDTPGRCYQKRSMDKLQMDFQCCGNNHPKDWFEVQWISNRYLDFSSKEVKDRIKSNVDGDGRYLMDSVPFSCCNPNSPRPCIQMQITNNSAHYSYNYQSDELNIWVRGCREALLSYYTGIMATNGAAVTLSFLLQASVLVSLRYLQTSMDKISGDDDVEAETEGFLLEKGVMETMNTTLVKIKDLFKSNQVETAEGGGEATAAS

>XtTsp6 NP_001039117

MASPSRRLQTKPVITCLKSVLLIYTFIFWITGVILLTVGVWGKVSLDIYFSLLNENATNVPYVLIGTGAVIILLGTFGCFATCRASTWMLKLYAMFLSIIFLVELIAAIVGLVFRHEIKNSFEQGYLQALKQYNATGDPRSQAVDTIQRTLQCCGVNSYSEWGQTQYYKTHGIPQSCCRVPGNCSEADMKDLGKAGGKVFKQGCISLVTTVMESRMGVVAGISFGIACFQLVGMFLACCLSRYITNNQYEMV

>Xtupk1a. NP_001005444

MAEKGSSGMVACIVFGNIVILLSGLALFAETIWATTDPYKVYPILGVTGKDDVFAGGWIAIFCGFSFFILGVFGILAVQRGSRTMVLTYLVLMMIVYIFECASCITSFTHRDYMINSNVIKGQMLTYYSDSSTPQGRQITSVWLRMMLEKNCCGVDGPLDWVDYYSYFRQSYNETTAPWPLWCCQRDGNFQILNQQGCIVGLSSYVYQQGC AHISNAINSYTWGISWFGFAILMWTMFVMLATMYHYTKM

>XtUpk1b. NP_001037968

MKEDSGVRCYQSIIIFGNVVLGLCGVALTAECIFFVSDQSG IYPLLEATNNADIFAAAWIGIFTGFCFFILSIVGIIGIMKSNRRMLMVYLILMFVVYAFEVASAITAATQENFFIPNLFLKQMLDFYQNPNPTNNDNLWKINGVTNTWNRFMLLNGCCGVNGPQDWQTYTSVFRQSNSDSAYPWPQQCCAMNSLGQPVNLDACKLGVSPYVNLNGCYDQMAGPMTRHAWGVAWFGFSILCWTFWVLLGSMLYWTRIEY

>XtTsp11 ENSXETP00000036322

MAREYKEEKDERGGVFLKYLLFVFNFLFWIAISLTYNVSTVPQSSMVAWGCRLPGLSSGLLGFIDKSLLVVSGGCFASQRVLMHYLSILISVYAAELAAGVLAYLYHETISEELKQNLNKTIVETYAEPGKKHITSAIDHLQQDFHCCGSGSYNDWQHSEYISSTQSEDRVVPDSCCKTKTLHCGRRDHPSNIYRVEGGCITKLEEFIQEHLLLIGAVSIGIACLQLVGVLLTACFLCVLYKEEKEESY

>XtTsp13 NP_989184

MVCGGFTCSKNSLCVLNLLYIMVSLLLIGVAAWGIGFGLISSLRVVGVAVAVGILLFLIALVGLIGAIKHHQVLLFFYMIILFLVFVVQFSVSCASLALNKDQQDQLLEVGWNHTNGAHTDIERSLNCCGFRSYSKNDTCGATCAPDCHTCAPIIESHAAEVLRVVGGIGFFFSFTEILGVWLTYRYRNQKDPRANPSAFL

>XtTsp33 NP_001085454

MARRSPGSGKEEDFSFVSPVVKYLLFFFNTLFWVISMVMVGIGVYARLLKHAEAAMACLAVDPALLLIGVGILMFLITFCGCIGSLRENICLLQTFAICLTLVFLLQLAVGIVGFIFSDKARGKVSEIISNAIEHYRDDLDLQNLIDFGQKEFSCCGGISYKDWSQNMYFNCSSENRSRERCSVPYSCCLHDEDEAVINTLCGQGMQELDYLEAGAFIHTNGCIDRLVNWLHSNLFLLGGVALGLAIPQVT

>XtTsp31 NP_001017068

MVCGGFTCSKNALCALNVVYMLVGLLLIGVAAWGKGFGIVSSIHIIGGVIAIGVFLLLIAIIGLIGAVSHHQVMLFIYMVVLILVFIFQFIVSCSCLAMNRSQQEYFLNTTWTRMSNDTRLNLEKTLECCGFLNTTDAREEFKMDVALCSKAQVCSQNPQKCLSCGDKMLNHADEALKIL

GGVGLFFSFTEILGVWLAFRYRNQKDPRANPSAFL

**ZEBRAFISH**

>DrRDS XP_ 687617

MALMPVKFDLAKRVKLAQGLWLLYWLSVMAGILIFSTGIFLKIELRKRSEMMDNNESHFVPNLLILVGLVACGVNTFGGKVCHDSLDTVKFTKWKTMLKAYMSGCVIFCIVLFVTALLCFLMQISLHFALAEGLKNGMKYYKDTDTPGRCFMKRTLDMTQIEFRCCGNNNYKDWFEIQWISNRYLDFSNDEIKDRVQSNVEGKFLMDSVPFSCCNPGSPRPCIQHHLTNNSAHYSYDHHTEDLNVWTRGCREALVSYYGGMMNSIGAFVLLFIIMQAVVTVGLQYLSTSLETLTDPENPESESEGWLLEKSLKETLSDLMTKIKSPFKGSQVEEGDAEAAPT

>DrROM2 XP_001331586

MVVGKMKFTFQKRVKLAQGLWMLSWLATVGGAITFTLGCFLKTELRRRGEVMDNTDIHCVPNTLMIVGLASMGGNYFASRICQDALDAGRFPRWKTYMKPFFGCSIFFTTLMLISIILSYIMKGSLETSLKIGLKNGIRFYKDTDIPGRCFQKQTIDRLQMEFHCCGNTDYRDWFEVQWISNRYLDFSSKEVKDRIRSNVDGRYLVDGVPFSCCNPSSPRPCIQYKITNDSAHYNYEHETEELNLFIHGCREALVNYFMGLMNTIGAVVLSVFLIQCSVLSSVRLLQTSMEAVAGQENVEIDTEGYLLEKALKETIMEYVDPVMKLLLLNQVGSSEDKAEAGAATS

>DrRD2.1 NP_001104664

MAVLKVTFTKTNRDKLAQVLWVLNWVSVVTGVTLFSMGLFLKVEIEKRRELMSKEIDSVPNMLICVGLTACAINFLGGKICYDCVDTTKFLRWKLLMLPYITCTFFFTFCILVGALMCYSMRKDLEESLYFGLRDAMRYYKDTDMPGRCYIKRTMDMLQMQFQCCGNGGYRDWFHIQWISNRYLNLSNSDVV**E**RIRSNVEGKYLIDGVPFSCCGLYSPRPCIQHQITNNSAHYNYDYLKEDLNLNRRGCLQALLEHYTQIMQSIGLIVLIIWLFEVSVLTGVRYLQTAMENVLRQGDPDCESDGWLLENSIADTARYNFNIIKNLGKCYQVDDDPNIDVPSTSRQMQENVPVKQIPEA

>DrROM1 NP_957308.

MVLLKLKFPFQKRVRLAQGLWLLSWVAMFSGAVTFATGVFLKTELHRRSEVMHSMDIHIVPNLLMAVGLASVGINICAGKVCQDSLDPSRFPRWKTLLLPFFCLSVFFTSLLLVAMILSYALQPSLEESLKIGLKNGIRFYKDTDTPGRCFQKETIDRLQIEFQCCGNTNYRDWFEVQWISNRYLDFTSKEVKDRVRSNVDGRYLLDGVPFSCCNPASPRPCIQYSLLDNNAHYNYEYQSEELNLYNRGCRQALVSYYMGLMNTIGPCVLLVFLLQMAILVGLRYLQTAMEGVLGQENVEIETEGYILEKGVKETLLETKEKMMKLLQFAQVGDASAETPGSEPEAEKPATA

>DrTsp10 XP_001339579

MQSQPLTSYPIQQDVRKSEGASAASYLGTYESSGSHTSSSFSSHEEPSRSSSRRFRFSPASFGTSGWSDYILKYLLISSNLLFTALGLATLVLGLWGLINKESFAQEKISGIGTDPMLLFLFLGLVLALLSMTGCIGALRENMCLLRIFSAAVFLLVVAQVLAAVALFSMQAQMEDYLRLGMLTAIVRYQDDLDLRFITDEIQTSLQCCGADTYRDWEINVYYNCSAPGVQACGVPPSCCIDPLENGTVWNSQCGVGAQQLDEFSAQSVIFLGGCLGSVARWMEHNGGVIVTVVIVLLGIQILTLFITARLLHKIRWTRAQHQAYLQALQ

>DrTsp14.1 = **NP_001006047**

MTGKHYKGQEVSCCIKYFIFGFNIIFWLLGVAFLGIGLWAWSEKGVLSNISSITDLGGFDPVWLFLVVGGVMFILGFAGCIGALRENTFLLKFFSVFLGIIFFLELTAGILAFVFKDWIKDQLNFFINNNIRAYRDDIDLQNLIDFTQEYWECCGAFGADDWNLNIYFNCTDSNPSREKCGVPFSCCTKDPAEDVINTQCGYDVRAKPDAEQKTYIHVKGCVPQFEKWLQDNLTVVAGIFIGIALLQIFGICLAQNLVSDIEAVRAS**C**LFT

> DrTsp14.2 **NP_001006047**

MSGKHFNVHEVGCCIKYFIFGFNIIFWLLGVAFLSVALWAWSEKGVLSNISSITDLGGFDPVWLFLVVGGVMFVLGFAGCIGALRENSFLLKFFSVFLGIIFFLELTAGVLAFVFKDWIKDQLKFFINNNIRAYRDDIDLQNLIDFTQDYWECCGAFGPEDWNLNIYFNCTDTNLSREKCGVPFSCCTKDPAEDVINTQCGYDIRGKGDIEHKTFIHTKGCVPQFEKWLQENLTVVAGIFIGIALLQIFGICLAQNLLSDIEAVRESCLFT

>DrTsp5 NP_001038877

CYKYLMFSYNIIFWLAGSAFIAIGFWAWSEKGVLSDLTQVTRLHGFDPVWGVLVVGTVTFILGFAGCVGALRENICLLKFFSGVIGFIFFLELTAAVLAFVFQGQVREWISEFFLANVKAYRDDIDLQNLIDSLQKLNHCCGAKHPNDWNMNVYFNCSNKNNLSREKCGVPFSCCISEPAVTVLNTQCGYDVREKGTLNDWSSSIYIKGCIDALEDWLSPYLYIVAGIFIVISLLQMVG

>DrTsp15.1  **XP_001337013**

MTGEVRYCEKCSYFFLKFSLIGYATIFWLIGGFILAIGIYAEVERQRYKTLEGVFLAPAIILIVLGIIMFIVSFIGVLASLRDNLCLLKVFLYMLALCLVLELVGGIVALIFKNQTVDILNKNIRKGMVNYYDDLDFKNIMDFVQKTFKCCGGTEYQDWEVNMYHNCSAPGPLACAAPYTCCIVTPGEVVNTMCGYKTLNKDRHENTDVIYIRGCTDAVFIWLIDNYKTMAGLLLGIFLPQFFGVIFTWLYITRVEDAIEEYGYYMDGLLQSDSVQPETKRQSKLAKCCKCMPLMD

>DrTsp15.2 NP_001017802

MPSYEEWRKTIHFYYFLKFSLNVYSMLFSLLGLCVLCIGVYAEVERQKNRTLEGVFLAPAVVLILLGLVMFTVSVIGMVGSLRDNKTLLHMFLCVLCVLLALQAIALIIALIFEKTTIKLFQNSIREGIKHYYDDLDFKNILDYVQEKFSCCGGDEYKDWEVNQYHLCDGKSPLACGVPYTCCIRQSVGEVVNTLCGYKTLHQQREALDGVIHVRGCIHAVNLWMGDNIGATIGICCAVGLPQLLGILLSCVFWNLLVEMSESQDMVDFKFLKRAGYKYSELDLSGAGWCMCLPREEGYLPVPVGEPDTWAPDPIPYYQEQEFTHSQLESKGPHSGMGMDEVDNRA

>DrTsp4.1 NP_001002186

MARGCLCCVKYMMFLFNLLFWLSGCGLLGVGIWLSVSQGSFATFSPSFPSLSAANLVITLGSVVMVTGFLGCLGAIKENKCLLLSFFIVLLIILLAELILLILFFVYTEKVSENAKQDLKDGLRLYNTDNNVGLRNAWNIIQAEWQCCGVTGLSDWHEALQEKSVPDRCCQEHYTECGRNTTNVFWSQGCYEKVEEWLNDNKHLLGTIAMCVLVLQLLGMAFSMTLYQQIHRAGKKYDA

>DrTsp4.2 NP_001002348

MAEGCLRALRYGMVFFNLLFWLCGCGILGVGVWLSITQGNFATLSSSLPSLSAANLLIAAGTVVMVIGCLGCVGAVKENRPLLLSFFILLLLIFLLEILFIILFFSYQDQIDLYAQNDLKKGLQLFGTEGNIGLTNAWSIVQTDFRCCGVTNHTDWFQVYNTSRVPDSCCLEYSDNCGLENPGTWWTAPCYERVKGWLQENLVALWIFALCTALTQILGLVFSMTIFCHTVKAADTYYA

>DrCD53 NP_956665

MSCLKCLKYIMCVVNFIFFICGAAIFGMGIYLMTFSRLSLLPSLQAMSIANTLFITGIIITCVSFLGFLGALKENRCLLISFFILLFILMLAELAAACLMLMYESKIENFIKDDLVDGLNQSIKNRKQHNTTDDWDKVQETFGCCGIQNATDWQGFVPQSCNISGTSNWHKGCFKLLENSFESNLLSTGIGVIVVCIIEVLGMCFSMTLFCHINRSGLGYK

>Dr151.1 NP_001006041

MADAEEKTNSCGTVCLKYLLFVFNLLFWLAGGAVMAVGIWTLLDKSDYISLLSSNTYMVAAFILIGAGAVVVFTGILGCCATIREQRSLLIVFLILLLLIFLLEITAGVLAYVYYQELNAELRADLKERMVENYQQPGQEHITRAIDNLQQDLKCCGSNSSADWRDGAWIRNYADRRLVPDSCCKTPSVGCGVRDHPSNIYKVEGGCISKLEEFILQHLLILGSVGLGIAFVQIVGMIFTCCLYRSLKEEIY

>DrTsp11 XM_687389.1

MGSMYKDEQGDWITVCLKYLLFVFNFLFWMGGGVVMGVGIWTLVDKGEYLSLLASSTFAVSAYILILAGGLVMVTGFLGCCAVIREQRSCLSTYFSCLLLIFLIELVAGVLAYVYYQALSEELKQHLSKTMMENYAQPGKESITQSVDRLQQDFKCCGSNNSLDWMHSVYIMSQAADSRVVPDSCCKTITPQCGRRDHPSNIYKVEGGCITKLEQFLADHLLIIGAVGIGVACLQICGMVFTCCLHRRIKLDPY

>kDr151.2 NP_991213.1|

MGANEEKKEKCGTICLKYLLFTFNFLFWLAGVAVMAVGIWTVIEKSDYISLLSSKIYAVSAYILIMAGVIVMITGVLGCCATFKEQRRLLRVYFVLLLCIFLLEILAGVLAYIYYQQLNDELKENLRETMVQKYNQSEQEHVTKAVDKLQQEFKCCGSNSSSDWVDSAWIRSSEADGRLVPDSCCKSPVRKFCGRRDHPSNIYKVEGGCITKLENFILNHLQIIGAVGVGVASVQIVGMFFTCCLYRSLKSEPY

>DrCD9 NP_997784

MGVEGCPKCIKYSMFLLNSVFWIAGTAVLAVGLWLRFDPKTKSLFEGENSPYVFYT**G**VYILIAAGALMMVVGFFGCCGAIQESPCMLGLFFFFLLVIFAVEVAAGIWGFSNQTKVTEDITTFYRQTYDTYQQSKQEALKKTLRLFQHGLNCCGPSGNMQESLDETCPKKEGLDNLIIKSCPDAIDEVFNSKLHIIGGVGIATGVIMIFGMIFSMMLCCAIRKTREIV

>DrCD9.1 NP_998593

MAAGGGLQCIKYLLFIFNFIFWLAGTGVLAVGLWLRFDAKTKEFFTAENGQTVFLT**G**VYILIVAGAVMMVVGFLGCCGAIKESACMLGLFFMFLLVIFAAEVAAGIWGLSNKDKIVSDVQQFYTQTVKNYKESPDGPLKETLTAIHFSLQCCGPTGLASDGVSVTCPKQEGLANVITTGCSSVIQDMFNSRLHVIGGVGIGIGVIMVFGMIFSMLLCCAIRRTRDIV

>DrCD82.1 NP_997826

MGKGCITATKYFLFLFNFIFFIFGATIMGFGLWILLDNQSLIAVLQESSVILKVVSYILIGVGSFSMLLGFLGCLGAIYEIRCLLGLYFTCLLLILLAQVAVSILIYFQRDLLKTEADKIVSQVVANYPGQNKTAEQAWDYLQRTMQCCGWNGRMDWDENHIIKNNTVPLYPCSCHNYSIQAPIVPDNGFCQASSSDWPIYQTGCLEHVGSWLFTNYGIILGICLGVAVIELLGMIFSMGLCKSVHQEDYTKVPKY

>DrCD82.2 XP_001331673

MGKGCIAATKYFLFLFNLLFFILGAVIMGFGLWVRLDSQSFLTVLQESSTSLKVGAYILIGIGSLSMLMGFLGCIGAIYEIRCLLGLYFTCLLLILIAQVTAAVLIYFQRDLLKGETYNIVNKILVNYTGSNNTSEDAWDYIQRTMKCCGWSGQSDWKENVMIKNNSKILYPCSCRNESISGSDKQEKGFCEGLSPQWPVFTKGCISSLEDWILRNCGVILGVCVGIAVIELLGMILSMCLCKSVQQEDYTKVPKY

>DrCD81.1 NP_001003735

MAVTGCSQCIKYMLFFLNFIFWLAGGVILGVALWLRHDSQTSNLLMLQFEGNQAPGTFYISVYVLIAIGAIMMFVGFLGCYGAIQESQCLLGTFFTCLVILFACEVAAGIWGFINRDTISTELINFYDAAYIKAVDPVDTTSRQTASKVLEVFHDNLDCCGKGDDNDLFKVVQTSLCPKKTFPLDPLISQSCHVKLRNLFSEKLHVIGLAALVIAVIMVFEMIFTMVLCCAIRNAPAY

>DrCD81.2 Dr8 NP_571593

MGVGVEGCTKCIKYMLFFFNFIFWLAGCVILGVSLWLRHDTKTSSLLDLKYEGTESPTTFYISVYILIAVGAVMMFVGFLGCYGAIQESQCLLGTFFACLVLLFACEVAAGIWGFMNKDKISKEVIGFYDSVYDKGATYNTDNKNPATAVLKVFHETLQCCGKGNLFTAIVDRWLTDTCPEHLRTNAVDCHTEIKNLFTDKISLIGIAALVVAVIMIFEMIFSMVLCCGIRNSPVY

>DrTsp18.1 NP_001002734

MEGDCLSCIKYLMFIFNFFIFLGGSFLLGVGIWVLVDPTGFREIVAANSLLFTGVYAILIMGGMLFLLGFLGCCGAIRENKCLLLFFFMLILVIFLAELAVAILAFIFREHLTRDYFTKELKTHYQGTNSTDVFTSTWNAIMTTFNCCGVNSAEDFDDQSLFRRLNPSRIVPEVCCQRTDLMMSKEECLRGIMPIRNKGCYSAVVDYFETYIYMAGALAIVVLTIELFAMVFAMCLFRGIQ

>DrTsp18.2 NP_001002439

MEGDCLSCIKYLMFVFNFLIFLGGSFLLGVGVWVVVDPTGFREIVAANPLLFTGVYIILAMGGMLFLLGFLGCCRAIRENKCLLLFFFMLILIIFLAELAAAILAFIFREHLTREYFTKELKKHYQGYNNTDVFTSTWNAIMNTFDCCGVNSPEDFEESIFRIINPSEMVPEACCRRNNHVGESGFSNREECLSGSMLYRNNKGCYSAVVDYFEMYIYVAGALAIVVLTIELFAMVFAMCLFRGIQ

>DrTsp12 NP_957446

MAREDSVKCLRCLLYALNFLFWLMALCVLGVSAYLRDQLNNVLTLTADTRLEEAAVRTYSPVVHPVVIAVCCFLIIVAMVGYCGTLKCNLLLLSWYFGSLMVIFCVELASGVWTYDEPMVQRSDMISLKSRMPHFGLQRYQWLTHAWNALQTELKCCGVIYFTDWLEMTEMEWPPDSCCSNQYPGCARQAHYNDLSDLYQEGCGPKIYSFIRGTKQLQVLRFLGVSIGVAQILAMTLTVTLLWALYYDHKPPEPASADALIHTHSPTEDPLKVSHSHPRASEAWANTPANGHTQFEMEQL

>DrUPK1a NP_001035332

MGAVTCLMVTVVGLNAIAAAAGLALSAVAIWVAVDGYKLYPISGVSGKDDIFAGAWIAIFTGFAFFLTCIFGIFAALKRSRALMIYLIIMFIIFLFESASAITSATNRDYLVGNSNLVKKQMLQYYADSSTQGQQITMTWNNVMTQVQCCGADGPTDWIQYNSTYRQLFGAASLWPLGCCKRQSSNFEVVDPIGCKAGVTSSMFTQGCFQYIESVLSRYTWAVSWYGFSVLMLVFFTLVIAMIYYTQL

>fDrCD63 NP_955837

MAVEGGAKCVKYLLFFFNFIFWLCGLALIVLGILVHVSLHNTAILQGASGSPMVLIVVGVIIFFISFFGCCGAWKENQCMVVTFAIILSLIVITEIGAGIAGYIFRGKVNELLDQSFNTMIAGYNKTEEYRTTLDSIQKQLKCCGGNSSSDWVNFSADHISVPDSCCKNVTKNCGIGAMTKPTVIYLEGCQPILETRIKENILWIAVGALVIGFVQITGIVLACILSRAIRSGYEVM

>DrTsp13.1 XP_692767

MGCAGFTCSKNSLCALNILYVMVSLLMIGIAAWGKCFGLVSSFQVVGGIIGIGVFLFFVALAGLIGAMKHHQVLLFFYMIILFLVFVVQFAVSSACLAINEKQQNHLLEVGWNNSLTTQRDVEKSLNCCGFSHMDVNGSCAAPCFHYSTCTTCAAKIQEHAGEVLRFVGGIGLFFSFTEILGVWLTYRYRNQKDPRANPSAFL

>DrTsp13.2  **NP_001002748**

MACGGFVCSKTCLCILNLIYVLVSLLLIGVAAWGKWFGLVSSFSVMGAVIAVGLFLFIVAIIGLCGAVKHHQVLLFFYMFILFLVFIVQFSVSCACLAINKEQQNLLLEIGWNKSESMQSDLERSLNCCDFLQVDYSGSCEATCFKEKTCKPCSVIIQAYADDALQFVGGISLFFSFTEILGFWLAYRYRNQKDHRQNPGAFV

>DrTsp31.1 NP_956575.1

MVCGGFTCSKNALCSLNVVYMLVGLLLIVVAAWGKGFGIVSSIHIIGGVIAVGFFLQLIAIVGLIGAVHHHQVMLFFYMVILFVVFLFQFGVSCSCLAMNQGQQEKLLESSWKIMSNDTRISLEKKLDCCGLFNSTNLQADIMSDLHLCTSPCTQKKECVTCGPKMLQYSSEALKILGGVGLFFSFTEILGVWLAMRYRNQKDPRANPSA

FL

>DrTsp3.2  **NP_001002748**

MDCGIITSKTILLLLSLIFWAAGAALAYVGSYVIKSYNNFEDFMSDRHTLIPAAIIIGVAVVMFIIGFVGCCATLRESKVGLGLFLIIIMLIFAAEVTAFVFGIIYRGRIRGDLEKSMNDVFLKYDGLNSETHAVDYLQSQLECCGVKNQTDWTLTSWFAQHNNTVPQSCCKANMTQCTGQLSQPDLLNTQGCEAKLEQVLQDVLSYAMLVILGFAIIKFFGMLSVCVITCKSKKNEYQPLYA

>DrTsp7.1 NP_999939

MSPPSARLQTKPVITCLKTFLISYSLIFWFTGVILLAVGVWGKVNLEFDLLVNSEHGTNAPYVLIGTGAVIIIFGLFGCFATCRGSPWMLKLYAMFLVLVFLAELVAGISGFIFRHEIKAVLKGAYQKAESNYMGKDDEDLDRIQRTLQCCGEENYTSWANTTYFEKEGIPKSCCNVTASVNCTSAELKDLKKADSVVYHQGCFSLMSTTMEANLGIIAGISFGIAFFQVIGIFLACCLSRYITNNQYEMV

**SEA SQUIRT**

>CiTsp15 AK116798.1 (mRna)

MPQEVGCCTKYFLFSFNILFWIIGLCLLGAGIWAWSEKGFFDNLTAISSLPIDPVLVVIIIALVMFLLSFSGCLGSLRENIFLLKCFSICLGVIFFAELIAGILGFVYKDWFHQQFAIFVNKTIKGYRDDPDLQNIIDFSQEYLKCCGGDTGVADWDNNIYFDCASNISFNDVFLKPAESCGVPFSCCIKLGTSNVVNTQCGYGLRDPGVTASEQVTKIYTSGCITQFGDWLKTNLYTVAGVFIGVALIQIVPICFAQNLISDIQAVKAGW

>CiTsp15a No ests;cin130100147944;AABS01000088.1

FFLFQLLSFFLFQLLSLGFIGLGIYSEIWRSEITEAEHFLLTPSVFLFVLGFIGFLFAGFGCVGALRDNLCVLKFFICSVLLCVLIELGAGIAALIFKDPAKNFVNDYVMKCLPTYYDDPDLKNVIDFVQEDFYCCGGRNYHDWEINPYHNCSAPGPAACGVPFSCCKPQPDDDLIVNSMCGYDTLHKDQSPFDVVNIIYVDGCIDAVIDYLWLHMDVFAAILLGVFIPQVSSLCLFKIFI

>CiTsp3 AK114607.1 (mRNA)

CRRYTLMILHCFFLVVGVVLIMLGVFLYSVPGTQSVIQVTTEATTSRQVHQVSLVTMVTGGCVVFISLLGCYGALNNNRCILMTIAVIFTVVLVVECALAAFGIMFQSQIALKVTDQMTISLSLYEGDDATDANSVAWREIQTFFKCCGVNTAEGFHNHTNINVSTSGNYTIGSAWYRNIGMLVNQSWPGSCCELDRVGEVSNPAVCYTDNENNTELHKQGCLDQVVQFLQRHLLTITLLAILLFVTQIVAIILSCQLFKNLKSSGFKPV

>CiTsp1 ENSCINP00000012953

MCLYTIAKYLLFAFNLIIWLAGGGTLGVGIWLLVDPSIQDSMDLAGLEIYQAGAIVFVVAGSLILIIGFFGCCGAIKESTCLLGTYFGFLFVIFGLQLGIGIWALVSYDSMETAINDAMKVKEGGLNQNDDANYVGVEQNLQCCGATRGCKDWATTSASYGCGCDPTSSNLKNISNCVLPNSKDCPDDTFPDTQTGHIYGQPCSAAIYDLIYDNLTIVGAIGLAVAGAEILGMIISMCLCCSIKKKNDHGV

>CiTsp7 ENSCINP00000012956

LYTIAKYLLFAFNFVFWLAGAGTLGVGIWLLVDPTIQDSIDLAGLQIYEAGAIVFVAAGSLILIIGFLGCCGAIKESTCLLGTYFGFLFVIFGLQLGIGIWALVSYDSMQAAINDAMAVPLNSKLNRNDDANYIGVEQNLQCCGATRGCKDWETASASYGCGCDPTSSDLKNISNCVPAHTLNCNQDQNKKTSTTNYIYVQPCSTAIYDLIYDNLTIVGAIGLAVAGTEILGMIISLCLCCSIRKKEDRQL

>CiTsp2 ENSCINP00000012935

MGCCSGIIKYLLFLFNVLFWIAGGAVLGIGIYLLVANNVQAVVQVAGIQFYYAGCYVLISVGCVMFVVGFFGCCGAIKENKCLLGTYFTCLLIIFLAQVGIGIWALVSQSSIETEIKKGLNSTLPLDYSKSDAYANTVVAVQQSFKCCGLVTGCTDWIGGNTTGCSCTPVITNSTVCAVPSSGTCINGDTPGGAIYTSDCYDAIIKFINDNIYLIAGIGLGIGLAEIFGMIFALVMCRSEKSGYETY

>CiTsp18 ENSCINP00000003743

MEDCGKKCAKYLLFAFNIFVWIAGASVLGIGIYLRVGDNIHHIVSIADIQIYYSGCYVLIGAGSFMFLLGFLGCCGAMAENKCLLILYSIFLGIIFVLELGIGIWGAVNQTSIENDIQKAFNQTINNGSNPNDSFAQSVISLERQFKCCGLANGCSDWKSSESYGCTCDVNTAANGTCVLASSLKCTNGDTSGGQYIYKEACYQSIVDFVNNNLWLIVGIGFGIGFVELLGIIFAVMVIKLASKNKYGELV

>CiTsp9 ENSCINP00000003717

MESKGLNCCKYLMFLFNLLIFLCGCVLLGLSVWAYVNADSFKKIISSDPVIFNSLIVLIAVGAVLIVAGFFGCLGAIQESKCLLGTFFTIVLVIFIAEIVGAILIYVYYPKAKDLALQSMQNYNTTTKQPWDILQTTFKCCGFTNYSDWGSTIPSTCCSDAASPCSPSMTTFYPLGCEAAIRKYFWIIGGIGIGIIVFEILAMIFACCLFQNIGDYEMA

>CiTsp11 ENSCINP000000021588

MLITFKKLPYNKNKYVTLNSIWKLIVFCCFQICGAVLLAFGIWAYANGNSFRKLVSANLYLSNSMALMMAVGSILIIAGFGCIGSLMENRCMLGTFFTIVLILFVIEVVSIILLFVYWPKAETLALNSIHHYDTDKKIPWDYLQRSMKCCGYTSYTDWGSEIPSSCCFHNQTCTVGSKDLYVKGCRSSIQHYIFIIAIFGVGALLFEVMFISKYNKWTKMIRVFILLSRPIL

>CiTsp14 ENSCINP00000003743

MSDSLTCCKYLMFFFNLLIFLSGAALLGVGIWVAVGADSFKQVVSQDPAIFNAVYIIIAVGALLFLVGFLGCCGAIKENKFMLGAFFVMVLIIFILEIIGGVLAFVYYPKAKQAAIDSMKLYDDNTPEGNTVKAAWDAFHTAFKCCGINSPTDWLGQTVTFVPTSCAGFTQGCESALKGYFWALGGVAIGVLFIELLAMIFACCLYRGVNKHQYA

>CiTsp16 ENSCINP000000025843

MMTGGLKCLKYSMFVFNLLFMLCGAALLGLGIWIVVDGNSFSTIVASNSVILNAVYIIIAVGAALFVIAFLGCCGAIKENRCLLGTFFVIVLIIFLAQIVGGILAFVYYDRVRPAALGTMSKFNENGTDAVTTGWNTLQAVFQCCGFTNYRDWNETTWTPKVNPFPLSCCARNVLTTTGSIKNETACVAEVPGYFYSVGCETKLKAYYWAVGGTALGVLLVELLALIFTCCLYRAADDDK

>CiTsp17 **ENSCINP00000015326**

MAKKNFNRCIKCSLAVLVLVLWVLSGSVLVLSTYVREDLSQLVLVTEDTTIGDFYLSIFFPVIFPVLVTISCFAFVIGCVGCSGAFTESQLLLGWFFFSMLVAFCVELSAGIWRMVNFKLSYNESDIDQLKLNIQRYSRLEANNWFMKAWAKVHKEYECCGVESFQDWMNSEPLMLGSCCPGSLSGDRSSSGGRDACLDFISELDGCGTKLLADLSSPKSLASVPYITAVLGITQVVALILALKLWLSYCFEAKEDPTSSVSEESRTYEMGYVWDTEKQTLVHFNSANKQIIESASPCLNSKK

>CiTsp4 EST cin 40100141511;AABS01000525.1

MARKNSDKRHGNSGGMCDLSCLRGVLIAFNFLFIIGGCGALAIGIWTVISKMKYAALLGSIYYNLITYLLIGAGVLVLITGVLGCMGAVRKNSGMLTCYFALLVTIFLCECVAGILAFVYYQSLHDELVSELKSNLNKNYNQTGQESFSMAVDDMQQDFQCCGVSAYSDWSGSKFITTNQDGLKTPESCCKSPSPGCSVRDHPSNIYRVLGSDSMGCLTRLEQYIKDHLFILAITGTAVACLEILVMIFTCCLRSRIREEEDEPY

>CiTsp5 cin50100145399|ciona4-5 ENSCINP00000010687

MGKRRLNYLVIIFNFLSFIFGAAVLGLGIWAAVQSGGLNNVVNDVMYAGIYILIACGAVVMLLAFFGCYASVAESQPAIVAYCVILLVSVGLEIASCIILFVFYNASRSGLSDSYMTKYGIDTSITTSWDEAQLKGKCCGKEFSSDWDKSFFYKANGTYPLSCCVRDAESNIVDLEKCNNGEHGFIYTEGCAWILKLYYYAIAGTTIPAIMFQLISVAIIVCLYQYIH

>CiTsp12 ENSCINP00000025966

MNCGMKFVKYSLFAFNLVFFIFAMALIGVGAAVEIKYRSLVTVTGSAISAAPILLICIGVFIFFVSFFGCCGAYKENYCMVSTFAILMALIFILEVGATISAYVLRGKIKMYLEKSFTKSIEEYKTSTKGAFNFVQKTFHCCGSTNESDWSRSVVFQQEANQTRIKHNIPLNESASYYVPDSCCKQMIPQCGIKSNRVLNNKGCVATIEASFKKNIVTVGGIGLAVIFIQSLASYSHVC

>CiTsp6 ENSCINP00000015991

MVSRSMKTTPAVTCMKTLLAIFTLIFLVIGIALLVAGTYSKISLTSYNLLSTTDFTSVPYVMIGVGAFIVVVGLAGCCATMKGNTCLLRTYAVCLGVVFLAEVVGALAVIISRNKINNGFEAGMQNAMKKYKTDGAYKTAVDNAQKTLHCCGSNNYTDYYTLPGWNPNTVPMSCCSNLTYCKEHLVDNKVEIVGLPTQDASEYVYNQGCPQLVFGVVNKNLGIVIGSMFGVAFFQLVGLILSCCLASSINANKYELV

>CiTsp8 BW315030;Cin 80100132804

MASSAKSYRILSCLKILLVIFTVLFLCTGVVFLATGIYALMTLSEYNKLTPMVDLSMTPIVLCASGGFIIISGLFGFVSAFKNNKCMVNTYGCLLGVIFLVEIAGGILSIIYINQIRDSFEVGFRQTMQNYYSSNQALMDKAQIDLQCCGVYSYTEWFNTTWRDNTEQCNGIITRARGDNFTDIVNINTQGCVAVMFGSEGQTLYYLISACFGLAALQVIGICLSLCIYQQQRRIRASRRHY

>CiTSP13 from ESTs BW399959; BW475985; BW399953;

MCGGFTCSKNSLGFLNVVFLLVGVLLIAVAAYAKAAAQITSFEICG**G**IIASGLFLFLIALLGLIATTKHHQVLLFFYIVILFLLFIIQFSVSIACLALSEMQVAKVLGNVWGTAPSNTKNDAQAYFKCCGWLENDPTSQDCILAKACQELPTTTQITTNPATNSTPVCTTCSSIIPQTVSKGLEATGGIGLFFSFIEMLGVWLAVRFRNQKDPAANPSQFL

**SEA URCHIN**

>SpTspXp XP_001177545.1

MKVLKCCSIALSAKFIMFCLSVLFWIGSAGLMYLGIEMFTYAGNIHHLASNYFLTIPASVCIGLSILFLTVGIIGLIALTREDARFLRRMFVALLMVIVILEITGAALAIAFKEEINTGIDNGLNNTMKHYNEKENYQRSMDYVQGHLDCCGSHHAEDWATTPWGHANPDMVPLSCCKDNATANCTGSLVNDQFNINHDGCQEKLYDDIKAYLVYIIIIAVLLFLLQSMALCCTCYLVCHRRDKQYQQLQTPSKRESSYGYRA

>SpTsp9b XP_787272.1

MGKKTQPYPQPASSQQQLHYHQPPPARVRRAPHGITVSICVKYTIFAFNVIFWLCGLGILAFGVWGLVSKSVSSVEAIAEEVGIKLDPMYGFIIVGGCIFILAFLGCIGSLRENTCLLKLYVIILVLIFLAEITIGLLVYFYQDRFVTLLDTWVEKTLLNYFDDPDSQFLMDNMQEGLTCCGVNSPDDWQKNAYFNCSSIADSRCSVPFSCCVPDPTTSVINYQCGYGVLALPPTEWFMTIYTIGCAQSLTDWFKTNVILLSCIGGALVLMQSIAICLARSLIGDVKEVKSYW

>SpTsp15 XP_794304.2

MPRIRKPDCGHTTLKVLVVIYNIFFWLVGATILSVGIYAEFEKRNYEAVSDVLLNPSTVLIVLGAFMFILTFIGCVGALRENILLLKIFGWTITIVFMLQLIAAIIAIVFKTKAKELVSSGFSSALENYYDDPDIHFAVDAVQQKLECCGGFDFNDWDKNIYFACQDPGSCGVPFSCCVSTKEDMVMNTQCGVNVREGDPHPFAVDDTIYIRGCTDALLVWFQDHLDIFIGLTLGVCLPQLIGCLLTWLFIGKIREAQDQYDYHQAPTSDKK

>SpTspCG XP_788310.1

MVAPERIPNNVINPCVKYTLYALSFLFWLVCWFVLGIGIWALVERGDNVKVTSYLDFFTDPAIVMVVVGGLGIILNFAGFIGALRENCCLLTFFYLSMILIFILEVTAGILAFAFSGQFFTAVDEVVESAIMNYRQDGDTTNFIDYAQRTLECCGGDGGYRDWTLNRYYNCSDSNPSIERCGVPYSCCRTVSDDDIINTQCGFNVQRSESAEIIDTIYIIGCVDGFVLFVNEKSLIVGLTIFGIALIQLLVMLLAFILSRQIEKEYEFYEEFKNRGLTGISTVSGGNRF

>SpTspDN5 XP_794023.2

MGSKYDKEPCCGISLLKYVLFIFNFFLLLGGAGVLGVGIWTVISKFDYTEVLCSHAYIIATYVLIISGGVVIIVVITGCYGAVQEVKGCLLVYFTLLLLLCLVELAVGVFVYYYSGELQVELEKCMNSSMTKNYGVTGNEAYTVTVDELQTSFNCCGAASFEDWEKSEWKQNGNAGNRSVPASCCKTYSPYCSIRTHPSNIYHQGCVVGLSLIVEDHLIIIGAVSLAIAGAEIMGLIFSMCLYCHLRYEEQEPY

>SpTsp9a XP_786750.2

MCCCSISCVKCFLYFLDVLFLLSGIGLFITGIHVLVGVTSGAYASLLPNISYMYAAYILIIIGIFIVIVAIVGCVAAVREGVCCLLTFYFMVVIVICLEVSAIIIAFAGLHMRGDMSSYVSASMKRGLYYYQDENHAEMKTAWDTLQQEVECCGVNSPTDWEVTGGFPPGIVPNSCCNGPEGACTSHYMEGCEDYILMELQDWLTLIGVV

CIIFVIIQ ILLVWFILILFFKLHRG

>SpTsp4 XP_798173.1

MRGNINCTKYMLIAFNVLFFVVGGAILGAGAHLHIKEGPFMTLLPSMPFLNVANVMIFAGIVILVVSFIGGYGAIKEHQCLLLTYFVFMVLVFILEVTAGGLGFVYRWQVAKYVANEMRAGGLAKYNTPGEGGLTKAWDKLQSTLACCGVFNDTDWANTANGMFLPRQTPDSCCLFAAPGCGKDVNSEKFIRGCKGAIIMELESKAHVVGIACIIVAIFQIFGIGLAYTMWKSVSDRAGGNYV

>SpTspDN7 XP_800749.2

MALEGCAKIVKILMFIFNFIFFVAGIVVLAVGIYVNVVDGDFAQILPSFSYLSAGNLLIACGVIVLVVGFLGCCGAIKESACMLLIFFFLLLLILILEIAAGALAFTYRSQVKGFVIKDLTAGLSQYNKSQSLTKAWDVLQSELKCCGVNGSSDWTTTPNVTLNGARFPDSCCDPAFEKGCAATGTAWMEGCQPKLTMKLEDNIYIGGAIGIAFGLIQILGLVFSM

>SpTsp13 XP_783097.2|

MVCGGFSCSRNALIALNSLYIIVALILIIVPSLAKSGSYVSSLYIIGGIIACGIFLFIVAVAGLIGAIKHHQVCLFFYMLVLFLVFLIQFSVSIAALACGKDQREKLVRLSWEHSSNETKTDIQNGLCCCGFDKNDTSHPTCPTVRNDVKCGDQTCQDKMDEGYAQGLKISGAVGLFFSLTELIGVWLAVKFRNQKDPRANPSAFL

>SpTspEST XP_800780.2|

MVEGTASCIKYLMFIFNFIFFIFGLGIIVGGSLTLTLYSEYVDFTGNTGTAIPIALICIGVFIFLTGFFGCCGAVKENYCMLGTYAFIMVLLLILELAAGISGYVLRDDIDKLVDDNMTDLQKDYNSSTSTQQLFDNMQKNLECCGVDNYTDWMGYNMSHPDMVPESCCMKPGTAGCNQVGSATLDIYTKPCYNALKDLLLRNIGIIAGVAIGIAVIEIFGIAFGCCLMRRSRMNTRQFKMATMGDVWGFILGFLPSLII

**C. ELEGANS**

>CeTsp13 NP_741692

MPHQKYVDDTRRVYSPVPSPSYHNGRSMVSSDMYTYTTDTNVTDSEYANRAGCGVWAKYGIFTANIVFLIVGGLLLAMGVWLRTDSRFRNFISERYRQAVQEAFWEAPTLFAFSYIIIVLGAVMMVVAMLGCCGITGRSRPFLIIYSMVVFLLLVATLSCGIYLLYKKDGLDVELSDALNYMVQHYYQGPGVVQESLDHLQTAFRCCGNAGCSDFRVFRQDIPRSCDIRCDGCHFRIMIALRIGFSVTLIVFSAVVLCQVLTICFALYFVFMREKEVKIIYVEPPHRRHSHRLTRDTLRDHDLPFQLQPKKPRRYASKERY

>CeTsp14 NP_508828

MGSGEPTRARAVVSSSHKQRKPRQEISACLKWLVFLLNSIVFLVGVGILALGVYLFIKDFREVKLVDIILNPAILISIFGFSICVVSFFGFMGALRDNIFLLKCFAACVFLSYILVVAVTLVFFTLFYTDTTEGLSANWLLLYAVKNYHTNRNLAEIMDALQENLECCGVSSIAQGYRDWNMSYQFNCTNSNPQPEKCGVPFSCCRKSVISEAAGSSNPLLPAMRSLECWQNALTKRPGDLEHDIYTRGCLQPLRTLFESHAVHVGAFVALLIVPVCISVCLTNILAKQVDHQRYLLEREARRNDRRRKRDHNRRDQLNSLDLLEEGKFNNASANATRPRPPDIPPPLPPIEHVPRKKSRNASSSPTRKPKSAGVENAAARRKRTATTTRTPPAAAGPAPTPQATTTNRTHQWVLQQTDLVPQKSKS

>CeTsp12 NP_501853

MANRRQPVQHRAQQRVYRQSQIRYAPGAGGESEISCCVKYSVFSFNVIFFLLGFGLLLFGVWAQIEKNTFVNMLSKASKLYLDPTWPLLIVGFLTFIIGFSGCVGSLRENTSFLTFYSTLLGLLLIAEFSAGVFAYACRDQLDNYIRNLLNDVVVGYRDDPDLQLLIDSMQETWMCCGINGADDWDRNTYFSIEAREVASPEAGGVPFSCCINSSKLEFKNYFCGHGVRLKPESHMAAHLAAQRVMAHTASIYTEGCLPKLQLWLNNNMLLVAVSMVIIAIIQVLGICFAQNLKSDILAQRAKWYYTH

>CeTsp17 NP_508634

MLLDPKRNYILDLVHFSENDPLLKASAYVSLVCGCAQLLVGFLGLCGAVNRSRFLLLAFVMFLIGTFLADVAMGTLSLFYKDKVYSEKENVREIVKSKYGVMTSEPENKMVTEFIDKLQFYEKCCGSLGPTDYISSRWSQSTNQDSEEIESPLFPVSCCTQITGASALNPLAKSYARCQQIGANRQWRHAVGCSERLMSWFNEQIWIFVGFGFGSALTMMLGICLSCCLISKIRIYHVIRDDY

>CeTsp8 NP_510445

MGSCVNALRIVTFLFNFAFWLSGVVVFGLGIWLLFDPAASDFFALHSTHPGAFRYVGWFLVGAGAIIILVGYFGCIGAWKMNQCALAFFCCILILAFFLELAAAVTLFHKQEHIKHYVESSMYDTIRNRYSSETAFKDAFDTVQEKFECCGVKTYTDWLSARWDAEPSTQLEVNEEDAGRIEHGIGAFGGNKGTGYGRVPSSCCNEHGKLSYPNNCGRSFSQAPLNTYAQFINTRGCADAVYESVSSSLSLIVGVCVVLCIVQLLGIVLSMTLCCCKGNSKK

>CeTsp9 NP_508232

MVCGNSCIKLLFFVINFFICIFGALICGFSLWANLDKNFGSHLSDFVRQIEGIDQKLVNEIAEYQASLWILVAVGALLFLVGFFGCCGAGCESPVLLGLFIFIIVILTAVELGATVFAMTNREEFVSSIQQVLKKSSATYELRKNIKPIQNVFQCCGATAQTQNRYIQDGLCGPEPLSAPVNCFDRISHMVQSWGESIVVVAFLLLAIELFAILFSCILCRSSQEIRYTPYYS

>CeTsp18 NP_495178

MVVCLMRISAKSRKLIGIFNGALIGFILFLEVLLVILKNDKLIEFYNIGIFQWLRDQDSVYLYSNFIVSFLVLCSVCLLIFVFDVATSQESEAFHVNLKTYSISIYLLCVSFMVCVLSYTLSLRISSEYDTKMTTILMTNYLNYNESMESRRLVDRIHIEYSCCGVNSIEDFVDLSEVDNKLPTQTKLWPCNEWEYCSVPLSCCKTVSCSQKVELLNDGWDQANITNKWFNKIGCVKKMDNAWLFFNFPSYKLNDFIMILVLGFHICSLILTQILVTSSATLHGARLETSESSYAWLIDVGQPDSVALIKKLNPDILPSEFEASTSVGKDEKEIEPEKDVDMNAQTALTTTAETEHTATMNSTTAPVTTTNTAETTDATLVSTSTSNIGKLSTSRDDGVLSSDSNDKLTKKSTEHPMKKEKQTKNKVQLRKSTETKQKPNVVAKPQKKTDTKVNTAKKNPTPVKKAPSKKNKK

>CeTsp11 NP_509546

MNFRNQSTMPLSCTARALKFSLFIFNLVFLLCGLICLGIGLWLVLDKYAIDNLAFATAKDFQATKPTAVRQFGYLLFVGGFIVIVVAFLGCCGAAKEWRPLLCCYSSCLMLILAIQIAATIYAFLHSHMFENDFRDILHSSLKMYNGTDNMKVSNNPQDGLLVKTAWDKIMIEKSCCGVDSKIGEFNNSGWYQLNHGRYQFPPACCPPDEHGRLRPYCNTIMRHSHGCITPLYRESQIHFSAIFSILVVLTIFDIVLFIVGVQLFRHIRIEMLYYF

>CeTsp1 NP_498804

MATWKFIIRSVLFFLDLAMLLAALALIAVGFWMGYDSSFDTDLKNVIYKYDDPKSLADAKFNIRVWLIVVFWSIIGLSLGAVVTAVLGMISSVWPKRKGFMITYLVLIIVLVSLEIGCGVAVLVRRNSLHDNTNSLIDAMYTTNSVNDLKIIQDKYNCCGIENSLFNVMYCGPMSQKPHCDVAVFDSVDNTMMISGIILLVILILQTIAIILPVPILISRKKTYKYSYEPRVTQLADITEDTRF

>CeTsp2 NP_498803

MTSRIAFISRITIFCLSLAMLFCSIGLDIWGSLMEDKSYERDLKDVVFNYNSSQPLADDKFDMRSWIYSVYWSIYGLCIIAGPLSIIGLIGAISQKKPVLATCLVLIVIFFIMELGGSIALWSKRGSLRSLLYRFANDIYLTNSVFDISIIQNTYNCCGVQDGQWKCPNAPSCDIALFNSVDNTMMISGIILIPVLLLQIVIIGAAVLVLVFEPRNVLRKIEAQHENPAWESSE

>CeTsp3 NP_499724

MSFCTFLARIFLFFLNLAQTLVGFTVIALTLWIRFDKGFESEIRTNILRDNDPEPLAGVKSDIRTGIIVSFWIIIGFAIANVIIGFVGVIGAVIRSKYLLAPYFLFMVILFLLEIAIGITVLVKRRSVRRTVKEYVFDSFNMNAQADISAFNFRYNCCGAENLPNLNCFAGQPTCSSAVWDRLDFTMMIFGICMLIIVVLQMFTAFLTVPIIIERKRETSYQQ

>CeTsp7 NP_492636

MVEGGVTIVKYLLFLANLVLWVGGLSLIIVGSILQLKFDNVLDILGDERLATPILLLVIGSLCTLLGFLGCCGAIRENYCLTVSFAVLLALLITCEIAAVIIGYALHDSFRLGIGNQLQTGMVRYHESRGVESAWDKTHQLFECCGVTNSSDWLTFTTIPDSCCIEEIEGCARENAPLFEPGCIHSVEQWVLKNGAMVGGICAVLAAIQLVGVCFACCLSKSILKDFHDFYY

>CeTsp15 NP_492404.

MGALGDSAYGARGRLIKFSYIVTALISILFSISCICYGIWLLARRSQYAELVSPSLYVDVGRILVIISILSILNYLICFYAIFKEMRCFVTSCAVASIVIAVMLIIGGCIGLNFRDQLTHYTPLNLKMLTSLRELYGTHDMKGITESWDALQSNFKCCGVNGTDNAQIWKTSKWYMHQRAPKLLIPESCCIPSEIERCRSNPFDQDAPPPYYTSTCYEPLQNDLLHVMNVASWLCITNAIVQIIPSVAGCWYSKLIRK

>CeTsp-10 NP_001040805

MVELNNQDKVFLGASLATAIIGIALIVIGFLFRFGGGFGTFSTYAQQDNDFLELKRLDMIFGLFVAAACVLVISFLVATVSVLRHNSFLLKAYCAMVALMIVVQLVDGLLAFTYSDQVNQLASDDFMYESLSKAAQKTIPPIGSLSSDAEVQFWANTQDLFGCCGVYNSTDWSVFWGVTGTNNDLQALHCYPQHFNDGCEKTIRNKISSNAQYLGAASMGVLVVEIIASFLAGYRAYTLA

**>**CeT**sp16 NP_509183.**

MRDLTETVSSNSKYFWQCVCLRLCVLLGSVTFQYLFVFPSAHRHIESFEREKEGPRGNESDRIPLTAVRRVKKTYLLINNRHVEQKRGQCLKKCLALLLSVLTMIGSGFIVGFGIQTLREVSFTASVIGTNLIVYCPYLLILVGILSFVMTPVRFFSIILNDNKIMITHMFATIFFATICAMTAILGYDLNSHVSSSDMEHWMKHSIKEDYGNPTAPHIMEEWNKAHRQFKCCGVRNLTDFVESKWYIMQKKHPRQRIPDSCCASCATMHERFCVAFFKEPGNQPHQLVKNQTICLQASNGCLSADSSIANREPLRTTLEFFSFRIFIYSLSFLAFLALSTLIWLLNYQISREALPFQVI

>CeTsp-20 NP_510014.

MRPVHQHRDHPPRNRRSGSCSGCCSKCQLFISYLLVIVGILFLALAIWLYIFRGDLIPLIQSHFYVKCIYLAVGCGVFNVIIGFLVHSAVSNRCALVFYLLMLIFSMIMEGCLIYFTFSYHATYEQELNASLPNDILNNYNLDPNIAKAVNYLQRDSKCCGSNAFNDWPKPEVEDHYLPYAKTVVQRVQYIPDSCCKSTHQRKGCALSDSPNNIFYRGCLPFLKEEVYNNLNFLFTVTAASLVLHFANLIFGCCTCFRSDEDEEENPFKDYDQEMHLFD

**DROSOPHILA**

>DmTsp33B NP_523552

MRQPFRRASVYLHLLLITAVIGLLILVVTAYYHTVLTGYLSDIECRLVYGYLFGIYVFGAQVVVTFLCSIAMWRRIWRRRCTPNIRLLLSVWAFYSCVIIASGFGCVWNLYRGVDVLENAADTSLTRGIDMYYSCPEWKLLWDGLQWHKECCGVHGYKDWMNAEWMPRRENNCTSMVLAPFACCKRSCDSCFNNFLPSEGQSIGGNSRQPFPALTVDSINANGCLPAFVSAVWNCFYILMALWVLALKFLIVLCCMTKFIVHRQNEGDGCDNVGLTDDDGHPLVVVKYPCNVRCVTIAEDDLVSDNVPDINYCNCTEMDDEPCGY

>DmTsp2A NP_525037.1

MGIGYGASDEQLEKQIGCVKYTLFCFNIVAWMISTALFALTVWLRAEPGFNDWLRILEAQSFYIGVYVLIGISIVMMAVSFLGCLSALMENTLALFVFVGTQVFGFIAIVAGSAVLLQFSTINSSLQPLLNVSLRGFVATSEYTYSNYVLTMIQENIGCCGATGPWDYLDLRQPLPSSCRDTVSGNAFFNGCVDELTWFFEGKTGWIVALAMTLGLLNVICAVMSFVLVQAVKKEEEQASNYRR

>DmTsp96F NP_524500

MGLNGCCSCVKYLMVLINILFWLIGLTIVVTSVWMLTDPTFMLSMTQNYNHYHIALYVFLAIGILITLGAFFGCCGVCRESQCLLVSFFCVILIVMVAQIAAGAWAFHNKDKLDDIVRAAVKSSVQEEYGQSTMSSRTVTFDTLQKNLKCCGADGPGDWATSRFNNVDRTNIVEIAVSSMNVFYNIPESCCKDNLKDNECELSRRLKFGGPLNNAIYQQGCVDKLIEIIYENWVTIFAVTAAVILLELLSLTFALSLCCAVRNQHYKA

>DmTsp26A NP_523487

MPAAVRKFRRETSEISCCLKYLLFASNVILWLSALLVLSVGIWAWSEKGMFRNIARLHFIALDPAFVLIILGGVTFLLGFMGSVGALRENTCLLGAYAIFLSVLLIAEIGFCAVAFVLKDKGWIKDQATEGLKAFIRHYREDADQQNLIDWIQEDWLQCCGIDGPKDWDSNNYFNCSSIAIGSREACGVPFSCCRRRPQEVIKNKQCGYDVRKEGYPVDRNIHERGCLRAGEDWLEAHLISVAIGCVALLVLQVQNISIVVVVYFFLNL

>DmTsp3A NP_525054

MSNYRYQGAGLGGVGMAGGRGYSGIEVHEVMHPHHFTYVSQCVKYMIFLLNFVFWLFGGLLLGIGVYAFRDKWEDANGSVRLENFYDVFLNISLVMILAGTVIFLVSFSGCVGALRENTFLLKFYSMCLLLFFLLEMAIAIVCFVCPQYMNTFLEKQFTHKIIHSYRDDPDLQNFIDFAQQEFKCCGLSNSGYQDWSKNEYFNCSSPSVEKCGVPYSCCINATDISSGLVNIMCGYGVQNAPVPEATKLIWTSGCIEIVRVWAEHNLYVIAGNALGIALIQLLVIYLAKTLEGQIELQKSRWLA

>DmTsp86D NP_524309

MSNHRYHQGGNYMHPRISTYPHHFSYVSSCVKYMIFLLNFLFWLFGGLLLAIGVYAFMDKLMDGNGWLRLDTIYDVIFNISLVMIIAGVIVFTVSFAGCLGGQIDLQKSRWSALRENTWLLKLYSMCLLLFFILEMSLAIICFVFPQYMNSFLEYQFTDKIIHSYRDDSDLQNFIDFAQQEFNCCGLSNAGYQDW**S**KNEYFNCSSPSVERCGVPYSCCINATDISSGLVNIMCGYGVQVRSVAAASKRIWTSGCIEIVRVWVERNLYVIAGVALGIALLQLFVIYLAKTLEGQIDLQKSRWS

>DmTsp29Fb NP_723426

MSNRELQLNSGMKCAKYMLIIVSFMFALTAILLIMVGTTIQTIFGDFSLFIDGHFSSPPALLIAIGFILIAVAALGAYGAVKESVMVINLYGVCLFLVFILEVSAAIAAFVMQSQVRGMLIRTMNQALAEYEHDPYVESGVDFMQSMLECCGVNEPEDWKDYLSANVNFTLGVDDVVVPNSCCGNQPTSLNDSTQMTCMETYDYGCFRKMNFIVSQSAMLIATGATTVAFVQLLGVLCAFMLAKTLRRNKSIREARRWQLQQSLGVLISGGKMAPPQNSAVTGYQQLDNGEQGSHEPYTYTPQSPSVN

>DmTsp74F NP_730317.1

MGFSSRMDCCGQFVKYSLFIANFVIFVGGAIVFCLTLWTLVDRSFVNELLGTNLFSGAVYVLLVTSIIICLVSFLGCVGAGKEVKCLLLTYFIIVALVFVTMLIGGVLGYVFRERVQQTMRQEMRSTMALYGSRREITQAWDLTQERLQCCGVDTWHDWNRYGPVPESCCQELFGGQRKECTIFPTITNLYNQGCLYVTTNFIRDHAAVIGGTSIAVAILMIFGMIFSCLLFNMIE

>DmTsp42A NP_523623

MANPFRWCRVSHDCVFQINVVLVVVGVIFLLDVLSHLYLKAVMFPGLRLYPVALRPWLFWVRALTMVAYILNAVLGIHMARQPTVLKYAGYMLVGSVVLLYTISIGVTRFMYRKRFEFFAEMLVLQMWVRDRLGKVEVEFECCGRSSVVDYQTASSNRTWPIGSCCGKQNCTGCTAKLSQYLWTIEMDVARDNIIVSVLLFVAMIVMVLHFKDVQSLDDTSDVDESSELVDDSDAKE

>DmTsp68C NP_730317

MACCFNYKFVLNLCNFLFLICGLLLVVSGLYIFSDNKRILLSRLLAASSDRLSSLPQPLLFYIALGVAIAGFVATLAAVVGFWASCLHTYCFLTIYFLSVVVLLLTESVLCLAITLWPHCLGISLDETQMVRSLQSNYGVPGQEQFTNALDLAQVRFGCCGMRSSLDYDTSLWRLQGYGQRNWPVPLSCCFLKNAGHSMAYLDPKPANESMCQSLERLSYERERHTESCLPHLDNWYREQYSIFLGASLILAMIEFCVLLAIIMSCTGLASQRARLKKPVQEMRTQKVKSRQTLIENIYEPDVELRENSNHSGDGIYLGPASRHVSSEDFKELYIKPRDLYKQHNLRTSPANRPTQMRNYLV

>DmTsp5D NP_511054

MPATLAYAAHSAGSTSFYGQLCSCAFLGAGLWLRLSYAGYATLLPQHAGLSADTIFMGIGGTGFVVSFFGCCGAWVQSRCLLVLYFMLIVMLFMSEFLVGSIAFLFRGGLGRTLANELRFGIERHYNSSDRGSLVAPSVASIWDSVQQSFECCGVSSYEDWYDIQSWPGRRWVPESCCRTLYDQRQVLTEGSGDGMMRPDCGRSENPSLWWDKGCAHSLQSWFTGQLNVVGAVGLGIAFVQLFGLITSMLLFCTVKHKRASDTYKSYSPSIDPQTRTSSWED

>DmTsp97E NP_733176

MCGGFTCSKNALIALNILYVMIGFLLIGVGVYARAASIVTNLPIVGGILACGVILICISMLGLAGAVKHHQVMLFFYMIILFMLFLIQFSIASSCLAVNSEQQQQFAEQGWMTVPTDLRKQVQDSLKCCGFNATAPSTTSVVPPSNEPSCELINQQCCAHSSEPDCRCEPCGPLLEDKIDYAFKLCGGLGIFFSFTEVLAVFLARRYRNQHDPCYLPARAVFPHDYLY

>DmTsp47F NP_725044

MRSCGPSLIKYVLFAFNVLFAISGLGILIAGAVVLADVNEFNHFVEGRVLAPPIVLIVTGLIIFLIASLGCFGAIKESPTLLITFAVLLAVIFIVELAVGIAASVFKKDLEGMVKNSLQESIKRSNSEDTMAWDNIQQKLMCCGVDSPADWRTLSANKTLPGSCCQPQYIDSTVGHCLESPALGKDKYFQVGCVGKLKDRIEKNAIILIGVGIGIAFIQILGIVLACYLANSIRQERAK

>DmTM4SF NP_477248.1

MALPKKIKCFKYLVYSYVVLLALTGAAQIFLGTSLLWGHSVYYGIVQNKLWAPAAILLCLGPVTFILCWMGCQATNQRKRCLLGMFAALLVACICVQFIICGWSLAMRENLPTSVEIFIDDSFVEFLDKFSRTKVDNLHLWNRMQSQLQCCGVDGPLDYRRLSLPWSCCSRPEHAYESACDTHYKRGCLAVVSEQIRNRLLITAFGAAIIAIFQSLGIFCAVHLTILFGKNDNTHPMNMNRKKKQQQFLPLTIQDKRHDMPSPINLSPSAPGQRVLKTALPSAMHK

>Dm66A fromESTs CK661761 AI294005 AI293965 and CG16991-PB NP_729259.

MVDNEETGCLQRLLSVGKLQICKFIIYFVWLVNFIFSCADIYIYYFILKEHMPCWDCLFRSYMIIALTVNALMAPLLIVGFFFIYSHLCREIRIYATVLFLATWLQMMLTILFAQQYQIVGDVLRIWMNRKSLEFYESRCQCCGVLGPDDYKLGDLNIPKSCYKNGSERDEDLYRSGCSTRSIKPASPIIHVISFVIQYVLVICIEVFLIILLRSKSQPTSMWSERVTERFGSVKK

>DmTsp39D NP_523612.2

MASGGLTCVKYLTFFCNLLFALTGLLIFLVGGMVQLNYAHYSNFVSDHVWTAPIILMIVGAAVAVICFLGCCGALKESSCMILSFALLAVVIFLFEIGLGLAGYVKHTGLHQIMESQFNSTMQHYKERADYRDAWTLLQTELDCCGINGPNDWETVYRNSTLPAACCSVINLSEAKECTNTHATQHGCLQKLLEILDSKTLILASVVLGVAGIQELRSCLKCEVADFERKQQFLAYHASQELLQK

>DmTsp42Eg NP_523633

MACSTNVLKGFALFWDIILALFGLVVIGLGVHIIYKFEHFNTAAFVIIAVGVVVVLTALFGALGAARESSATSKVFVVILIVLVILEVLAVGFLWVFQTSLLINVDKTFDKLWNDQPVPIKPGNQSQIASLERWLDCCGNVGPSDYILPPNSCYNGESDKLNLEGCRQKFLDFIADRWTTFNLVSLVLLGVELICALLAYVLANSIVNRWRRSKYYQK

>DmLBM NP_523639.1

MGCATTSVKIASIVLNAVLGFLAAGAIGWIAYNADTETEEFVIAAYIACSLILVFALLGIFAAIRESVVLTATSAVFLLILAILQIVSTCLFLHEFDVKSGRDMVEVAWQANNMDSLQQKHECCGQSSAQDYIHLSLLIPPSCYADLQQTPDHLYLDGCIEKVQSFYESDKLRFIIVSWVLVAFELICFALAVFLAISFKNKQRRMEF

>DmTsp29Fa NP_523515.1|

MSLLTGSANAVKYTLFGFNLIFLITGIILIAVGAGVGAVYTGYKLFLAGKFFSIPTFLIVIGSFIIIISFFGCWGALKENYCLVLSFSVMLAIIFILELAAGISGYVLRNDASDLIKTSLTYSLNEYNSINPNATTKLWDDIQDEFECCGVTSYNDWITAFPNGDLPISCCNVHVGAVGTFTCNNAQSSVADRHKVGCLDGFSGYISAHAVSLGAAGVVIAILQFFGVIFACYIAREIKIRNGITGFM

>DmTsp42Eb NP_525011

MNCLSAMFKYLLYLLNLVFVAGGILLIVVGSIMLSTMGNFTAFDGGVNTQTIPICIIVIGSVTFVVAFFGCCGTIRENACCTTIYAICMLILFGLQLALSIWIFAANDKFLSSMGKAVDKAWDENNAAQGYPMDALQLAFSCCGNTGYQQYETVPSSCCGYKDRTKVCEAEIYSQRPGCRQEFVDFWASNTDLIRWSSLIIALFELGIFIMSCCLASAMRKR

>DmTsp42Ec NP_523629

MGCLSGIVNFILYIVNIVFLIVGILLIVLGSIMLSDLSRFDVAGSGTDPNTIPICVTVLGGLIFVVSFFGCYGIFRQSVCMTGAYTSMVFVLFILQLVLTCWVFVNRSAFLGDMSNLVNLLWDSHDYTAMGVLEETFGCCGDTSYTNYNNIGLSVPGTCCGYLDRQATCNTPSVYQSRPGCSAKFEEFWNDNMDIIRWSGLGLCIFDLVVFLIAGALTNCMRSQNAGRQVYA

>DmTsp42Ed NP_523630

MDCGGVFVKYVLFIFNILFVICGILLITFGSIMVSTIKDFSGVGETFTANSVAIIILVLGCVVFLVAFMGCCGAIRENSCALTSYSVVMLVLLVSQLALIIYVWVDHVQIQQSLEKIVQTIWDQRKTDALLMDTLQRSFKCCGLNGFADYGITYPASCCDSPSNGTCALTQVMTRSSCLKAVDSFWDTNVSIIKYAGLGVTAVELVAFIFACCLANQTRNSQRRQNY

>DmTsp42Ee NP_523631

MDCGTSMVKYILFIFNTIVSVIGILGIVYGVLILKSIGVVEVNGQVGFPIQALMPIILISLGSIVVFISFLGCCGAIRESVCMTMSYATFLLILLILQLTFVVLLFTHREEFENAMGNVIENAWNSEHTYKGGVFDTIQKSLHCCGSSSALDYIGKGDLVPPSCCSGSCLIPTNYYPGCRGKFVELMTTGSDNAKYVGIGLIGIELIGFIFACCLANNVRNYKRRNAY

>DmTsp42El NP_523638

MGCATGTIKYSLFLFNALWAILGILVLIFGGLGWGAMPDAYAIGILILGGTILVISLFGCCGAVRESPRMLWTYASLLLILLLLIVAFIILNPKDVFKKYALQTVENQWELEQTKPGSMDIIQKTYYCCGRDSAQDYLDIKFWNNTVPSSCCKDDSCVNPLNLYVRGCLIKVEEAFADEATTLGYLEWGLLGFNAVILLLAIILAIHYTNRRRRYNY

>DmTsp42Ek NP_523637

MGCTSGCVKCFLNTLNTLNALSGLSLIAIATLALSKAPIAYILFLYGLGGIIFVSAVLGCCGICMENVCMTATYGFLLLAQLIISLLGIFRFKFTEEYIEKFAAEEVQMKWDEELVEPGAMDIYQTVYECCGRDSPDDYVAIGRQTLPPSCYPQEDPQMPHYLAGCVQKSSENFVVLFSYAHDTNWIALGITILMMIAAFYLVGRFRKQRVRYTY

>DmTsp42Er NP_523644

MGWSPLMIRYLAFLFNFLCAVLGIATIVVNVIAIDQIAPKDQLILGLYIAVGSIVFLLSFFGCFGAIKESICVTWAYATSMLVMLIVSIVMLFVFRMHFEEDSITKLKQAFAKQTNTFDAMAEYQTQYQCCGIYKLKDYGDAYITVPSSCYDQNDTPYRDGCLAKMETQYEELLKGPKIVGWMLMVIEIGAFTFSTIMGVSLRNELRRSAY

>DmTsp42Ei NP_523635

MGLGATTVKHVLLLLNFVFSVLGLALIAFGIFFLISAAENAVSIGKNVAGGLIIALGVVILIIAIFGCLAAIHEAPVRLLIYVGAVVLLILAQLIFLGMSSHGTKDGISGSINEGFDRLWESERNQTGALSYYESWLQCCGVNSSEDYWIIHHGIPSSCCPESKCMDTPSRVFKTGCKAAFVKYLDDKLLVFKIVCWLLVIGEAVGAVFGWLLYSSVKNQSRRNNAVWM

>DmTsp42Ef NP_523632

MASTSSVKLIVYALDVLCTLLALVLISFGIYVAVSYNLNEIGQLTAYGYVGLGAAALLVVLWGYLSAWRENVCCTVTFIIFLCLVIIAQFAVVYLLITQEKTVASNLANALEATWEEELNSPGAMSLYQNWFQCCGRGSPQDYIVNERLPPETCFRNHDKSKPENLIHTGCRVEFENYWQHLTKIFNILALVLIGFELLLSVISCRLCNSIRNDARRSYF

>DmTsp42En NP_523640

MDCRTSFLKAVLIVLNVLLSLIGVTLIALSVYELNSSTPGTFEHIAIVVQIFVGTFVVLTSFLGCFATARVSLGLVWSYVICLLILLCLQIYIIAAAHSTDYVERSKKDFLATWADQRTNVERISLLEQKYSCCGQLGAHDYILMGRGIPLSCYKDQERREYSLFSGGCLQAVQAHATDNVAIGLIIKWLLLLVEFAALGAATHLGITVRNKLRRERF

>DmTsp42Eq NP_523643

MSCGTKALKVSSFVLDFLCCVLAALTIAACSYALIAFSHSVAIRVPSILGIVLGGLLFFSTIFGCIAALRESIRMTWIYAAILLALVFSQITVILAQPINYELLANETIYDAWQGQLYHSDRMSYFEIKYHCCGQTGPANYPDSGLVIPQSCYFNQNATVTTDLYTVGCNHQLAAAFVKGTRWEKITDWSVVGVEILTVIIAGLLAITLQNAERRRLYR

>DmTsp42Eo NP_523641

MPTVRVCLQWTSVVFSTLTLIVGVLAALAGVYELDKFNEGSAEHTEKFVQLGMAGALILAGLVGCLGAIFGSIKVMVVNLILLLALIASHIWKVSHYNETKQLDATEVYVMDLWMKELVHHGAMQDLQQEYECCGDKGFSDYTSLNMKVPRSCFHTKDGIHALYPYGEGCMAAVKRAYLQIYRYEKWVHCGLIGYEVVGIILGITLCCQLTNKTRRYTY

>DmTsp42Ej NP_523636

MEKSFPITPWKYGLLVTCILIVTCNVFFFSCGVTTWGSAVSVYGSYGSALCGGAVFGVAFLGMYVALKVSYKYSIYYLICSGLVIAALGSYLFTFTAMREQLMGRFEERMRDLFERKTHSDDKMQPVHSLFGCCGIEGPQDYLQEEHGALPSSCCYAFDCSKPAHVYEEGCSTKAVATLRMQAELNYYSCMAIIALEFLGLFTAYHLGKARKYAKTKIKDEETPIND

Principio del formulario

>DmTsp42Ep NP_523642

MNGCYNTIKYTGLLSNLLYMLLGIGVMSGAGLGLQMAEPNTPEHTYFVKSLVLGGSICMIVMFGCYGMVANLLCVNLIFTMFILIALAAEYLQLHHYHSPSLRSPGGAWQQLELAWHGLDRDPELMHQYEASQHCCGYNGADDYKRLHLLVPASCYQAAVNDTAQQIYPSGCLETLNRSQRYIQHRDKLYMWAIVGLEIFILLQTVALSVLLFRLRQRQRIARRQVPPGVRREPRSNHVSASRAHLLNDA

**SEA ANEMONE**

>1nematostella XP_001627980

MASCGMQCMKYLLFAFNFVFWIAGIAVMSVGIWTRVSANEYASLMGQSGFTAAANIMIAAGALVMFIGFVGCCGAVKENKCFLLLYFFLLLVIFVLEIASGILAYTKRDKVISSVEKALNQSISLDYGQSNKEKATKAVDRAQSIFKCCGATQGPKDWANTAWAKSNTNQTVPESCCKKKAAGCNGAPADSDTYYQKGCSQGLQDFIKSHMKTLGGIGLGIAIIQILGMIFSLCLFRSIRYEKI

>2nematostella XP_001628001

MCGIKCMKYLLFVFNFFFWLSGLAVLGIGIWTKVDAGQFDSFLGSSGYSIPAWTMIGAGAFVTFVGFFGCCGAVKESRCMLATWLKYLLTILLSLSKQVNLFELYLKFFLLWHQVQEEIKINLDEQIRNTYGYDSEVESHINNLQIRLKCCGGERPGDWLGSKWKRDLNKDKIVPLSCCTENANKTTCYKDDTYKEVYSSGCVNELKTFVDKHMILIGAIAVGISIVQLLGMIFSCCLFCAIDND

>3nematostella XP_001635890

MASGGMACVKYLLFFFNFIFWLSGLALVIVGAIIKSKYGDFIQVSSSSLASGPVFLIVIGVIVAVVGFLGCCGAYKENYCMVTTFAVLLAFIFVLEIAAGALAYAYKGKLEGYVRTGLKNGVDHYDKKEYKKAMDKVQGEFKCCGYEDSNDHGYKNSTVPKSCKDTNGTVFSKNCVKGLEKFLKDHLIVVGGVGIGIAFVQLIGIIFACCMMRSIKKEYEVM

>4nematostyella XP_001622937

MAAMKLGACMQCVKFLLFGFNALFWLMGLSVLAVGIWARIQFSDYMKLSSHDYATAAYILIGIGFLIAIIGFIGCCGALKEHTCLLKTFGVILGLLFLVELGTAITGYIFRSEIKTGLSDGLDTALKDYPEQGFKDAWNDMQKNLKCCGSRNYTDWFMVAWAGPNTKNGSVPESCCKDTKVKSCNLEVLSHPETINSEQGCYGAAVKYFEDKLVIIGGVALGLAVFQLIGIAFSCCLASTLHNNLKYELV

>5nematostella XP_001631910

MALQGSSNIVKILVIIFNFIFFLFGLILFGVGIWASTKLGAYVEIASVNYATGPRVVIAVGFIIALVAFLGCCGAWKENKCMLICFFAFLLLLLILEIVGGALAYNNKDKIENRLDKDILKAIENYPGKNEKSINDMQTKFKCCGADNYTDWQSNIKMKNSSSVPDSCCKSEKAGCGVGGVKDPKDIYTKGCFTIIKGEIEKSLKPIGGLAIAVLVIQLLGMVFALLLICRIKSETMA

>6nematostella XP_001622984

MAEGATKCIKLLVFFFNFLFFLFGIALVGYGAYAEIKFGPYISISSNDFMSGSRLLIAVGCIITIIAFFGCCGAWKENKCMLILFFILLLIVLGLEIAAAVLGYINKDKIQSDLTDDIIRNIKEYPKNNKAAIDAMQMDFECCGAKGPSDWTFYGQMKYKAGPGSCCGKPNNDACLPGTRNSKGCFEAMKDFVNDKLIIIAGLAIGLIVIQILGMILAMTLICRIHKGSYA

>7nematostella XP_001627383

MGSLDAGAGRILKILVVFFNVLFFIFGIALIAVGAWAEVQYGEFIEVSSVPYATGSRLVIAVGVIIAIVSFFGCLGAWKENRCMLGTFFVLLLILLTLEIAAAVLAYNYRGKVKDEIESDLTKALKGDYGSSGQDGVTKAFDALQEKQKCCGVNGYLDWKASKKYNGTSTVPDSCCVVKADGCGKAVNGINKGGCFNKMVEVVKDKLDVIGGFAITMLVIQANLGDNLCFSSHNQDWQDWRICIGSFSCVILESMTHK

>8nemarostella XP_001639655

MAMEGAANCIKYMVFLFNFIFFLGGVGLMGVGIYVQLKIGDYVELQSVKYITGSIIIIAVGAFIALVSFFGCCGAIKEHRCLLATFFALLFIILAVEITGTALGYVYRNKVEEQLGKDMNDTIVSYGEKGKEGITDAYDAIQKKEKCCGINGFKDWQRSYYTKGNHSIVPDSCCKTVSKGCGINFKENDIYTKGCFDTVKTLLKDNLIIIGGIGIGVAVIQILAMIFAMVLVCKIGAQSEYA

>9nematostella XP_001639656

MAMEGAAKCIKRTLFFFNFIFFLAGLGLMAVGIYVQLKTGDYIDLQSVKYATGYIIVIAAGALIALISFFGCCGAIKESRCLLAAFFTLLFIILATEAAGTALGFVYRDKVNQKLRQDMNNTLDSYGQKGKEGITLAYDLIQKMEKCCGINGYEDWQQTPFANGSSTLVPDSCCKKETKDCGSNFAQDDINTKGCLGTIMKLLKDNLMIVGGVGVGVAVIQILAMTFAMVLICKIGAQSEYA

>10nematostella XP_001639760

MVVLGTWTLIHRNEFNILLDSSWFVIIVGLMIGIGGVVVLICTCGCYGTVKEHRYLLISFLIMLTLVLIVQISVGVIAFVHRAEVLIHISPLTHDMKYTTRRRMSDYGVRQDVTKAIDQMQINFKCCGEDNWSTWNTTAWKQSSNNSVPDSCCKTPSFGCGKRDHPSNIYREGCVIGLTVFFRKHLLVLGSVLLGIALIQLIGLIMTVCLLRFVKDYY

>11nematostella incomplete XP_001639761

SGIAILAVGIWVITSRSQYNSLLSNDNYVTVPGLMIAAGCLVIIVCVVGCVAVIKENRFILVSYLIMLVLIFILEIATGVVAVIYRSEVSKISYELEVGIRGKMNRYGFTDAVTKAIDDLQKEFKCCGDRGMDSWNNTSWKQSEISGNNSVPDSCCKSPSPGCGIRLHPNNIQEFGCISKLEKFFLDNLAILVGVAFGLAFSQV

>12nematostella XP_001638950

MVFFFNLLFLLAGCAIFAIGVWIISSKDEVAGDYSRLTGTINYKTAPILCIVIGIVTVIVAFLACCGALKESQCMLGTYFGLVTAIFCLEVTAIVLAYVYRERIEKNLHGDIRSTMNEYHLSGHDAVTRAIDEIHRDFKCCGNAQYRDWFETKWAKTHPDMVPRSCCKDPSDSKCNENISKDPTKIYRRGCYMFVKKYLSNNLHVISGFGIWIAVIQLMGIIFATCLCCHIRTILSEDYA

>13nematostella XP_00162

MSCAKAFLFFFNFIYLGISAALIYVAVWLMQSYGNYSKITSDTYTIVPAGIIVGVGCLVLVTALVGICGTCRESKCCLSIFFILLLVVFSLEITAGVLGAIYKNQAKDEVKNGLTDAIDHYSKEEQGFQKAVDKLQENLDCCGSTGPNDWVNSTFWKEKHHLPKSCCTHDSDVCFPYDKFHNTKGCYTTLLDKFNKNLSYIIGIGIGFAVLQLIGMLSACCVMCSTGEVAYLRLDGGTRV

>14nematostella XP_001633494

SLCTKYTLFFINVIFFILGLLVMTVGVYMVKELKKDLKGVQDWLKLESTFHWPVVIFFVIGILMFFIGFFGCVGALRENTCFLTVYGTSVFLCLILLIACGAVAYTQKDKVQALKERLKQDLKKYLDDPLNQDLIDFMQTLLKCCGVESYKDWQANPYFNCSSPGRMSCGVPFSCCKESIQFNRQCGYDAGKLKTDAERLENGVYSGGCWPSIKGNLYTVIGITGAVLICIIFTMCMAFSFRAQIREVKKYARFQS

>15nematostella XP_001633495

MPTLLFFTGFSLYLIVFFGCVGALKENTLFLASYGIFIFCCLIPIIAAGVLAFLQREKLVDPKARLEDGLKSYQQDSSKQEFIDFIQTRFKCCGIESYKDWQANRYFSCSSPVPTACGVPFSCCKKSIQGNRQCGYDAGKLKTDADRLENGVYSKGCVVVVGFIKDVINLVIGVMFAGLIFIIVAMIMAFSFRAQIREVQKNARFQSELGIQRGF

>16nematostella XP_001634451

MAKVQPNRRRRPGQPDDPSLCVKYSLFAINVIFWLAGAVILAVGIFIFIEMKEEITKLADLNFQPAVIFLALGGLLFVITIFGCIGALRENRCLLTAYIWMCGIILAGMIVCGGLGFYYKDVLETKVTAQLKDAIVLYRDPTKGDLHLIIDTVQTELQCCGVQGLNDWDANIYFNCSGPARERCGVPYSCCRKDLQENRQCGYGARSVPTSQAKENGIFTEGCVKTGIAWINTNLYLIGGIGVGILILQLATICLAQTLKAQVNALVKFAKTQTF

>17nematostella XP_001627397

MSYPTKTTFCVKYTMFFVNVLFWLISCIILAISSFAMVNKKELYGKINNLATDPAVMLAIAGVLMFVISFSGCLGALRENVCMLRFYSIMLGIMLLLEIVAAVLGYVYAGEVKQQVEKAVDHIIVRYRDDPDLQNIIDLLQKELKCCGSKDYKTWEQNVYFNCSSPSVERCGVPFSCCISDQINSQCGFGAIRMSESQASKVIYTKGCIQGAESWFMTNLIMMASIAASLPATQILGYCLARRLIEDIRDIIRRQQQAWG

>18nematostella XP_001630351

MPKEHTEVSCCVKYLLFFFNVFFWLVGCLLVSVGLYARFEKTAYQEFFSDILTDPAFALIIVGGIMFILGFSGCIGALRENICLLKFFSVVLAIIFFLQLALGVFVFVFQDKVEAVIVEKLQTAVTKYRDNADLQNLIDGVQQEFKCCGAKGINDWDKNIYFNCSSPGSEACGVPYSCCIKDTINRQCGYGIRKSGTPTSDQSKIIFIGGCVNAVKDWFKSNMIIIGGAAVGIALLQIMGICFANSLISDIKMQKARWNRDYPSRY

>19nematostella XP_001638778

MDLHCLSKYFIFTLNFIVWVVSIVFIGVGSWAHSEKDKYNTMGNLVASPSIILIAVGIFMFLVAFCGCIGALRENRVLLKIYMVVLAVLFTLEIIAGFLSFFFVEETRSKVSSAIKLFVVHYQDDLDLQNAIDGIQSNLKCCGGYSYHDWNHNKYFNCSAQAVEACGVPHSCCVEDQINTQCGFGVRREKSVLEASSVIYVRGCVDSLSDWMVENLHIIGGLAFGFAAIQLLSILGASNLIKDIEQAIAISDKLHADAHNDFVYT

>20nematostella XP_001632787

MDNPNLEAQNVISKCLKYFLFVFNLLFWAIGGVMFGVGMWSVTQKGSYSKLSSLSTDPGSVLIAVGLLIIIISFFGTVGALREQIILLEIYKWVIVVIVILQVLGGLLAFAFWPNVRKSVQNQISKGIVNYRDNLDLQNIIDGIQENFKCCGSSSINDWDANRYFKCGGPSPEECGVPHTCCVKKDGEVQHNFKIQSSRHERERCFYIIGCLDAVINWFRDHMYVVAAIAIAFALPEFAGIILTHIFVQQIKEQI

>21nematostella XP_001631343

MALDGPKVVTFCILGFTALFLITGLGLIIFGSTLTTPFGEYFSLRKDAFDTQVGLRVGLILLILAISAVTILGAFKQNNRVLTILAVLFFVIFVTEISLGANSLTLRNEGISKSSTSASGLQLLSIYGEHPGVTEMIDNTQMGSMCCGVTDYRDWFTSGWAARQGVSNRVPSSCCKVPVPTCGWDMDVKNPVDVINIIGCSGLNYAQNDRLRVIGGGGLGLAFVHLLSVLSLIIFCFCFNTVGGLSPIDALKGKESEVQSGGGDSEGPLKSGPLGPGSSPKKKRKPKAVQEPFEDDDDLQENYSKDLLDKSK

>22nematostella XP_001622618

MVSLDASSMCLRWATFVLNAFILIGGSMMLGLAIWVTSEETEYNHVTGNLAASITYIAMSSILFITGCLGVCALLYMNPCLLKTYFSFMLILVVAEVTIAVYLLIEKDKIEDYITSNWNNTDDETRILIQTKLLCCGMKPLTTSHSSSSDKSCYEGMNKATGTRLTDCYSKLTQWIQNNYVVLASVAVVVAVAEIVRSKLRATVKTWYNQSLFVKDLSPNNSRK

>23nematostella XP_001637927

MYPPKYSKSHPCAYYCNKNSIVALNTLYIFIALLLIGITVYARVTAQITNLPILGGVIACGVFLLMIAIFGMIGAIKHSQVILFFYMVILLLLFIIQLSVSVGALAVTRNQQEQLMNKGWAKLSPSLKSKIQGVKDCCGYKNFTQLDGPMGHPDCSLLKCCSSLAKEPCSECTNCFDKLKDSVNHLLKVAGGFGLFFSFTLLFGVYMTCRYRNQKDPRANPSAFL

**TRYCHOPLAX**

>1Ta Cd63L[jgi|Triad1|63102|estExt_fgeneshTA2_kg.C_30005](http://genome.jgi-psf.org/cgi-bin/dispGeneModel?db=Triad1&tid=63102)

MALDGSAKCIKLLLFAFNVVFFIMGIALIVLGAVVKSTYGSVFTITNNALTSAPVIVIIIGCIIFLIAFFGCFGAIRESYCMITTFSIFMGIILIMEIVAAILGYVYRADVKKVLGSSFDKALHNYNNHNTSATAKAFDFLQRTVKCCGSNNYTDWFDARFDGNAISVPDSCCKTMSAGCGYDIGQNPNLNNIIYSMGCVNKLSQDVTNNLGIIGGVAIGIACIQIIGIVFACCLMRSIRSGYEQV

>2Ta Tspan3 [jgi|Triad1|54107|fgeneshTA2_pg.C_scaffold_3000114](http://genome.jgi-psf.org/cgi-bin/dispGeneModel?db=Triad1&tid=54107)

MAERDCSKCFLCFFNLIIWLVGVALIVFGGYLMGVYGDYTSVLNEQYTVLPFGIIIGVGVLFFFCAVLGWCAASRNSRACFGVFFFILFAIFAMEIAAVALGVFFKSNISDFIGKDLENAIKNYDTHNDHGIDRIVDKIQELFHCCGNHNYTDWSSSPFDHVYPASVPDSCCKNRTSGCGNDSLSLPRNQAGEIIYIEGCHDAIFNVAHDDMGIIIAVAASFAAIQILCMICTVFIMCRRSNDRLISGYDILGDGVVA

>3Ta CD63L [jgi|Triad1|61667|fgeneshTA2_pg.C_scaffold_26000047](http://genome.jgi-psf.org/cgi-bin/dispGeneModel?db=Triad1&tid=61667)

MGCGERCMLISFYIITFGFMVLGAALLGLGIYIRTNGNGLDIVVDTSWHTAAYIMIAIGVMVFLISLLGCLGAVRKSTCLLGTYLSCMIAIFIVELAGAIYAGINRTAVESTIRNKFSTEVKDSYGQPQKNAITTGIDQIQTLFQCCGIDSPFNWTNSKWSNSKPVGNYGPPVPDSCCKMEMPNCGTNPANHYNETIFTEAIYQQGCYIKIRDLISNNIAIAIAILATFGCLQIVGLLIARLLYSTLGYEYGYEI

>4Ta CD63l [jgi|Triad1|32798|e_gw1.26.51.1](http://genome.jgi-psf.org/cgi-bin/dispGeneModel?db=Triad1&tid=32798)

MHLRSGYQCIRIIFWCLNLIFTAIGIAIMAVGIYALLQYNQLPAFSDSTLKGGIAIAVAAGCLIIVIAGLGCCGAWAGNTCVLLLYTIILAIITAIEITAGIIGYVYRDTVRNYATRFISQVMSDYGNGHNATITTVNEVQQKFHCCGATTIASWYNSSFANGKPIVPDSCCVSMAKNCGKQGNITNIYNTGCLTKFIDYSKRNLLAIGGISLAVALLQILGIIFSCCLCREFHRQSAYTKVKLSVKGGQDVEAKIKIKNRGCLPC

>5Ta trichoplax MODIFIED PROFILING [jgi|Triad1|64199|estExt_fgeneshTA2_pg.C_120002](http://genome.jgi-psf.org/cgi-bin/dispGeneModel?db=Triad1&tid=64199)

MGKGSRDSDSEVSLCVKYTLFFFNIIFWLIGCFIVGVSIYARTEKGFQDGFASLAYDPVMILLVVGVVIFILGFSGCVGALRENTCLLRFFGIFLGLILLAEGTVAVLIFVYQAQVKAFVEDQLRGAIKKYRDDVDLQNLIDFIQRQFRCCGVVGYKDWDANIYFNCTKSNPSREACGVPFSCCRNSTINTQCGYDVRSQGASNSVIDRIYTTGCVDNVVAFLKDNLYVVAGVAIGIGVTQDYWLSNDKKPTSIDLLAL

>6Ta CD151l Chec TM[jgi|Triad1|63517|estExt_fgeneshTA2_pg.C_10884](http://genome.jgi-psf.org/cgi-bin/dispGeneModel?db=Triad1&tid=63517)

MGCGNLSRILLIVFNFIFVLSGFAVIGLAGYNLATYWDIATIAADSAVTIGTILLLTAGIITTLAGILGFCGSLNRHPGMLLGFFILLLLIFGFEVAAGIYTVVHRQEVIDALRTKLSNDIQNKYTPNNLGIQRGLNAIQTQFKCCGSSNYTDWARSTWGAGKTIAYPVPDSCCKVYVPGCGLDEVQRGGANLTGINLNGCVQSVSNTISENAYIILGVGVGIGALQIFGMVCSMILYCDLKEDKD

>8Ta Tsp7L [jgi|Triad1|52174|fgeneshTA2_pg.C_scaffold_1000921](http://genome.jgi-psf.org/cgi-bin/dispGeneModel?db=Triad1&tid=52174)

MPSPAKTSAGHKCLTILLHLFNFILWISGVAILAASIWGLIAFGPYMKLDNQHSFSALYILLGVGILIVLIGFVGCYGAMKGSRCLLGIYSVILIVIVLAEITLGIIAFVFKGDFEAGLAVTMKPFLQKYDTDVGIKHIVDGVQQNLECCGAQNYTQWFNTSWASKQKYRDSVPSSCCPKTFFTSGTKPNNSTGLCNNLHLTYNTGNYQYPSGIKISQSACSGAVIKFLESHLGIVAGIAVAIGVFQGDEQKDAIVDLLDYVNLV

>9Ta [jgi|Triad1|57319|fgeneshTA2_pg.C_scaffold_6000299](http://genome.jgi-psf.org/cgi-bin/dispGeneModel?db=Triad1&tid=57319)

MPSIETTNKRRQRHKKKWYKFEPTEISAWIRVPFFIVCLLTWLIGCISLGIGIWAEVQSANLRPTTANGKVNSQIANWTSGPAIILIVMGLMGFIMGFLGCVGALRENLCMLYTFLVSATIILILQIVAPILAYVLSQEATNLANTAVRSSITDYRANADLERLINYIQTSLKCCGGANYNDWEFNIYFNCTSPGPESCGVPYSCCINYTISQLQARTRIYTNGCTDSVKALIRNNLYWFIGIGFGLCLIQLAVLVLCGKLIQEIKAVKTDYEQYYNDVGTWPGGNTGENAGV

>10Ta [jgi|Triad1|19533|e_gw1.1.805.1](http://genome.jgi-psf.org/cgi-bin/dispGeneModel?db=Triad1&tid=19533)

MGCCTICSRFFLIVISIVMILFSISIIIAAAIILSQSDNIAAALPNNIRTVTVSAILVIVACVMMIFSGFSGCGGAFKRNSKPLYVYVVFAAIVLAMSIAGVVIAAINSPQISSAIGQGLTNVATNEYGDPNRQSITTAMDRLQRQFQCCGGNSHLEYTNSLWANTSATNATNSNPVPSSCCINQQSRCGQDRISNSVIYTNGCTTGSGLSAQFILNSVVGMGIAVCVIQGLAILCALILLHDLVYNEGKGTHSTTKVVIIKEQAS

>11TaTsp[jgi|Triad1|63519|estExt_fgeneshTA2_pg.C_10886](http://genome.jgi-psf.org/cgi-bin/dispGeneModel?db=Triad1&tid=63519)

MGSCTNCLRITLIAFNTIFLLSGIALIGASAYIFATSADITTIATDGGIYVTGTALVMAAGILTFIASIFGCIGAMKRNSCMLITYFIFLLIIFAMELGGGIYAATNSPAIINAITNSFRSDISSKYGVGNNGPLTTAINHIQQQFKCCGAQTYTDYTGSEWGKQSNATIANPVPDSCCRTVTLNCGLNRTDNTNIYTNSCRVQLTNAVAENIRIVLGVGVTIGIIQVFGMIAAIMMVCDIKKEGEVV

>12Ta [jgi|Triad1|38462|estExt_Genewise1Plus.C_260068](http://genome.jgi-psf.org/cgi-bin/dispGeneModel?db=Triad1&tid=38462)

MNGCGFKFMKHLLFIFNFIVWAMGIALLAMGIIIRVNSDLIGVVADNSWFTAAYIMIAAGVFIFLVALVGFCGARNSNKCMLGIYLGVLVVIFILEIAGAIYGAVNRVAIEEAAKAKMTTEVQIRYGQANQGVITDGFNKLQTTFKCCGVNTTTDWYSSAWISGKPSFPYGPAVPDSCCRTSTLLCGVNSTAHANAVIFNATYYSIGCYQAVRNSITNNLGLAIGVLGGFAGVQVLGMILAGVLIAKADEEE

>13Ta [jgi|Triad1|63518|estExt_fgeneshTA2_pg.C_10885](http://genome.jgi-psf.org/cgi-bin/dispGeneModel?db=Triad1&tid=63518)

MADCGDLVRIILIVLNTIFFLCGGAMIGLAIYVLVVSGTTITTALGSSGLILPGVILLMVAGIVTFICSGFGCCGANKRKRWCLTSYLIILFVIIVLEIAGGVYAYVNRDLITTNLTSRLNSDLATYYNTPTTQTAFNTIQSGLSCCAVHNYTEWRGTSWYGNVSAVSTVTYPVPDSCCRSNVSDTASCGYTYSTTNNIYTTGCLAKLQTLASDNASILLGIGIGVGVVQILGFIFGVILCNSIKND

>14Ta ESTs CD151 [jgi|Triad1|59701|fgeneshTA2_pg.C_scaffold_12000052](http://genome.jgi-psf.org/cgi-bin/dispGeneModel?db=Triad1&tid=59701)

MASKPKWTAVDYAKRVAIFFNFLLVICGLSMLIFGAWINATNSEFHIYTGDGGYISTSVLLIIGGLVIAVLGAIGCGASYRGSCRLILVYFGILSTLFLIEVIIGCVIYGQHDSMMLAAQNGLNLTITSQYGFNQPVTNSIDRLQQRRKCCGTKSWMDWRYSLWRKNESLHDRNFVPESCCITVSRSCVVTTSPSNIYYKGCGTALSEFVDENVYVIGGVCFALGFIQAIGIILTFYYYLKLREA

>15Ta [jgi|Triad1|58643|fgeneshTA2_pg.C_scaffold_8000191](http://genome.jgi-psf.org/cgi-bin/dispGeneModel?db=Triad1&tid=58643)

MKISTYEDKGVACAQYFLLFFNLMLWVVGGIMFGLGIYFHVHHDVYSTLTSETYFIGANLLIAGGVLLIVGGFFGCCSTLWDNQLALIGFIFYLVICLSLEIATAVFGYQGTPSLQAIALKHMTQDVDTKYHTMKNVRDAMDRLQSTRHCCGYNGYQNWAASTWAKNGTADGKLKGPYPVPASCCNTTMVKSNSPAACTKLYDYGKNTQYIYTQGCFPRYKDDIVMFMYSIGAFGIGVSLFQILGIILAFVLFFSLSSTQGYKYI

>16Ta Check ESTs[jgi|Triad1|52140|fgeneshTA2_pg.C_scaffold_1000887](http://genome.jgi-psf.org/cgi-bin/dispGeneModel?db=Triad1&tid=52140)

MPRKNAVVGATMLGMGIYYHIAHTDYAVLTSESYFIGVNLLIAGGTLLFAAGFLGCFSALCDNQALLVGFILALILLLGLEIGTAIYGYVGTPTLEGIAKKHMTQDVETKFRTNVKVQKAMNNLQKTRKCCGVDTYKDYAKSSWALAKFNNSESDLKGNFPVPDSCCNTSIVGEDLPRCQKEYANGKNVSYIYADGCYPRYRDDIVQYMFSLGAVGVSMTLLQIMGIMLSFILCCSLNGSQGYGPLPLKRDPYDY

>17Ta TaTsp13 2 EST profiling EDV27750.1

MANFPYSCVRNSLGALNVLYMIIGLVILGVAGYARGVAKFTSLAALGALVAVGVILLLIAIIGFVAAIKHHQILLFMYMIFMSLLFIIDFAISIAALALNQQQQKDLLSKAYDGLTPEDKTALHNRLKCCGFNNSTNIDCPSSCDNSCKTCYNVVKEPIALALKAGGGVGLFFSFTF

FLAIYFARQFRNLKDPRSSDSFL

**SPONGE**

>1oscarella1 EC370499 EC370133

MGSDHDLSGGMKIVKYLLFIFNFIFWAGGVAVLGLGIWLKIEYEDFLEIADQDWANAANIMIAAGCLITLLGFLGCIGACCESRVALLIFAVLLFITFLVEIVAGILAAVYRNEVEESLTKSLTDSINNDYNSSEPTRTAWDNLQKQFSCCGVNGSADWLDANLPIPDSCCETTNTTCNAANPALYKTGCYTDLKSFLEDILAAVAGVGIALAVIQLLGIIFACCLANALK

>2oscarella2 EC370778

MGGDSDLSCCGKFIKWALFVFNFILWAIGTAILGLGIATYIKYGNFLDELANKSWLSAPIIMMAVGSVMVVICFIGCCGAWKESQCMLYIFASFIIGVISVEIAAGVVGYLKRDELETTLQKELLNSLKEYNITPVAEAWDFAQKEFKCCGANTTYREWWTVTNFFPINHVPFSCCNPGNNTVCPTFGQYETTGQVTWYEPGCYQKIKQDIEDNYVPVLIALIVLACL

>4oscarella Tsp4 EB741452

MGSTELSGAWKCCKYTVFFFNLIFWIFGLALIIVGAIALDKFGPIFSLDSNQKWSSGPALIIAVGVIIFIIAFAGCAGAFLQSRPLLYTFSFLMGIMFIATIVGVILVVVYKGQIEDHLRSAMNKTMVEYSEENEGIIKTWDGMQHDFPKCCGTTNYTDWYVLLGQDTVPDSCCLKMEKGCGNNLLSNNTADAVIHSEGCYNKTVGAVEDHWPAAAGIAAAVAIIQLISVIISCGLARAISAGKYEVVZ

>6oscarella6 EB741453; EC369375; EC369002

MPVLEGCTCANTIKWIHFTFNFIFWAIGLAILGLGIWLYVTIGDFLDDLAGVSWLNAPIILIAIGCIITVLGFLGCCGACMENKWCLYIFGVLLTLTLIVEIAGGAVAYAKRHDLEDELESSLLGSQLDYNSTTVIKEAWDTVQKKFKCCGTLGPANWTDETGFPGGSLPATCCLNEPKHCNVAVDHYPEGCYEAVKEEVADNIIPVGVAITVLGCVQIVGIIFAFYLGRVISKEGKAEFV

>7oscarella7 EC3757618

MGKGRGEDDSEVSLWVKYTIFAFNFLFWIVGSLILGVGLYAKFDKGWGNLSDTFSTDPAIIMIVIGTLIFLIGFFGCLGALRENVILLKAFVWSIGIIFLLEVVAAVLAFVFRSQVEEGIKTLIEKAVKQYRRDPDLRNLIDFTQEYFECCGGEDQNDWDLNEYFNCSSPTPEACGVPFSCCMTADERINSQCGYDSRKLTGNTLNNANIWSVGCWDAVIAWAKENLILIASVALSIAVLQIVAIGCGCSLISSVKRQEMFYZ

**MONOSIGELLA**

>monosiga EDQ86250.1 jgi|JGI_XYM13072.rev|

MTSETKDKSGFSVALLWLYVFNGIYLVLGLTLIGVAAAAQAEAVLTDLTIL**G**GVIAMGVFLVMVSIFGIVGTARRSPFLLFLYILFMVLLFVMQFSIGVAALSVSQTQQKQLLSEGWCKLSDERQIWFQNSTNCYGFENASKYEPNDCMAPPPQNASNPKCPTRCAAPECGVEPTDTLPRCETCYDAVK**G**HLLMSNMASGLTDRLLRRGGGICLAFAFLELLGVVAAIRYRRDARAPATYNAFM

**FUNGI**

>Phanerochaete chrysosporium (genomic profiling with ESTs from *Antrodia cinnamomea* DR032716; DR028174; DR028019)

MAFYGFVDLWLLAAGLLSVIMSFVWRAPNLMLNFTISNSDLTGACTGYIGLNRTDAHTAGLVLGIMLLFTFFISIIAIAQRNHVTSGLVFLNWTLIGDAIAVLVIGTFIWFYSLQQRNNYYEVFKVQTADTRRAIQDKFSCCGYMTPNETFTVLGGFCANQTFVDSLFNKSAPDQNACVGPITAFTDFTLNNIFTIYGFMAIIISLFLASLCVIHQVLSRSWCYSCYVR

>Encephalitozoon NP_586477.1

MHHKSTVKKTFKVLFFFIQALGFISGIGVLIVGTTIYATAHEILQIPTKMLMLSYVLGILEVLSAVLGYTALASRRRLRMLVYISTTLILMNVQAIMAVKSTVIHERSRAWADWRWDSLNEEQRNFVQSKFKCCGFLDSSDRSGSSCGGSDGCVDAVYKLSKSTASLMQRTLMFSFFFESVGMGILSMLRLRK

>Neurospora crassa PLS1.Nc XP_964619 MSKVLLAYVVADGLFLLMGIFMIAFSVIVQNIQFEVPTEGQQAARNLLYQRFPLTAGIVNAVFIFVTFLFTIPGIITPARGWLKLGGWMTTVCGIFSLIIGLYLWIMTLKTKADFAPFYFSQPPEIQELMQSAFKCCGYFNSTSPAFITDDICSSPAAAALMRPCATPITSFANVLIDNIFTAVFGMVGIDVVLVMATACLLKERKERERFRHIDEKSGAIGF

>Gibberella XP_388871.1

MVDKIFLTTVCADILFLGSGVMELVFSLVVRSQMNDMATDGESATRNLLYQRFPLTAGIVNAIFILVTFAATLPGLVMPARSFLKVSGYMVTVCSIFTMCVAVFLWVMTLRMKEQFFNIYIEQDPDVQSLIQNSFQCCGYNNSTSPAFVMDSTCTSPASSALLRGCATAISSFANLHIDGIFTVLFGLVGIDAIFVLCIACLLKDRKERE

RYRHIDEKSGYRQI

>Blastocladiella CO967140 CO962469 no genomic sequence

MAPPLASWFSRVNRIRGTIWILKNTVMLINVLSLVSSVGFFAVGIYGYSDSSVVGIVSSSLPLACIILGIIVALISLIGVFSVANESALFIRVYFALLLLLILAEIIIGGVAFSHKDNVELYLTQAWTNAYKTDKSTIARLQGFFGCCGFRTVDDMAVRDKCMTMVPCYTKVKQQFLTGLNTMATVAVTLGVIELFCLLADGLLVFFSRLSEDA

>Rhizopus Zygomicota genomic profiling with PLS1

MMAIQLKISIAILIPINILKSMNKCLFCKCVCTIILYICIVINILGIILIFSSLIGLLGSFYREKKSIHFLCTAVVVIAFVYQVSIAVIVYKQAAHTTSWISQTWAEASSDYRLYAQTKFHCCGFTGPMDHPVSSDTCIPHQVIDSAPPCYGPMNQYIKQELTHIYIVLFTSLVIELLALCNSITQLCTMNPNRTREVSPEYVLEERKYTNMNEYNASADTLVNPVYSKYKRQF

**AMOEBOZOA**

>Dictyostelium-1 XP_646376.1

MVDTSNLLPQTPRLLKVPLIILNIILWILGLVLVIVGGICVSFLSNFKDFTKASDAKSALSNLTTSIPAGVLVIGILFVIFTVVGCFVAYKEKLVGLVIYCAVMLILLVILIGVGGKAITLHNDDIINEVGGAWEHVANGTKNSTLTRLENFLKCCKWSNVSIDSSDLCPKDGDKIKYEGHYCGEALSDQFSSKIYAVGAAGLAIGIIELVAILFSLFLIIRICRSPRTRSYDQY

>Dictyostelium-2 XP_646364.1

MVDTTNLIPNTPRYLKVPLIAFNTILWVLGLVLVIIGSIGVSFFSNFKDFTKVSKASAALSNLTTGAPAGVLVIGIFFVILTVIGCFVAGKEKLVGLVIYTMLMLIILVALIGVGGKALTLHNDDVVKQIGNAWEDVSNGPKNSTILKLENFLKCCYWNSTSSRNPLLCPKDSKGIPKYTDTCDSVISSKISSNLYLVGAAAVSIGVIEFICMLFALFLIIRICRAPRTKSYDYQ

>Dyctiosyelium-3 XP_646377.1

MVEYLPSTPRYLKVPLIILNVILWLLGLVLVIIGGICVGFFSRFKELQEVGGVSESIKSISVSLPAGVLSIGIFFMVLTVAGCIVAYKEKMVGLVFYTILMLVLLVVLIGIGGEALTYHNADIGIEIEDNWKNISYSNQSVVIKKLEQFFECCCFDESDLKLNCTALCPQDDQKNILYNGTFCYDVIFGAVNSKLYLVGSAGVAIGVIELVSLMFALFLIVRLYKSNSYR

>Entamoeba histolytica EAL42794

MEKSRIFGICFGIFLIISGISVEIVSAFGGFNKIIDSKKSMIKYSKTFFRGFNYAIGASLFICGILLIIASCLMNGFFINGGGIINVLFLIFVIGSISYFSYDLRQLFTMDEEKAKPFQIPYDCCGWKLTNITTLNNSTCQSPIGIKTGKTCFQVLKDIEYADYQEIVVMMYSIVLILISESSYRLLNETLQQQNTNQEVSILPRITDEITDNNEEYANDSNDNINSPLLINSQEGITQRISESTTTNSEDYLV

**STRAMENOPILES**

>Phytophthora ramorum genomic profiling with ESTs from *Phytophthora infestants*

MHAWRSLSRSILVFTNVLFLLLGSVLVSIGGYMASLPALTEFSDGGVASSVIMCGSLIILIALLGCCGAQWESKVFLFPYAILVLVSVIAQLSLAGFLTHVHSSLVEVAKHNFDLSVLGPADQETLRWINKRFKYVYYGCGFDVDIDLTGTRGRPLVATCSNPEFAWFAPFVEENCPIGHKQLQTGSNFLKCAGPSFSLSNAMTEHTMLCACETRMISWVNDQSLLVAVFVFVIVALEIMLVALSCYVMHSRRHRRYGYQEITMPVRQQPYNPHPRNYFGQQASQRQPLNSQPAYTSYGPSGGENPYAAAAAGGKAKKAGY

>Phytophthora sojae genomic profiling with ESTs from *Phytophthora infestants*

MHAWRSLSRSILVFTNVLFLLLGSVLVSIGGYMASLPALTEFSDGGVASSVITCGTLIILIALLGCCGAQWESKVFLFPYAILVLVSVIAQLSLAGFLTHVHSSLVEVAKHNFDLSVLAPADQDTLRWINKRFKYVYYGCGFDVDIDLTGTRSRPLIASCSNPEFAWFALFVEENCPIGHKQLQAGSNFLKCAGPSFSLSNAMTEHTMLCACETRMISWVNDQSLLVAVFVFVIVALEIMLVALSCYIIHSRRHRRYGYQEITMPVRQQPYNPHPRNYFGQQASQRQPLNSQPAYTSYGPPGGENPYAAAATGGKAKKTAGY

>phytophthora infestants CV901333; CV951114;

MHAWRSLSRSILVFTNVLFLLLG**G**YMASLPALTEFSDGGVASSVIMCGALIILIAMLGCCGAQWESKVFLFPYAILVLVSVVAQLSLAGFLTHVHSSLVEVARHNFDLSVLAPADQETLRWINKRFKYVYYGCGLDVDIDLTDTRSQPLIASCSNPEFSWFATFVEENCPIGHKQLQSGSNFLKCAGLSFSLSNAMTEHTMLCACETRMISWVNDQSLLVAVFVFVIVSLEIMLVALSCYIMHSRRHRRFGYQEITMPVRQQPYNPHPRNYFGKQQAGQRQPLNAHPAYTSYGPSGGENPYAAAATGGKTKKTGY

**PLANTS**

>Lycophites selaginella moellendorffii genomic profiling

MGLSNYLTGILNFLTLALAIPVIGAGIWLSQRHDTVCMRFLQGPVIAIGVFILVVSLAGFIGSCFRVSWLLWIYLFVMFLLIVLLLAFTIFAFAVTNRGAGHALSGKGYKEYRLGDYSTWLERRVKNTGNWNRIKSCLADAKSGCCKPPTACGFVYQNATSWINSASPAADTDCFAWNNAADRLCFDCNSCRAGVLENIRKDWRKVAIINIIVFVFLVVAYSVGCCAFRNARRDEYFSNKAPYR

>Moss-1 Physcomitrella patens scaffold_191|XP_001775454

MGCSNVVTGVVNFLMLMLLLPIIGFGVWLAKKHDSECVRFLQWPVIVLGMFVLVVSMAGLFGSWCGNRPLMWTYLFVMFVLIFLLFVLTLLAFVVTNSGAGRVVSGKGFKEYKLGDYSNWLQKRVDNPLYWSKIKSCLADGQVCSDLSQYATADVFNNASLTPSGCCKPKSDCGYTFQNATTWLGNSSGSANADCRAWSNTQTQLCFDCNSCRAGVLQNVKSNWRRVAVVNIIVLVFIIFVYSCGCCALKSSKREQDNFKYRYGYA

>Moss-2 Physcomitrella patens scaffold_2 XP_001751676

MGCSNGLTGFLNLLTFLLSLPIIALGAYLAKTHDSTCMRFLQYPIIVIGVFMLLMSLAGMIGAWCDKKFLLLIYLFFMFILIVLLFCFTIFAFVVTNSGAGSAVSGKGYKEYRLGDYSNWLQKRVDNPSTWEKIRSCIQDSKVCSDLGKKYTTETDFNKASLTPLEVNCFTNFLYFQKSGCCKPPTACGYKFVTPIEWTGTNSTADADCGTWKNTPQEWCLGCNSCRAGVLQNVKSNWRRVAIGNIIVLVFLVIVYSCGCCAYRNNKRYDKGYA

>Moss-3 Physcomitrella patens.1scaffold_303 XP_001781448

MAFSNVVMIVLNFLSMILSLPIIAFGVWLAKKGDTECVRFLQWPIIVLGVFVLVLSLSGLIGSWCGNRVLMYSYLFIMFLLILLLFVFTIFAFVVTNSGAGKTVSGKGYKEYRLGDYSNWLQKRVDNPKYWSKIKSCLVDGKVCSDLTKYTSAASFSKAPLTPLESGCCKPPTECGFTFDNATTWVGKPPSTVSNIDCGQWSNIQTKLCFDCSTCRAGVLQNVKSNWRRVAVVNIIVLVFIIFVYSCGCCALKASRRERANHKY

>Moss-4 Physcomitrella patens scaffold_22 XP_001756386

MGCSNYLTGFLNLATLVLSIPIIVFGVWLSKTQDTVCVRFLQYPIIAIGVFILLMSLAGMIGAFCDKKILLLLYLIVMFLLIVLLFCFTVFAFVVTHSGAGNVVSGKGYKEYRLGDYSNWLQRKVNDTAYWSKIESCIADSKVCNNLATKYTSVDAFNKAALTPLESGCCKPPSDCNFIFGKNATDWVGTGSAAPDTDCRSWNSQDLCLKCNACKAGVLQNVKSNWRRVAIVNIIVLVILIFVYSCGCCAYRNPERVGYRKSY

>At-4 *Arabidopsis thaliana* NP_190146.1

MRTSNHLIGLVNFLTFLLSIPILGGGIWLSSRANSTDCLRFLQWPLIVIGISIMVVSLAGFAGACYRNKFLMWLYLVVMLLIIAALIGFIIFAYAVTDKGSGRTVLNRGYLDYYLEDYSGWLKDRVSDDSYWGKISSCLRDSGACRKIGRNFNGVPETADMFFLRRLSPVESGCCKPPTDCGFSYVNETGWDTRGGMIGPNQDCMVWSNDQSMLCYQCSSCKAGVLGSLKKSWRKVSVINIVVLIILVIFYVIAYAAYRNVKRIDNDEPAGEARMTKSHPSHFHL

>At-5 *Arabidopsis thaliana* 5.At gi 7269944|emb|CAB79761.1| senescence-associated protein homolog [Arabidopsis thaliana]

MVRFSNSLVGILNFFVFLLSVPILSTGIWLSLKATTQCERFLDKPMIALGVFLMIIAIAGVVGSCCRVTWLLWSYLFVMFFLILIVLCFTIFAFVVTSKGSGETIQGKAYKEYRLEAYSDWLQRRVNNAKHWNSIRSCLYESKFCYNLELVTANHTVSDFYKEDLTAFESGCCKPSNDCDFTYITSTTWNKTSGTHKNSDCQLWDNEKHKLCYNCKACKAGFLDNLKAAWKRVAIVNIIFLVLLVVVYAMGCCAFRNNKEDRYGRSNGFNNS

>At-7 *Arabidopsis thaliana* NP_850045 MARCSNNLVGILNFLVFLLSIPILAGGIWLSQKGSTECERFLDKPVIALGVFLMVVAIAGLIGSCCRVTWLLWVYLFVMFLLILLVFCITVFAFVVTNKGAGEAIEGKGYKEYKLGDYSTWLQKRVENGKNWNKIRSCLVESKVCSKLEAKFVNVPVNSFYKEHLTALQSGCCKPSDECGFEYVNPTTWTKNTTGTHTNPDCQTWDNAKEKLCFDCQSCKAGLLDNVKSAWKKVAIVNIVFLVFLIIVYSVGCCAFRNNKRDDSYSRTYGYKP

>At-11 *Arabidopsis thaliana* NP_179548

MALANNLTAILNLLALLCSIPITASGIWLASKPDNECVNLLRWPVVVLGVLILVVSATGFIGAYKYKETLLAVYLCCMAILIGLLLVVLIFAFVVTRPDGSYRVPGRGYKEYRLEGFSNWLKENVVDSKNWGRLRACLADTNVCPKLNQEFITADQFFSSSKITPLQSGCCKPPTACGYNFVNPTLWLNPTNMAADADCYLWSNDQSQLCYNCNSCKAGLLGNLRKEWRKANLILIITVVVLIWVYVIACSAFRNAQTEDLFRKYKQGWV

>At1g18520 (F15H18At) *Arabidopsis thaliana*

MFRVSNFMVGLANTLVMLVGASAIGYSIYMFVHQGVTDCESAIR

IPLLTTGLILFLVSLLGVIGSCFKENLAMVSYLIILFGGIVALMIFSIFLFFVTNKGAGRVVSGRGYKEYRTVDFSTWLNGFVGGKRWVGIRSCLAEANVCDDLSDGRVSQIADAFYHKNLSPIQSGCCKPPSDCNFEFRNATFWIPPSKNETAVAENGDCGTWSNVQTELCFNCNACKAGVLANIREKWRNLLVFNICLLILLITVYSCGCCARRNNRTARKSDSV

>At2g03840 (SASpl3At.) *Arabidopsis thaliana*

MARDKEDQNNENPSIVQNMSFPFNTIFLISSAIFLVTAAFWFVA

VMTLHYRTDECNRFVTTPGIFISFSLLAMSLTGFYAAYFKSDCLFRIHFFIFFLWMFVVVSKAIFVIFLHKETNPRLFPGTKIYEFRYEDYSGWVSRLVIKDDEWYRTRRCLVKDNVCNRLNHKMPASEFYQMNLTPIQSGCCKPPLSCGLNYEKPNNWTVSRYYNNLEVDCKRWNNSADTLCFDCDSCKAVIIADVHNTSFSITVNIIHIIFSLCIGMTGWFAWLRILRES QK

>At3g12090 (SASpl4At.) *Arabidopsis thaliana*

MYRFSNTVIGVLNLLTLLASIPIIGTALYKARSSTTCENFLQTP

LLVIGFIILIVSLAGFIGACFNVAWALWVYLVVMIFLIATLMGLTLFGLVVTSQGGGVEVPGRIYKEYRLGDYHPWLRERVRDPEYWNSIRSCILSSKTCTKIESWTTLDYFQRDMTSVQSGCCKPPTACTYEAGVVDGGGDCFRWNNGVEMLCYECDACKAGVLEEIRLDWRKLSVVNILVLVLLIAVYAAGCCAFHNTRHAAHPYHPSDDNRMTRVRPRWDYYWWRWWHEKKEQLY

>At4g28050 (SASplAt) *Arabidopsis thaliana*

MVQCSNNLLGILNFFTFLLSIPILSAGIWLGKNAATECERFLDKPMVVLGIFLMFVSIAGLVGACCRVSCLLWLYLFAMFLLILLGFCFTIFAFAVTNRGAGEVISDRGYKEYHVADYSNWLQKRVNNAKNWERIRSCLMYSDVCSTYRTRYASINVEDFYKSNLNALQSGCCKPSNDCNFTYVNPTTWTKTPGPYKNEDCNVWDNKPGTLCYDCEACKAGLLDNIKNSWKKVAKVNIVFLIFLIIVYSVGCCAFRNNRKRSW

>At5g23030 (SASp5lAt.)

MLRLSNAAVITTNAILALIGLAALSFSVYVYVQGPSQCQRFVQNPLIVTAALLFFISSLGLIAALYGSHIIITLYLFFLFLSILLLLVLSVFIFLVTNPTAGKALSGRGIGNVKTGDYQNWIGNHFLRGKNWEGITKCLSDSRVCKRFGPRDIDFDSKHLSNVQFGCCRPPVECGFESKNATWWTVPATATTAIIGDCKAWSNTQRQLCYACESCKIGVLKGIRKRWRILIVVNLLLILLVVFLYSCGCCVRKNNRVPWKRRFF

>At5g46700 (SASp5l1At.) *Arabidopsis thaliana*

MPLSNNVIGCINFITVLLSIPVIGAGIWLAIGTVNSCVKLLQWPVIILGVLILLVGLAGFIGGFWRITWLLVVYLIAMLILIVLLGCLVGFIYMVTIRGSGHPEPSRAYLEYSLQDFSGWLRRRVQRSYKWERIRTCLSTTTICPELNQRYTLAQDFFNAHLDPIQSGCCKPPTKCGFTFVNPTYWISPIDMSADMDCLNWSNDQNTLCYTCDSCKAGLLANIKVDWLKADIFLLLALIGLIIVYIIGCCAFRNAETEDIFRKYKQGYT

>At5g60220 (SASPl2At) *Arabidopsis thaliana*

MRSRSNLIGLINFFTFLLSIPILGGGIWLSSRANSTDCLRFLQWPLIIIGISIMVISLAGIAGACYQNKFLMWLYLFTMFFVIAALIGFTIFAYVVTDKGSGRFVMNRRYLDYYLNDYSGWLKDRVTDNGYWRDIGSCVRDSGVCKKIGRDLNGVPETAHMFYFRNLSPVESGCCKPPTDCGYTYVNETVWIPGGEMVGPNPDCMLWNNDQRLLCYQCSSCKAGVLGSLKKSWRKVSVINIVVVIILVIFYVIACAAYQNVKRMYNDEPVGEARMTNLILVIFKFKEILVQFFFGIVFLLLFNGLMVCCCNDKFAFSVFFFGYVTYA

>Os-5 gi|37806167|dbj|BAC99671.1| putative senescence-associated protein [Oryza sativa (japonica cultivar-group)]

MAPRCSNAVFAAINVVTLLLGAAVLAAGIYYGAPHRGGGGVTECERFLRAPALALGGAIVAVSLAGLAGACCRATPLLWAYLLLTGLLILAAACFGVFALVVTNAGAGRAVSGRGFREYHLGDYSTWLRRSVEDGGHWARIRSCLVDTGVCRSLKSNQTLDEFVNSNLSPLQSGCCKPPTACNFTYQNETYWIKPPTPSNYSDPDCNSWSNDQSELCYGCQSCKAGVLGNLRSSWKKIAFVNAAFVALLLVVYSLGCCALRNNRRHKYSLVGK

>Os-7 gi|57863801|gb|AAS72369.2| unknown protein [Oryza sativa (japonica cultivar-group)]

MLRGGTSLLGIVNFVTFLISIPILGGGIWLASRANSTDCIRFLQWPIIAIGLAVMVVSLMGFAGACYRQTWLLRLYLFAMFFIVVALLFFIVFAFAVTDRGDGQVVMNRRFLEYQLSDYNGWLRDRVADPAYWATISACLRDGRACAAMRRFARDPNTGMLVPETPSMFYARDLSPIQSGCCKPPTSCAYNYVNETFWTANPGVPTVVNDVDCSKWSNDQQTLCFQCDSCKAGVLAGIKKSWRKVAILNIVVLIILVIVYVAGCAAFRNARRIENDEPFGMARMTKTQPSRFQF

**ALVEOLATES**

>tetrahymena-1 Q22W66|Q22W66_TETTH Tetraspanin family protein - Tetrahymena thermophila SB210

MKQKKVSKFVQSQQMKKNKQSTQFLNMSVCLNFLKGYLVLGCIFNIGLGIGAFVAAAYLNNLNQSFLVNGGVSAGKKYGVIGLFALGGVCCLIGLLGIIAFWKRIKCFQFLFILFNTLFLVIFAAVLAGLIYSQPFLNKLKNQSCDQITELKEADDLFTRQMKPYFCKLQTSSVCPCYVTDKSKWSSVDTSQIVFQPSSSSKDVKAVTECSYVSNQIQNSQTEIDLLKAVEEQFNCTGICSSDVIYYFSDINRGPPSSTSGCYKPLKDLLLKIIGGVFIFVVISTVFSFLNVLFGFVNCCCAKYDTPQNQNQDQNAQNQQDISSIQMSVRKPLNPQPQAQQSGYNLQNIAAHIPINQQNINAVQKNVGSAFNQLNHYLPQISHNKQNKLF

>Tetrahymena-2 XP_001011643 Tetraspanin family protein [Tetrahymena thermophila SB210]

FSKYLLINFSDMAGTCLKTILVICALVTIAAGGVGLYFTLTQLNGYKELWSQVNPDVDKYAMYSLCGFTGVTILTGLISLIGVLKRNRCLLLIFNILAIVLLGLFVAIGVFITIYTNNQFQDIKNITNCNNAPSDYNWLQQSNEFYQKVGILFCTQLCKCYVQNVQDFPPSTFDNKSVATIPSQGTTQIQVCPYAKQETQYDQYLSLVQFLENKFECSGICDPVPYYVFTDINRGPPTYTSGCKQRVQDFFEKYGNIVRVVAFSIGGFFFLQIILAIWLCCIKKSNGENDYYSRLAQY

>tetrahymena-3 Q23XS5|Q23XS5_TETTH Tetraspanin family protein - Tetrahymena thermophila SB210

MSFKFLQRLVEITTFIILLLGIFWIVFSAILFSKDKDVTDTSKYDIYRGALALGIAFGILLVIWAIIGLIGACKKNNCLLGTFNFGIFIFLLVSIAILVLSIIVAANLPDYKDDKNCTQKSLLIDLKNLNNQSYQALCQNSCQCNFKGSSIQALQQGIINYSGTQGAQKVQDCQVFSSFGLSNQNSNSDLLKAIEDTFDCSGFCSINTYYVFSNVNNGFPNKDCKVELLNFIDDNNKRVIIAAAVITFFLFLTFILSICLCVKKPKGENFYERNQVGQYNNK

>tetrahymena-4 Q23XS4|Q23XS4_TETTH Tetraspanin family protein - Tetrahymena thermophila SB210

MNYKTLQRLVEFSTVLLLLLGIFWVIFAAIIYSKNKDISNVSEYDSYRGALALGIAFGIILILWALLGLLAACKKYNCLLGTYNVGVFILLLVSLAILIISIVVTAYIKDYKNDTDCTKESLLKDLKKLNDQSYQMLCNVNCKCNYKGSNLDALSHGIVNYSSSQGVLRAQDCQGFSSIGVSNLKSYSDLLRSVEDTFNCSGFCNTNNYYVFSDVNNGFPNKDCKKEVIDFIDTNNTRIIIASAIITFFFLVSFILVICLCCKKPKGENFYQRTGEGQYKA

>tetrahymena-5 Q24FE3|Q24FE3_TETTH Tetraspanin family protein - Tetrahymena thermophila SB210

MNLKLLKKSVIITNSLLLLVGLFFIIYPAILYQQYAYTTYTNNLFSGILIFGIVLGVFLILWTLFGVLAAQKNNTMIQAAYNVGIIMLFLLSIAILAISCIVQYEVPSYKNDEKCTQQSILIQLQQLNHKSSLTLCQNDCLCNFKGTQEEADKLGINNFNHKDSSPVRVQDCQIFEDFQFENQSENSEVLKFLEELFGCSGFCSQNSYYLFSDLNDGIPNGDCKVHVINFVEDNIIAIVISSSFITVLLLVSLIFNILFYLELLKSRYSSK

>tetrahymena-6 6Q24C21|Q24C21_TETTH Tetraspanin family protein - Tetrahymena thermophila SB210

MYHPINEPNQKKSCLQVYISWTSYINLVISIAALAFGIYIGVKNKSVWSNSSFDWNKTLIALLIAGSVFLLLASIAGICGGRKQNKCCIFIFQILSIILALFFLVAGFIFLGLTDSHFTDIKDSTCQDKGSNGTEIFYYGQEAYNSAFSLFCTQRCPCKVTNTEVKSYITKNYIQMVPFMQDNGSVKVQDCQDYKTVFSNSDVKNQANWLQSLEDSFDCTGMCKKPANIYFFSDINNGIPDNDTCKEKFQDFVQSYGRVAYIVGLIVGAYLLINAILAFCLCCRKKNSGQSLYERFANY

**DISCICRISTATES**

>Trypanosome-2 *Trypanosome brucei* XP_845049.1

MKVESPHSNYQPLNSEEGRWFHRIRNFIMGINVFLMAFAVSGLVVGFIELDEIDSAIREICSSCQHAHVIYMSSFGALLLLSFLGFVALHTRKRCLRILNTTCLVLVFIPLVFGSVLYVLMSTEHINMQYGWNLVVAERSDDMCKLELQWKCSGWNKLCATHSTIGLIDLKPLEGTEKENIINNLSVVTNTTSYCSGADEICACPICTEDDQKYIDKFDQTCEMVVMGALRSHLIVFLFVSLCVVVLTGAGIVVSVAYPHAES

>Trypanosome-1 *Trypanosoma cruzi* XP_805082 and ESTs

MHAKLLGISTPEKGRPQEMDMYVPDEEVDWTSDAYNRLYGERPPQNRSYNCTYHRFSTACFSAFLILLGAMSAVIAVGGSKSKELNFCHDCKTLILFLLVPGIFFVIMGIVGAVSAWRETKLCSALFSLLLVIAAFLVLGTGVTVVIAYTQVVTSDDSLALYWERAVSNAPSRICDLQCWLHCSGFASTHCCVSNVTAERLPDVSPCYLLAEDGVTTLDPNTLQPVSWPSLTCAPRCNSSNIYNATCKEPLQEFFMNFFPLTTGGLFALGLVFLGFASLAVYRITVTGGRTSYLRYEY

**EXCAVATES**

>Trichomonas Trichomonas vaginalis XP_001327241 ESTs CV213702 CV213701

MARTIIGIVNLVVLVIIAIVAFVCRSAIKSINIDQIKAFSKTTYFLFAVLGVVILGCLIGFSLICFGSVKCCRVSYAIFYIIVIIAEIIFVAVAIKYTKNVDKEAGDYWAESGVNDSVKDGVKKIEKAGKCCGYDKPYANNSAECGFQPSYENTTTCRQLVVDKINANKKRAMIAGIIVIVFQLILFIYAVWYAFCYEDPDSTQKEGITYV
