# Supplementary figures and images for: Intron Evolution: Testing Hypotheses of Intron Evolution Using the Phylogenomics of Tetraspanins
Source: PLoS One. 2009 Mar 5;4(3):e4680. doi: 10.1371/journal.pone.0004680 (PMC2650405; doi:10.1371/journal.pone.0004680)

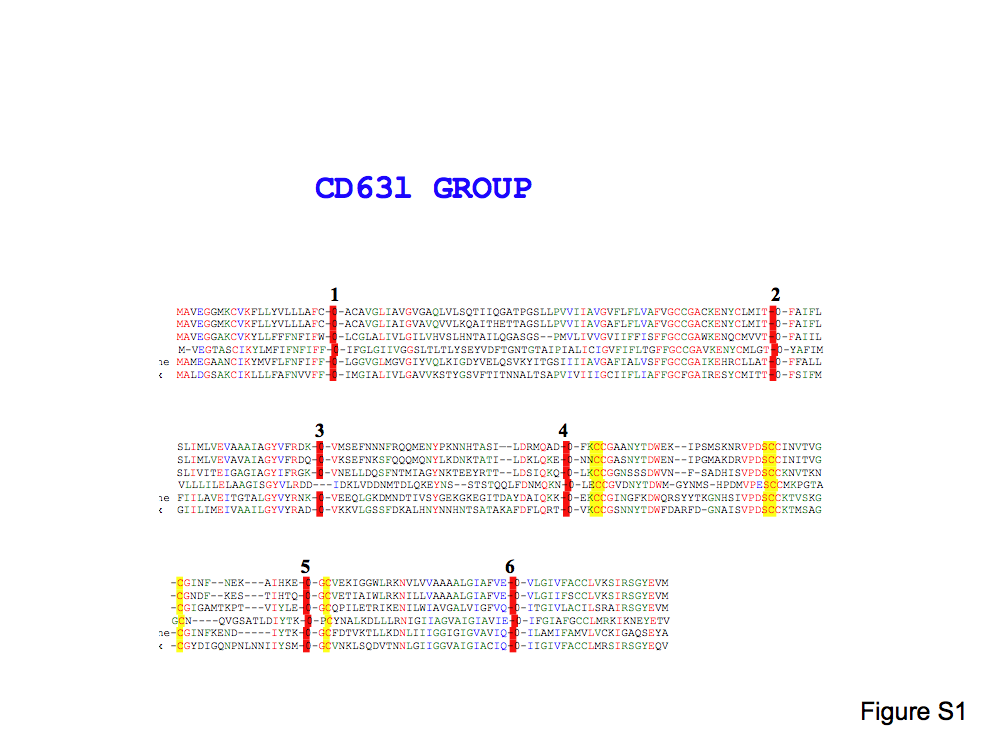

Supplement: Figure S1 — Intron junction analysis of the CD63L group. Alignment of full length representative CD63 tetraspanin from human (Hs), mouse (Mm), and zebrafish (Dr) with Sea anemone and Trychoplax CD63-like tetraspanins. The presence of an intron is shown within the amino acid sequences by a number, shaded in red, indicating the intron phase (0 is between codons, 1 is between the first and second position of a codon and 2 is between the second and third position). Ancestral introns 1–6 are numbered above the alignment. Cysteines in the variable subdomain in the LEL loop are shaded in yellow. (0.25 MB TIF) [file pone.0004680.s002.tif]

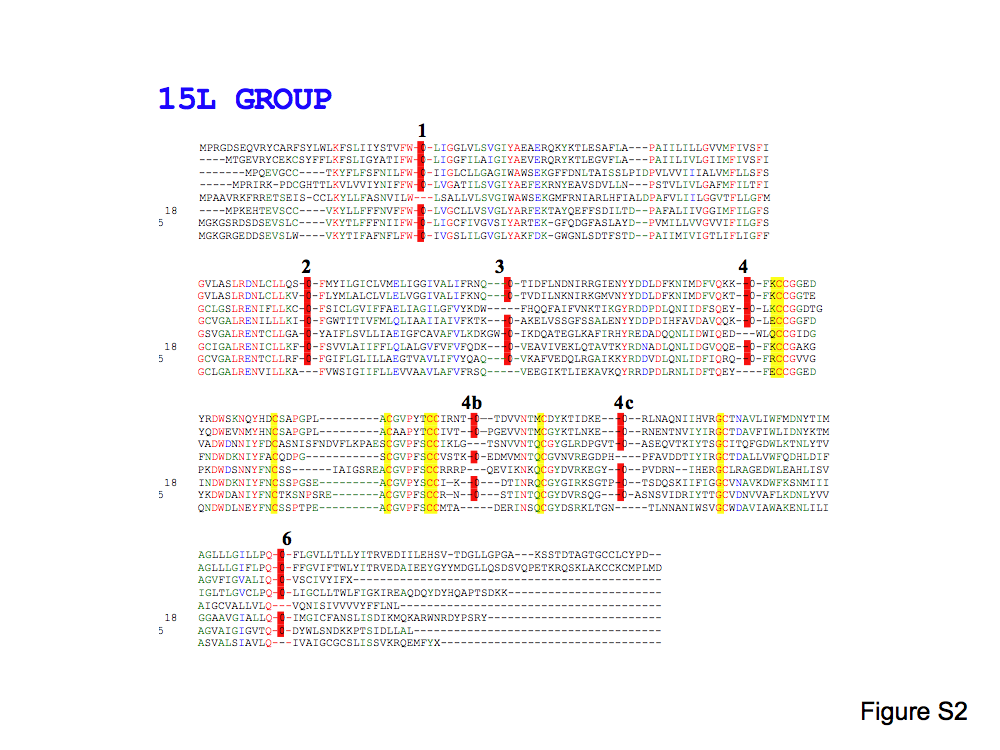

Supplement: Figure S2 — Intron junction analysis of the TSPAN15L group. Alignment of Deuterostomes (Mm, Dr, Ci, and Sp) and Protostomes (Dm) TSPAN15L proteines with Sea anemone, Trychoplax and Sponge tetraspanins of the TSPAN15L group. Ancestral introns 1–4, and 6 plus introns 4b and 4c characteristics of this group of tetraspanins are numbered above the aligned sequences. Cysteines in the variable LEL loop domain and introns positions are marked yellow as in Figure S1. (0.34 MB TIF) [file pone.0004680.s003.tif]

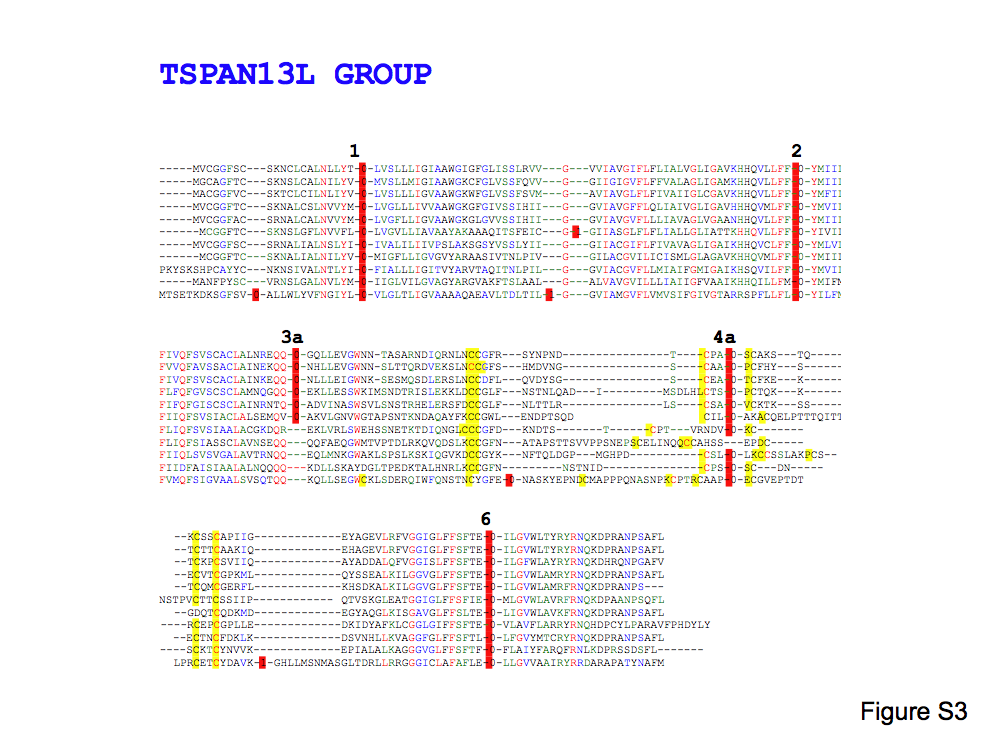

Supplement: Figure S3 — Intron junction analysis of TSPAN13L group. Alignment of Deuterostomes (Mm, Dr, Ci, and Sp) and Protostomes (Dm) TSPAN13L proteines with Sea anemone, Trychoplax and Monosiga tetraspanins of the TSPAN15L group. Characteristic introns of this group (1, 2 and 6 plus 4a) are numbered above the aligned sequences. Cysteines in the LEL loop and introns positions are marked as in Suppl. Fig 1. (0.37 MB TIF) [file pone.0004680.s004.tif]

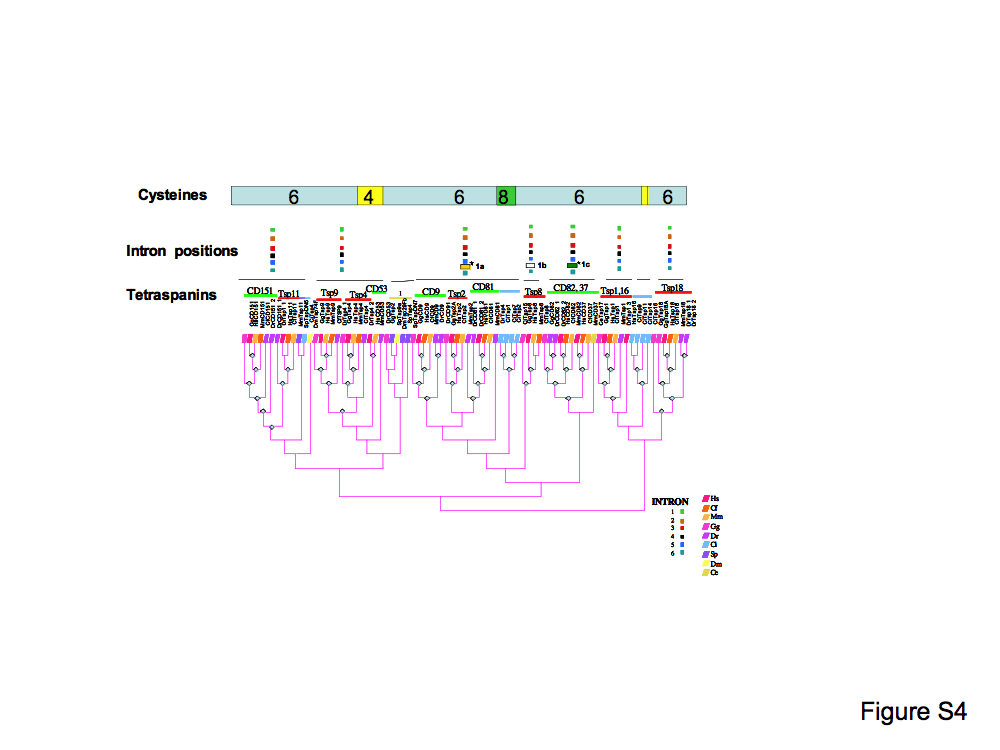

Supplement: Figure S4 — Evolution of the Intron structure in tetraspanins supports several nodes in animal phylogeny. Intron structures are represented above the tetraspanins from the cluster we called CD group in Garcia-Espana et al [18]. Cysteine numbers in the varaiable LEL domain are shown in boxes above the intron structures. Species are designated by coloured boxes with a legend for the species designation given. (Species abbreviation are: Hs, human; Cf, dog; Mm, mouse; Gg, chicken; Dr, zebrafish; Ci, sea squirt; Sp, sea urchin; Dm, drosophila; Ce; C. elegans. (0.16 MB TIF) [file pone.0004680.s005.tif]

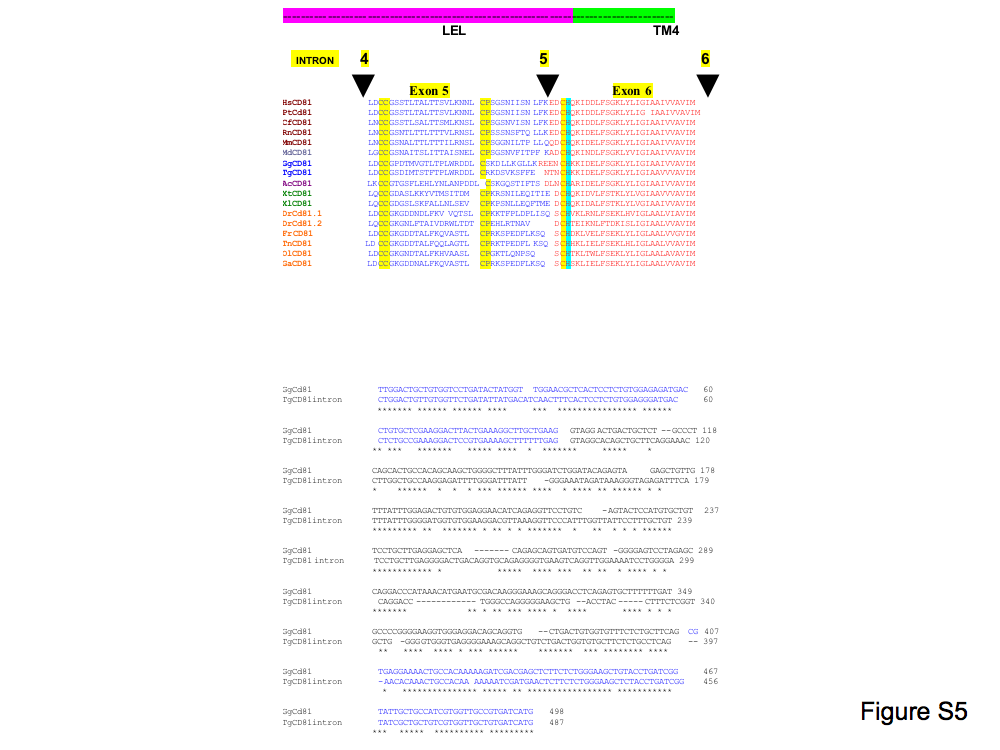

Supplement: Figure S5 — Junction analysis of the CD81 group. (A) Protein alignment of the full exon protein sequences between introns 4 and 6 of several CD81 tetraspanins. Cysteines are shaded in yellow. (B) Clustal W DNA alignment of the above exons 5 and 6 in blue letters plus the intron sequence between them in black letters of CD81 tetraspanins from Aves, chicken (Gg) and zebra finch (Tg). Other species: Hs, human; Pt, chimpanzee; Cf, dog; Rn, rat; Mm, mouse; Md, opossum; Ac, green anole lizard; Xt and XL, frogs; Dr, zebrafish; Fr and Tn, puffer fish; Ol, medaka; Ga, stickleback. * indicates indentical bases in both sequences. (0.24 MB TIF) [file pone.0004680.s006.tif]

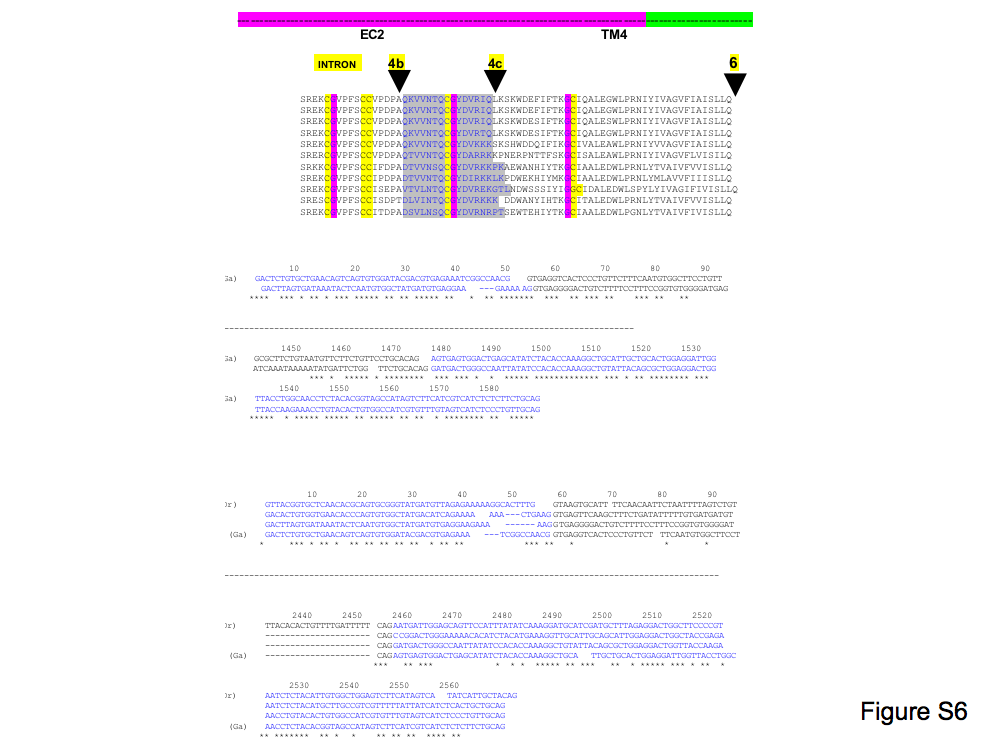

Supplement: Figure S6 — Junction analysis of theTSPAN14 group. (A) Protein alignment of the exon protein sequences between introns 4b and 6 of several TSPAN14 tetraspanins. Intron/exon junctions are indicated with arrowheads. (B) Clustal W DNA alignment of exons sequences in blue and partial intron sequences in black of the TSPAN14 sequences from fish stickleback and medaka. (C) Alignment of sequences from zebrafish, fugu, medaka and stickleback.Dashes indicate intron sequence not shown. Species are designated as in S5. (0.24 MB TIF) [file pone.0004680.s007.tif]
